# Climatic niche evolution and niche conservatism of *Nymphaea* species in Africa, South America, and Australia

**DOI:** 10.1186/s12870-024-05141-1

**Published:** 2024-05-30

**Authors:** John M. Nzei, Norberto Martínez-Médez, Virginia M. Mwanzia, Joseph K. Kurauka, Qing-Feng Wang, Zhi-Zhong Li, Jin-Ming Chen

**Affiliations:** 1 Aquatic Plant Research Center, Wuhan Botanical Garden, Chinese Academy of Sciences, Wuhan, 430074 China; 2 University of Chinese Academy of Sciences, Beijing, 100049 China; 3 Departamento de Zoología, Escuela Nacional de Ciencias Biológicas del Instituto Politécnico Nacional, Ciudad de México, México; 4 School of Agriculture Technical Studies and Natural Sciences, Lukenya University, P.O Box 90-90128, Mtito Andei, Kenya; 5 School of Agriculture and Environmental Sciences, Kenyatta University, P.O. Box 43844-00100, Nairobi, Kenya; 6 Hubei Key Laboratory of Wetland Evolution & Ecological Restoration, Wuhan Botanical Garden, Chinese Academy of Sciences, Wuhan, 430074 China

**Keywords:** *Nymphaea*, Climate change, Niche evolution, Phylogenetic conservatism

## Abstract

**Background:**

Interest in the evolution of climatic niches, particularly in understanding the potential adaptive responses of species under climate change, has increased both theoretically and within macroecological studies. These studies have provided valuable insights into how climatic traits of species influence their niche evolution. In this study, we aim to investigate whether niche conservatism plays a role in the species diversification of *Nymphaea*, a group of aquatic plants with a cosmopolitan distribution that is facing severe habitat loss. We will use climatic models and phylogenetic data for 23 species to reconstruct *Nymphaea*’s niche evolution, measure niche overlap, and assess disparity through time while testing for evolutionary models.

**Results:**

There was a lot of overlap in niches both within and between groups, especially for species that can be found in many places. The breadth and peaks of the niche profile varied depending on the bioclimatic variables, which suggested that the species evolved differently to cope with changes in climate. The analysis also showed that evolutionary changes happened across the phylogeny, with weak to moderate signals. The morphological disparity index (MDI) values indicated that there were disparities within subclades over time but not between or among them. Niche reconstruction and evolution analysis revealed both convergent and divergent evolution among various variables. For example, *N. immutabilis*, *N. atrans*, *N. violancea*, and *N. nouchali* evolved towards intermediate temperatures for bio2 and bio3 (isothermity) while moving towards extreme temperatures for bio8 and bio9 (wettest and driest average quarterly temperatures).

**Conclusion:**

Our study will improve our understanding of how changes in climatic niches are potentially driving the evolution of *Nymphaea*. It has significant scientific implications for the limits, assemblages, evolution, and diversification of species. This information is crucial for the ongoing efforts of conservation and management, particularly considering the inevitable effects of climate change.

**Supplementary Information:**

The online version contains supplementary material available at 10.1186/s12870-024-05141-1.

## Background

The threat of climate change leading to habitat loss for various species highlights the urgent need for biodiversity and species habitat conservation [1–4]. The availability of data from species and ecological distribution models has significantly increased, allowing for well-informed decisions regarding species’ responses to climate change. It is crucial to comprehend how species adapt to diverse environments in light of climate fluctuations [5].

Phylogenetic niche conservatism (PNC) is an intriguing concept that investigates how species that are closely related share niche characteristics and preserve the attributes of their fundamental niche over time [6, 7]. This knowledge is not just theoretical, but it also has practical implications. PNC models offer valuable information that can guide decision-making processes related to species’ reactions to climate change. They provide a fast and cost-effective approach to developing conservation strategies. To test the assumption that phylogenetically closely related species share climatic niche requirements compared to distant ones, researchers have employed the PNC framework. Over evolutionary time, adaptation to different environmental conditions can result in trait divergence within a lineage [7]. This variation in trait evolution can lead to differences in patterns, rates, and modes of trait evolution. Macroevolutionary approaches play a pivotal role in our understanding of species niches. By evaluating the relative adequacy of different models of continuous trait evolution [8–10], such as Brownian motion (BM) and Ornstein-Uhlenbeck (OU) [10, 11], and considering phylogenetic signal indices such as Pagel’s λ and Blomberg’s K as a test for confirmation of PNC, we can delve deeper into the evolution of species niches [12]. Understanding the evolution of climatic niches is not just an academic pursuit but a crucial step toward biodiversity conservation. It helps us comprehend how climate has shaped the speciation process and species distribution over time. This knowledge is of utmost importance for the conservation of biodiversity as it directly affects the management and survival of species in present and future scenarios. Over the past million years, the Earth has experienced significant climatic variations [13], which have played a fundamental role in shaping the geographic distribution and diversification patterns of species.

The genus *Nymphaea* L., which belongs to the well-known plant family Nymphaeaceae (commonly known as water lily), is the most diverse, with an estimated 40–45 species [14]. The genus originated from a common lineage approximately 38 million years ago in the Eocene period. This gave rise to three distinct lineages: the subgenera *Hydrocalis* and *Lotos* (i), *Brachyceras* and *Anecphya* (ii), and *Nymphaea* (iii) [14]. Within these subgenera classifications, the majority of species occupy unique ecological habitats and ranges. For example, the subgenera *Hydrocalis* and *Lotos* are found in the Neotropics and Paleotropics of South America and Africa. *Anecphya* is native to Australia, while *Nymphaea* is recorded in Central and North America, Europe, and temperate Asia. *Brachyceras* is widely distributed across Pantropical regions, including Central and North America, Europe, Africa, Australia, and temperate and tropical Asia [15]. These species demonstrate distinct distribution patterns associated with ecological adaptation and recent climate change, which pose a significant threat to their habitats. *Nymphaea* species are considered cosmopolitan, indicating their wide distribution throughout various regions worldwide. These regions experience different climate change effects, which, along with other ecological factors, are likely to impact species distributions and patterns.

Previous studies have investigated *Nymphaea* species by analyzing various genetic regions, including the nuclear region (ITS) and the noncoding region (*trn*T*-trn*F), as well as the coding regions (*rbcL*, *rpl16*, and *matK*). Some studies have analyzed the entire genome [16–18], while others have used morphological data in combination with genetic analysis [19–25]. Moreover, researchers have assessed the genetic diversity of *Nymphaea* species [26, 27] and examined the suitability of their habitats [26, 28–30]. However, the climatic niche evolution and niche conservatism of *Nymphaea* species remain unexamined. Therefore, we utilized a phyloclimatic modeling approach that integrated phylogenetic information and environmental niche models (ENMs) derived from bioclimatic data to understand the niche evolution of the *Nymphaea* species. This approach has successfully explored various evolutionary questions and evaluated the potential responses of the organisms to future climatic changes [5, 31, 32]. Such studies are becoming increasingly popular due to the significant loss of species biodiversity caused by global warming [8].

In this study, we aim to explore the effects of climate change on the evolution and distribution of *Nymphaea* species in key areas of Africa, Australia, and South America. To achieve this, we will utilize phylogenetic data and species distribution models (SDMs) to (i) perform phylogenetic signal analysis, (ii) assess ancestral climatic tolerances, (iii) evaluate niche overlap, (iv) fit different macroevolutionary models, (v) evaluate ecological niche disparity through time (DTT), and (vi) evaluate niche reconstruction and the evolution of *Nymphaea* species. By examining the influence of climatic factors on the evolutionary dynamics of lineages and species across space and time, We will determine whether convergent or divergent events occurred during the evolution of species niches. Our study will not only enhance our understanding of the patterns of evolutionary diversification and adaptation in *Nymphaea* species but also provide insights into how different clades can colonize the same areas and develop similar habitat requirements.

## Results

### Phylogenetic reconstruction

Using a subset of molecular data from the ITS and *trn*T*-trn*F regions of *Nymphaea*, a total of 26 ingroup taxa and one outgroup, 1967 molecular characteristics were analyzed, revealing a phylogenetic structure consisting of three lineages and five clades. The first clade included *N*. subg. *Nymphaea*, while the second and third clades comprised *N*. subg. *Lotos* and *N*. subg. *hydrocalis*, respectively. The fourth and fifth clades were *N*. subg. *Brachyceras* and *N*. subg. *Anecphya*. Phylogenetic analysis suggested that the divergence of the genus *Nymphaea* from its sister genus occurred in the late Paleogene (Oligocene), with the most recent split occurring in the mid-Neogene (Fig. S1).

### Ecological niche modeling

The ecological niche models encompassed the entire accessible area of the genus. The models demonstrated good accuracy, with AUC values ranging from 0.731 for *N*. *lotus* to a maximum of 0.999 for *N*. *atrans* and *N*. *georginae*. The COR values varied from 0.059 to 0.700, with *N*. *ampla* and *N*. *atrans* showing the lowest and highest values, respectively (Table 1). The models indicate that Africa and South America have greater habitat suitability and potential distribution, suggesting a close environmental relationship between the two continents. Introduced species such as *N*. *odorata* and *N*. *mexicana* show potential suitability across continents, while the Australian *Anecphya* group remains endemic based on the models. The maps showed that most species have limited geographic ranges compared to those in Africa and South America (Fig. S2), which indicated restricted dispersal opportunities.

**Table 1:** Model fitness evaluation and the threshold at which species absence is assumed

Species	AUC	COR	maxSS
*N. alba*	0.991	0.485	0.020
*N. amazonum*	0.886	0.150	0.011
*N. ampla*	0.825	0.059	0.031
*N. atrans*	0.999	0.700	0.071
*N. carpentariae*	0.992	0.381	0.024
*N. elleniae*	0.996	0.451	0.048
*N. georginae*	0.999	0.516	0.077
*N. gigantea*	0.960	0.393	0.006
*N. hastifolia*	0.996	0.438	0.042
*N. heudelotii*	0.907	0.063	0.016
*N. immutabillis*	0.975	0.387	0.004
*N. jamesoniana*	0.917	0.077	0.043
*N. lingulata*	0.928	0.199	0.021
*N. lotus*	0.731	0.152	0.011
*N. macrosperma*	0.994	0.519	0.013
*N. mexicana*	0.989	0.396	0.021
*N. micrantha*	0.920	0.253	0.007
*N. nouchali*	0.801	0.307	0.024
*N. odorata*	0.995	0.216	0.111
*N. pubescens*	0.998	0.697	0.019
*N. pulchella*	0.917	0.338	0.007
*N. rudgeana*	0.860	0.100	0.016
*N. violancea*	0.978	0.594	0.002

AUC; Area under the curve, COR; biserial point correlation, and maxSS; maximum specificity and sensitivity threshold

**Fig. 1:**
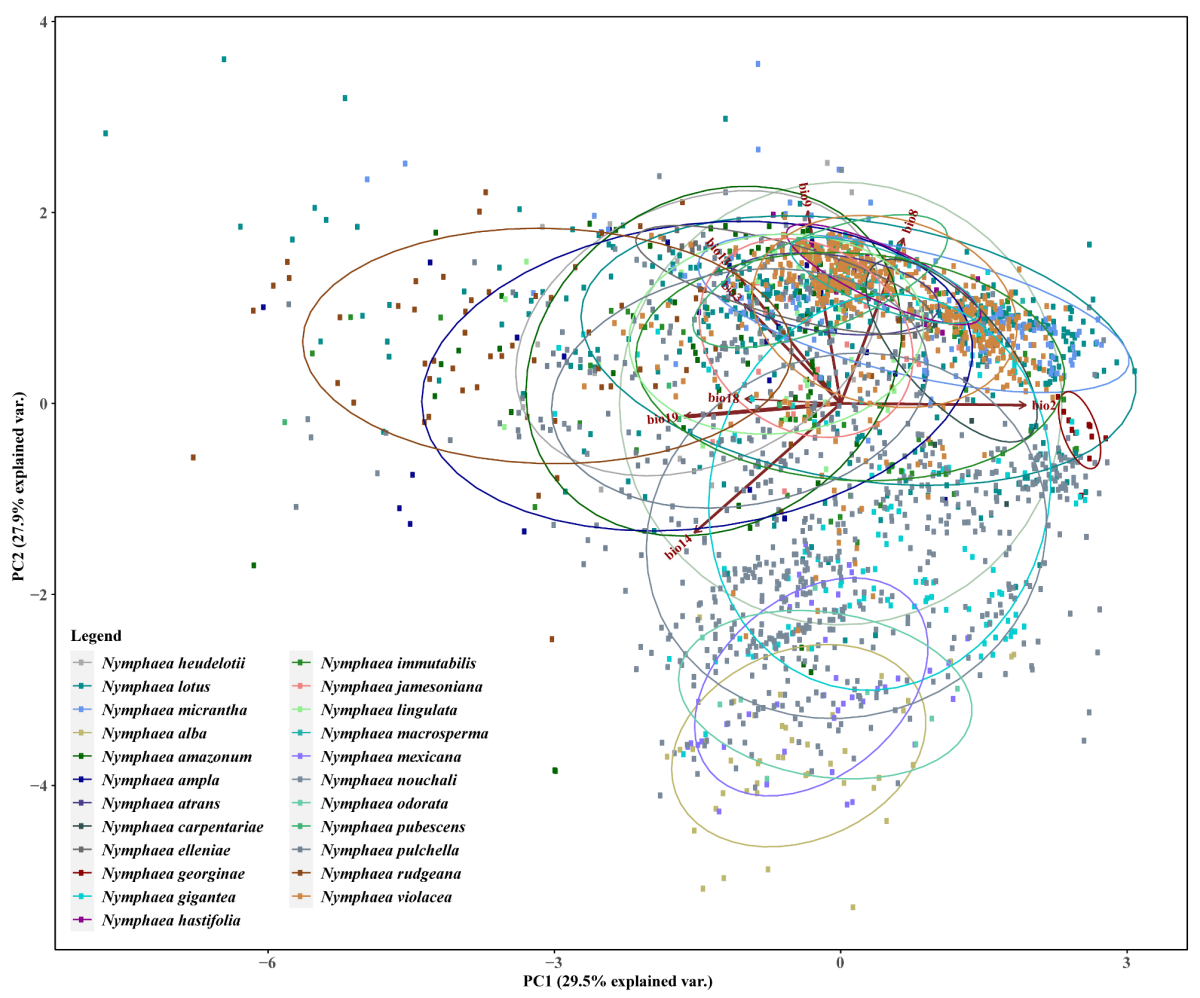
Principal component analysis (PCA) for the 26 *Nymphaea* species habitat niche variation

**Fig. 2:**
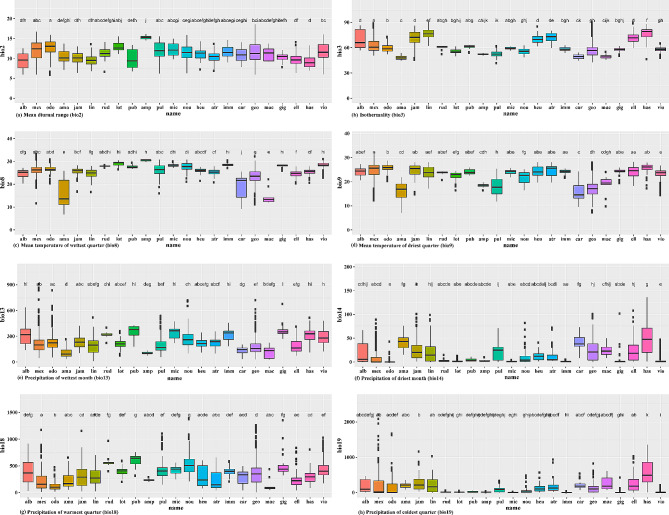
Analysis of variance (ANOVA) and post hoc test using Turkey Honest Significant Differences (Tukey HSD) for *Nymphaea*s’ niche evaluation. Significantly different (alpha = 0.05) groups are indicated by color and letter for each variable. The abbreviations in the tree represent: alb = *N. alba*, odo = *N. odorta*, mex = *N. mexicana*, lot = *N. lotus*, jam = *N. jamesoniana*, ama = *N. amazonum*, rud = *N. rudgeana*, lin = *N. lingulata*, heu = *N. heudelotii*, mic = *N. micrantha*, pul = *N. pulchella*, amp = *N. ampla*, nou = *N. nouchali*, ell = *N. elleniae*, vio = *N. violancea*, has = *N. hastifolia*, atr = *N. atrans*, imm = *N. immutabilis*, car = *N. carpentariae*, gig = *N. gigantea*, mac = *N. macrosperma*, geo = *N. georginae*

The variance in the realized niche accounted for 29.5% of the variance in PC1 and 27.9% of the variance in PC2, according to the PCA result, demonstrating the presence of overlapping niche spaces for most species (Fig. 1). *Nymphaea georginae* exhibited a limited range size and less overlap compared to the other species. Notably, three species, namely, *N*. *odorata*, *N*. *mexicana*, and *N*. *alba*, were clearly distinguished from the others, as they shared a similar environmental space below − 2 on the y-axis. The bioclimatic variables bio2 and bio19 made the most remarkable contributions to PC1 (20.92% and 17.71%, respectively), while bio9 and bio8 contributed the most to PC2 (25.98% and 22.29%, respectively) (Table 2). Although all the other variables performed reasonably well, the contributions of the bio2 and bio18 variables to PC2 were relatively low (Table 2). The contributions of bioclimatic variables varied significantly among the species (Fig. 2). In comparisons within clades, some species in the *N*. subg. *Anecphya* showed no significant differences in bioclimatic variables, such as *N*. *atrans* and *N*. *gigantea* in bio9 and *N*. *immutabilis*, *N*. *gigantea*, and *N*. *violancea* in bio8.

**Table 2:** Relative contributions of the eight bioclimatic variables in principal component analysis 1 and 2

Bioclimatic variables	Code	PC1	PC2
Mean Diurnal Range (°C)	bio2	20.923	0.225
Isothermality (°C×100)	bio3	10.92	13.427
Mean Temperature of Wettest Quarter (°C)	bio8	7.096	22.29
Mean Temperature of Driest Quarter (°C)	bio9	3.717	25.983
Precipitation of Wettest Month (mm)	bio13	12.289	18.22
Precipitation of Driest Month (mm)	bio14	16.543	17.452
Precipitation of Warmest Quarter (mm)	bio18	10.806	0.618
Precipitation of Coldest Quarter (mm)	bio19	17.706	1.784

### Ecological niche overlap

**Fig. 3:**
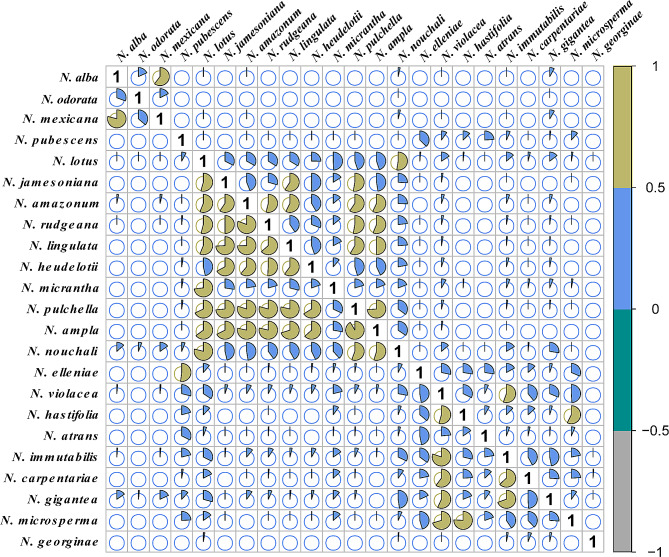
Schoener’s *D* (above diagonal) and Warren’s *I* (below diagonal) pairwise ecological niche overlap. Indexes > 0.5 indicate more overlap

The analysis of niche overlap revealed that some species exhibited high niche overlap values, while majority displayed low to moderate overlap niche values within and among different clades (Fig. 3; Table S1). Among species pairs within the same clade showing high overlap values, we observed *N. lingulata* and *N. jamesoniana* (0.742; *N.* subg. *Hydrocalis* clade), *N. pulchella* and *N. ampla* (0.723; *N.* subg. *Brachyceras*), *N. rudgeana* and *N. amazonum* (0.808; *N.* subg. *Hydrocalis*), and *N. hastifolia* and *N. macrosperma*, as well as *N. violancea* and *N. immutabilis* (0.802 and 0.759; *N.* subg. *Anecphya* respectively). Similarly, *N. hastifolia* and *N. macrosperma*, *N. violancea*, and *N. immutabilis* from Australia (*N.* subg. *Anecphya*) exhibited overlap values greater than 0.5. Conversely, the niche overlap between species from different clades was greater for *N. rudgeana* (*N.* subg. *Hydrocalis*) with *N. ampla* and *N. pulchella* (*N.* subg. *Brachyceras*) (0.788 and 0.793, respectively), both clades from Central and South America. Additionally, *N. lotus* (*N.* subg. *Lotos*), primarily found in Africa, showed significant overlap with *N. micrantha* and *N. nouchali* (*N.* subg. *Brachyceras*) from South America (0.730 and 0.789, respectively). These findings suggest varying levels of niche conservatism and divergence among species pairs within the group; however, ecological divergence predominate.

### Predicted ancestral tolerance and niche occupancy

**Fig. 4:**
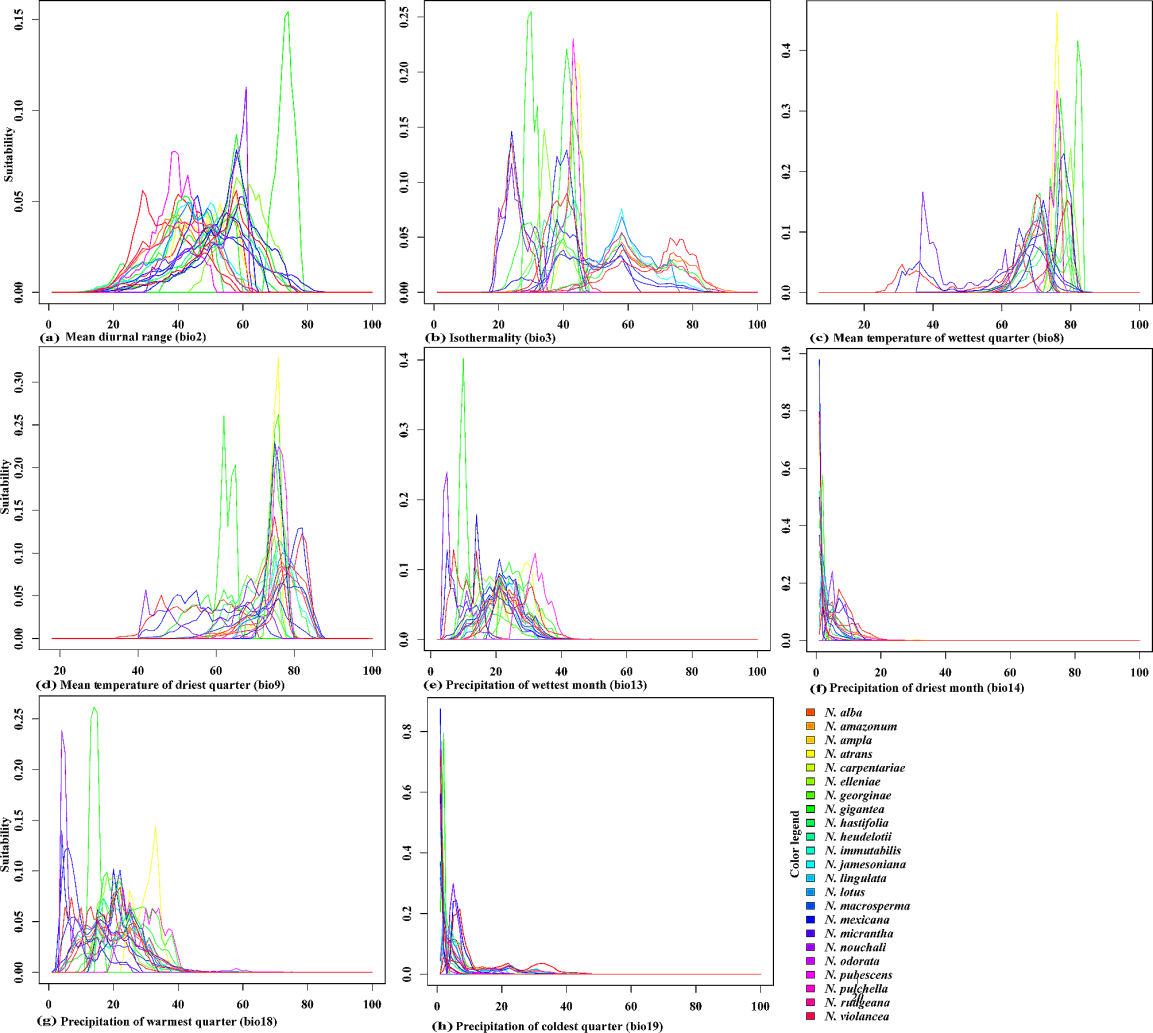
Predicted niche occupancy (PNO) profiles in the eight bioclimatic variables of the *Nymphaea* species. The vertical axis represents total suitability and horizontal variable values, with overlapping peaks indicating preference of similar climatic aspects

The ENMs were applied to assess the phylogenetic history of niche evolution by analyzing the PNO profiles. The contribution of each bioclimatic variable to the heterogeneity of the species distributions was visualized (Fig. 4). For example, the isothermity (bio3) variable showed that different *Nymphaea* species occupied distinct regions of the parameter space. *N. violancea*, *N. odorata*, and *N. mexicana* were clustered on the right side, with values ranging from approximately 45 to 55. *N. elleniae*, *N. carpentariae*, *N. georginae*, and *N. gigantea* occupied the range of 50 to 65, while *N. jamesoniana* and *N. lingulata* had values of 60 to 85, with some overlapping distributions. *Nymphaea nouchali* exhibited a more comprehensive range, ranging from 45 to 85. There were isolated peaks in the distribution of certain species for specific bioclimatic variables, such as *N*. *odorata* in bio2, bio8, bio13, and bio18; *N*. *atrans* in bio9 and bio18; and *N*. *georginae* in all variables except bio14 and bio19. These peaks indicated unique aspects of their distribution compared to those of other species. Notably, bio8, bio14, and bio19 tended to influence the evolutionary history of the species despite differences in their ecological distributions. The diverse PNO profiles revealed distinct adaptations to precipitation and temperature, as well as niche partitioning within the clades. For instance, *N. odorata*, *N. macrosperma*, and *N. alba* exhibit a tolerance for precipitation levels less than approximately 200 mm, while *N*. *pubescens* and *N*. *pulchella* thrive with precipitation ranging between 200 and 400 mm in bio13. Overall, the PNO profiles for temperature and precipitation significantly influenced the clustering of taxa within similar parameter ranges (Fig. 4). Furthermore, the PNO analysis sheds light on the evolutionary radiation of *N*. *nouchali* and *N*. *lingulata*, as they have adapted to a wide range of bio3 values, and *N*. *nouchali* also demonstrates versatility in its bio8 PNO profile.

**Fig. 5:**
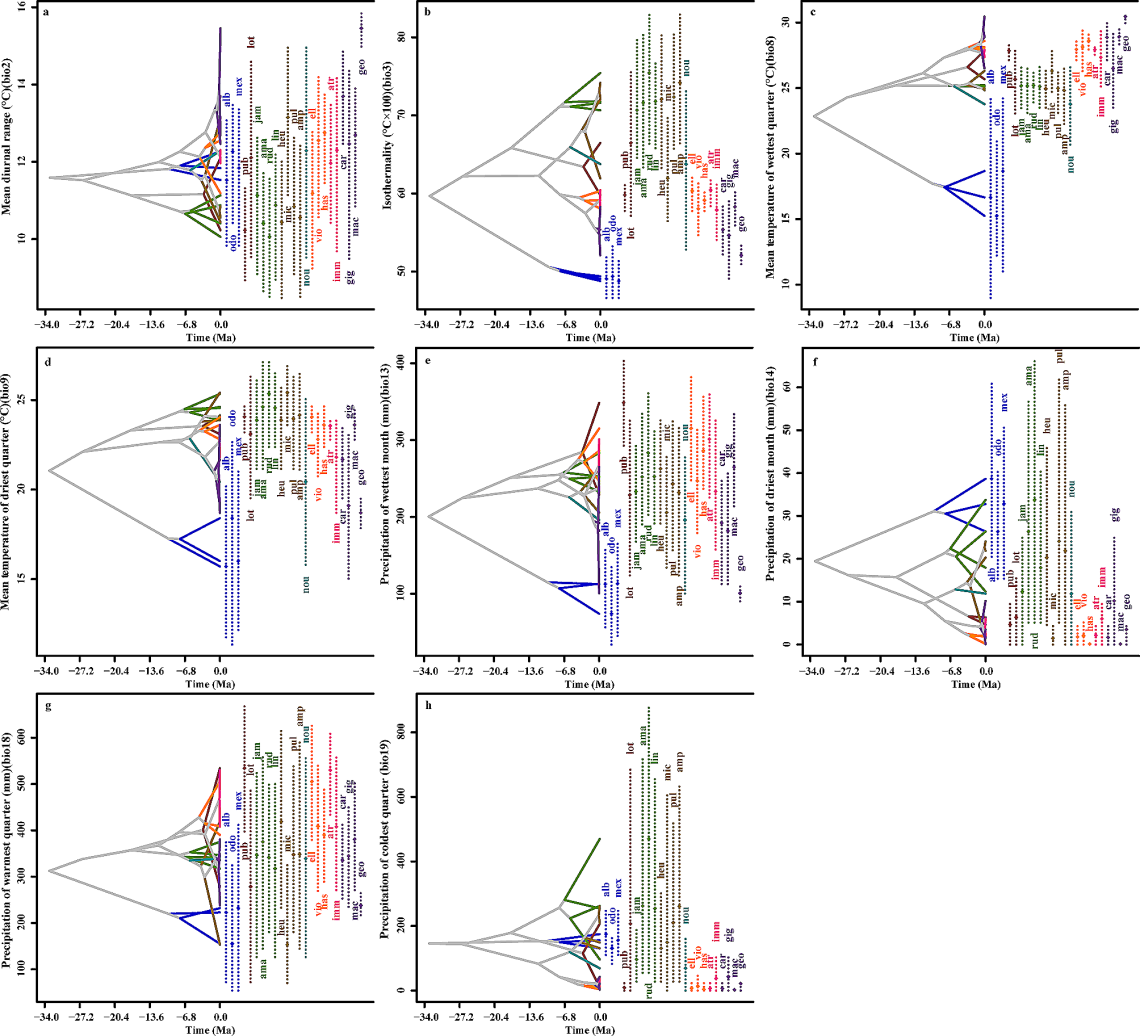
Evolution of climatic niche tolerance for *Nymphaea* species for the visualized eight bioclimatic variables. Y axis indicate variable values and X divergence time, while crossing branches indicate convergent evolution

The PNOs of bio18 indicate a diverse array of *Nymphaea* species radiation compared to other projected ecological spaces, such as bio13, where clear separation in precipitation is observed between the first and second clades (Fig. 5). Within bio13, species such as *N. alba*, *N. odorata*, and *N. mexicana* exhibit divergent evolution across a wide range of ecological space, occupying both low and high values of the variable. However, these species remain conserved within their respective clades, with little to no divergence observed among species from the same clade. In contrast, convergent evolution is observed through the branching patterns among different species in bio2 and bio18, particularly within the first clade. The taxon distribution within this clade demonstrates a tolerance for low values of these bioclimatic variables, enabling these species to be easily introduced to new geographical areas. While most of the bioclimatic variables support a wide distribution range for the species, bio8 limits the distribution of all clades except *N.* subg. *Nymphaea*, while bio3, bio14, and bio19 contributed to the restricted distribution of *N.* subg. *Anecphya* in Australia.

### Phylogenetic signal testing for niche conservatism

The bioclimatic variables tested non-provided substantial evidence in support of the PNC hypothesis, as they displayed null phylogenetic signals (K < 1) with distribution of variance within clades, and only bio8 show a weak phylogenetic signals (0.884, *P* = 0.001). Additionally, only bioclimatic variables bio3, bio8, bio14, and bio19 showed evidence of correlation among species under a BM trait evolution process (Pagel’s *λ* range from 0 (no correlation) to 1 (correlation), with *λ* values of 0.932, 0.912, 0.913, and 0.998 at *P* = 0.00 (Table 3). Among the alternative evolutionary models, only bio8 exhibited a lower AICc value, indicating a BM pattern of evolution (Table 4).

**Table 3:** Phylogenetic signal test based on Blomberg’s *K* and Pagel’s *λ* for the eight bioclimatic variables

Bioclimatic layer	Blomberg’s K		Pagel’s lambda (λ)			
	K	*P*	λ	logL	logL0	*P*
Mean Diurnal Range (°C)(bio2)	0.071	0.307	0.422	−36.027	2.738	0.098
Isothermality (°C×100)(bio3)	0.359	0.001	0.932	−70.495	21.433	0
Mean Temperature of Wettest Quarter (°C)(bio8)	0.884	0.001	0.912	−46.878	30.456	0
Mean Temperature of Driest Quarter (°C)(bio9)	0.102	0.157	0.733	−50.847	11.713	0.001
Precipitation of Wettest Month (mm)(bio13)	0.051	0.47	0.571	−127.41	5.277	0.022
Precipitation of Driest Month (mm)(bio14)	0.385	0.001	0.913	−83.366	13.968	0
Precipitation of Warmest Quarter (mm)(bio18)	0.062	0.32	0.22	−138.14	0.512	0.474
Precipitation of Coldest Quarter (bio19)	0.578	0.001	0.998	−134.11	18.92	0

**Table 4:** The comparative performance of the four alternative macro-evolution models for the eight bioclimatic variables

Bioclimatic layer	Model	InL	AICc	Parameters
Mean Diurnal Range (°C)(bio2)	OU	−39.017	85.298	3
	δ	−39.021	85.306	3*e*
	BM	−57.663	119.927	2***
	EB	−57.663	122.59	3***
Isothermality (°C×100)(bio3)	OU	−79.556	166.376	3
	δ	−79.743	166.75	3*e*
	BM	−84.393	173.387	2*
	EB	−84.393	176.05	3**
Mean Temperature of Wettest Quarter (°C)(bio8)	BM	−53.851	112.301	2
	OU	−53.139	113.542	3*e*
	δ	−53.738	114.74	3*
	EB	−53.851	114.964	3*
Mean Temperature of Driest Quarter (°C)(bio9)	δ	−58.375	124.013	3
	OU	−58.388	124.039	3*e*
	BM	−71.335	147.269	2***
	EB	−71.335	149.933	3***
Precipitation of Wettest Month (mm)(bio13)	δ	−132.958	273.179	3
	OU	−133.027	273.317	3*e*
	BM	−151.257	307.115	2***
	EB	−151.257	309.778	3***
Precipitation of Driest Month (mm)(bio14)	OU	−90.201	187.665	3
	δ	−90.337	187.937	3*e*
	BM	−94.288	193.177	2*
	EB	−94.288	195.84	3**
Precipitation of Warmest Quarter (mm)(bio18)	OU	−142.286	291.835	3
	δ	−142.288	291.838	3*e*
	BM	−152.222	309.045	2***
	EB	−152.223	311.708	3***
Precipitation of Coldest Quarter (bio19)	δ	−136.384	280.031	3
	OU	−136.5	280.263	3*e*
	BM	−138.23	281.059	2*e*
	EB	−138.23	283.722	3*

The initials represented; Akaike Information criterion for small sample size (AICc), Ornstein-Uhlenbeck (OU), Pagel’s delta (δ), Brownian motion (BM), Early burst model (EB)

### Accumulation of disparity through time

**Fig. 6:**
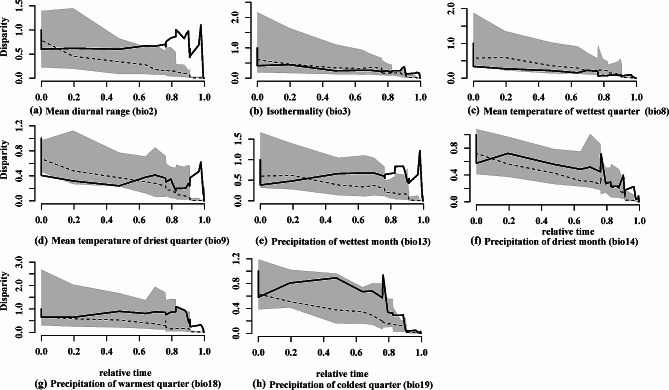
Relative disparity through time (DDT) accumulation plots of *Nymphaea* species climatic tolerances. The solid lines display distribution of relative disparity through time and dashed line unconstrained Brownian motion evolution model under 1000 replicates

Disparity through time (DTT) plots were generated to measure the extent of disparity within and among the clades (Fig. 6). The plots reveal a departure from the Brownian model of evolution, with most ecological disparities starting at values above 0.4 and some bioclimatic variables accumulating even greater disparities (bio2, bio13, bio18, and bio19). In contrast, other variables showed a decrease in disparity over time (bio3, bio8, bio9, and bio14). The DTT plots display a relatively stable and progressive disparity from the base of the topology (time 0) to the region containing recent tip topologies for bio18 and bio19. However, for other bioclimatic variables, there is variation in the progression shown by the overlapping lines of observed relative disparity (continuous line) and null model disparity (dotted line), such as in bio2, bio9, bio13, and bio14. The DTT plots for the climatic variables bio2, bio9, bio13, and bio18 demonstrate an accumulation of disparity within the subclades toward recent timeframes (divergence in recent nodes). These levels of disparity also fall outside the 95% confidence interval of null speciation for recent years. Generally, the DTT disparity for all climatic layers is concentrated within the subclades at a relative time between 0.5 and 1.0. Across all clades and bioclimatic variables, all the MDI values were positive, indicating that the distribution of disparity occurred within the subclades rather than among or between them (Table 5).

**Table 5:** Total Morphological Disparity Index (MDI) for the *Nymphaea* species phylogenetic tree

Bio climate variables	MDI values	avg.sq
Mean Diurnal Range (°C)(bio2)	0.6	0.330
Isothermality (°C×100)(bio3)	0.4	−0.036
Mean Temperature of Wettest Quarter (°C)(bio8)	0.2	−0.156
Mean Temperature of Driest Quarter (°C)(bio9)	0.3	−0.019
Precipitation of Wettest Month (mm)(bio13)	0.9	0.203
Precipitation of Driest Month (mm)(bio14)	0.7	0.114
Precipitation of Warmest Quarter (mm)(bio18)	0.7	0.316
Precipitation of Coldest Quarter (bio19)	0.9	0.287

### Climatic niche reconstruction and evolution

**Fig. 7:**
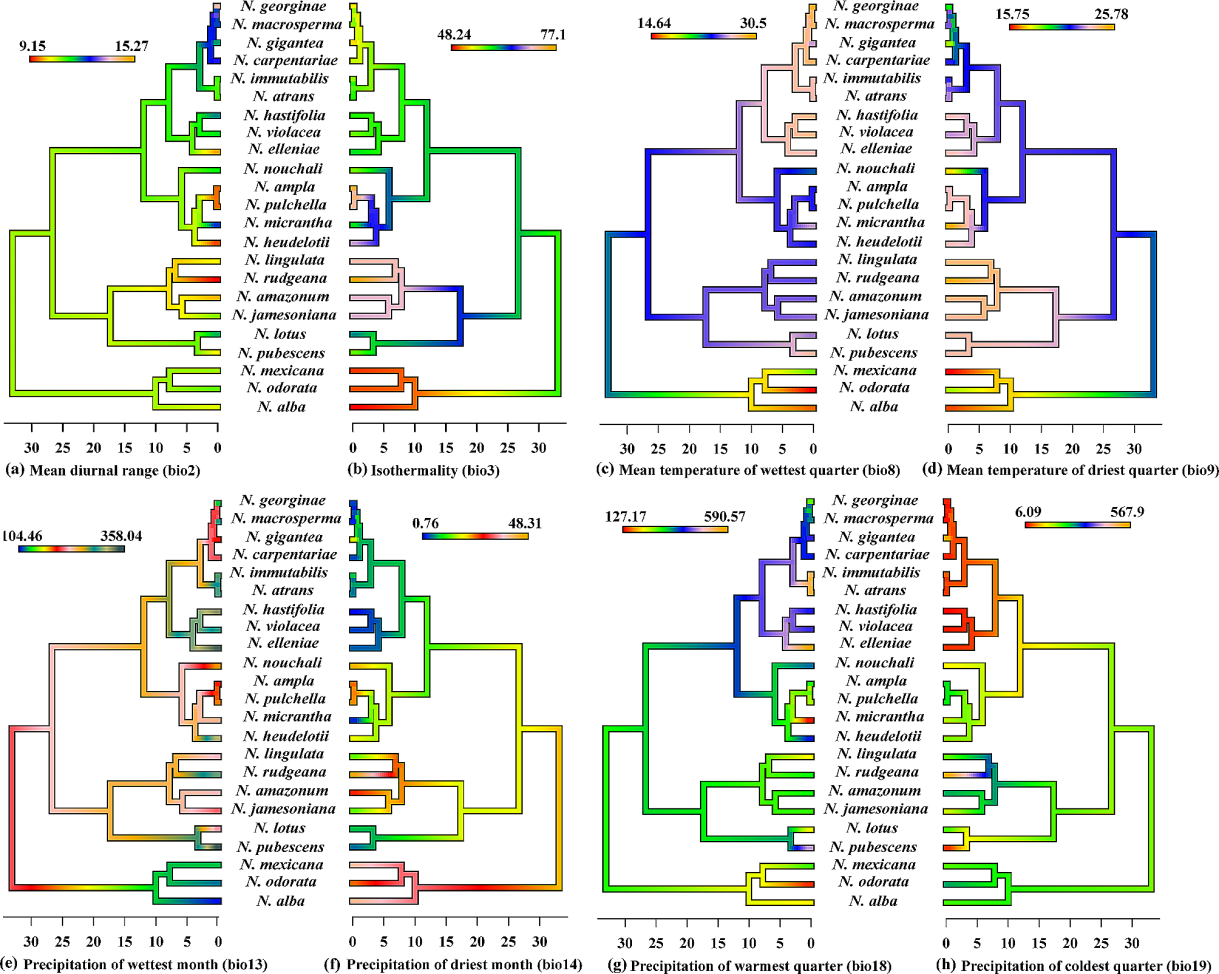
Reconstructed ancestral climatic preferences for *Nymphaea* species in eight climatic variables

The ML climatic ancestral reconstruction, based on the BM (Fig. 7), revealed both niche convergence and divergence in the analyzed bioclimatic variables. According to the evolutionary chronogram, *N. immutabilis*, *N. atrans*, *N. violancea*, and *N. nouchali* evolved to intermediate temperatures for bio2 and bio3 and toward extreme temperatures for bio8 and bio9. In contrast, the clade comprising *N. alba*, *N. odorata*, and *N. mexicana* evolved toward intermediate temperatures for bio2 but to extremely low temperatures for bio3 (colder conditions). Similarly, the clade consisting of *N. lingulata*, *N. rudgeana*, and *N. amazonum* showed distinct evolutionary patterns in response to the bioclimatic variables bio2, bio3, bio8, and bio9. The precipitation variables also displayed considerable variation among species. For example, *N. hastifolia*, *N. violancea*, and *N. elleniae* evolved toward both ends of climatic extremes, whereas *N. ampla* and *N. pulchella* tended to adapt to similar climatic conditions for all precipitation variables.

## Discussion

The combination of phylogenetic data and species niche distribution models has become increasingly popular in uncovering the intricate biogeographical histories, evolutionary changes in environmental niches, and potential underlying speciation processes of species in their habitat environments. The phylogenetic structure was aligned with that of Borsch et al. [33], and the chronology of cladogenesis, based on mutation priors, was consistent with that of both Borch et al. [33] and Lohne et al. [15]. Major diversification events for *Nymphaea* species are estimated to have occurred during the late Paleogene (Oligocene) and early Neogene (Miocene). In contrast, multiple radiations have occurred more recently since the mid-Miocene [15], which may be attributed to lower levels of divergence among clades and species, as demonstrated by the phylogenetic representation of ancestral tolerance profiles (ATPs). Additionally, the characteristic geographical distribution of these species also plays a role due to the limited inherent climatic diversity, implying potential niche conservatism within a short period [34], which suggested that these species underwent radiation due to climate change, converging on bioclimatic aspects that favored their habitat suitability. The radiation can also be referred to as gradual due to its restricted geographical distribution, which can be attributed to spatial and temporal factors that contribute to the observed patterns.

The distribution of *Nymphaea* species was mostly influenced by two bioclimatic factors bio2 (Mean diurnal temperature) and bio13 (Precipitation of wettest month). Gallou et al. [35] reported diurnal temperature to be widely correlated with the size of vascular plants. Likewise, precipitation may be related to reproductive aspects of Nymphaeaceae, as it has been reported that there is a correlation between flowering and seasonal flooding cycles in aquatic plants [36]. Studies have also shown that different fish and snake species exhibit both divergent and convergent patterns of evolution in response to climate change [4, 31]. Species that show convergent evolution under climate change conditions are likely to exhibit niche overlap, indicating their tolerance to common bioclimatic conditions. For example, in South America, the distribution of *Nymphaea* species is favored by the bioclimatic variables bio2 and bio13. Also, divergent evolution is observed due to differences in tolerance to bioclimatic variables in different geographical regions. For instance, *N. heudelotii* and the *N. alba* clade exhibit different responses to bio18. Although closely related species share similar ranges and clusters in PCA, indicating the influence of phylogenetic structuring on their ecological niche, their adaptation to bioclimatic conditions is limited. Notably, the first clade of the *N.* subg. *Nymphaea* provides clear evidence of this phenomenon.

The use of PNO analysis to study the impact of climate change on *Nymphaea* species provides valuable insights into the timing of speciation events and the distribution patterns of ecologically diverse species across different regions. The chronological representation of these species reveals why some areas have more variation and diversity of *Nymphaea* species than others. For instance, the distribution of *N.* subg. *Anecphya* in Australia shows shared ecological preferences for bioclimatic variables such as bio3, bio8, and bio19. Some of the tested variables show PNC for some clades and divergence in others. For example, in the case of *N.* subg. *Nymphaea* (*N. alba* + *N. odorata* + *N. mexicana*), we have different evidence of PNC and divergence. *N. alba* shows the same requirements for bio2, bio 8, bio 9, and bio13 as that of *N. odorata*, and *N. mexicana*. However, it occupies geographically different regions separated by highly varied habitats compared to the other two species, which is consistent with PNC leading to allopatric speciation through ecological selection [37]. However, *N.* subg. *Nymphaea* also shows niche divergence from other species in the group. On the other hand, we observed PNC for bio2, bio14, bio18, and bio19. Although most bioclimatic variables indicate a lack of PNC in ecological niche reconstruction models, niche divergence is evident across many of these variables. “Phylogenetic signal tests show that there is no phylogenetic niche conservatism (PNC) in most of the variables used. The absence could be attributed to climatic and geographical isolation during the species speciation phase [38]. “Similarly, Kozak and Wiens [39] demonstrated that high levels of niche evolution (divergence) are associated with low levels of climatic overlap among clades within a lineage. This high percentage of species with little overlap between clades would be the main cause of having values indicating the non-detection of PNC.” However, this result should be taken with caution since assuming that different PS values are sufficient to demonstrate PNC [6] is a mistake, as this assumption is only true when the underlying evolutionary model is BM [38]. It is vital to interpret PS as a measure of a pattern rather than conclusive evidence of high or low evolutionary rates because its complexity intertwines with various evolutionary processes [38]. However, other tests that we will discuss below would support and explain the absence of PNC in most of the bioclimatic variables. A cautionary note is necessary when interpreting this type of results because some authors argue that PNC is a mechanistic evolutionary process that can lead, under different circumstances and times, to patterns with conserved niches, constraints (divergent within limited available niches), or divergence [6, 12].”

Additionally, the variation in bioclimatic variables revealed the heterogeneity in ecological preferences among species. Sister species that have adapted to different environments tend to have limited dispersal and gene flow, ultimately resulting in niche divergence and allopatric speciation [40]. The extent of niche overlap in geographical space plays a crucial role in determining the degree of interaction or shared geographical aspects between species. Sympatric distribution occurs when the overlap thresholds of 0.5 for both the *D* and *I* dimensions were met [41] The *Hydrocalis* clade, mainly consisting of South American species, shows a high degree of niche overlap, while most other species display low niche overlap, indicating the absence of phylogenetic conservatism. Similar patterns of high niche diversification and low niche overlap have been observed in fish studies [42, 43]. The PNO profiles further illustrated that the species adapt to their ecological requirements. The different picks and profile breaths indicate the radiation of the species to a broader spectrum of requirements in ecological space. Although some overlapping picks showed species with similar ecological tolerances, most picks differ between species in terms of bioclimatic variables, except for variables such as bio19, which explains tolerance to similar climatic factors. This variable can be linked to the range expansion limit, as it reflects extreme conditions that could expose the species to frost and freezing.

The BM analysis suggested that values close to one indicate that character evolution aligns with a BM model. A value greater than 1.0 implies that closely related lineages are more similar than expected based on a BM model. Meanwhile, a value less than 1.0 indicates overdispersion, which means closely related lineages are more dissimilar from each other than predicted under a BM model. Although initially used to assess phenotypic trait evolution, in this study, DTT was used to assume that bioclimatic variables represent species-specific traits or physiological adaptations to climatic niches [44]. The DDT plots remain stable during the period of common ancestry until the late Neogene, when they become more irregular. The MDI values show positivity, indicating that disparity is mainly observed within subclades rather than among them, deviating from the null model predicted by BM of evolution [45]. While most bioclimatic variables in the DDT analysis exhibit disparities within the 95% confidence interval in support of the BM, the absence of niche conservatism could be attributed to climatic and geographical isolation during the species speciation phase [42].

In the analysis of the ancestral climate, it was found that the geographic area can have a significant impact on the evolution of a species. The habitat plays a crucial role in determining the response and variation of the species. For instance, two species, *N. hastifolia* and *N. violancea*, sharing the exact geographic location, exhibit similar responses to bio2 and bio3 variables. For instance, two species, *N. lotus* and *N. pubescens*, being geographically distant, show different responses, which highlights the influence of evolutionary history on the species’ adaptive environment. Interestingly, even the clades that are distantly related have evolved to adapt to similar climatic conditions. *N. ampla*, *N. pulchella*, and *N. mexicana* exhibited similar responses to bio18 and bio19, while *N. ampla* and *N. pulchella* exhibited similar responses to bio13 and bio14.

## Materials and methods

### Species occurrence

Species occurrence data were compiled from various sources, including the Global Biodiversity Information Facility (GBIF) and published studies [28–30, 46]. To ensure data quality, only records with more than five occurrences were included in the analysis. Data cleaning and spatial filtering were conducted using ArcGIS v10.8, and a thinning algorithm implemented in the spThin v.1.0.0 R package [47] was applied to reduce spatial autocorrelation by thinning occurrence points to a minimum distance of 5 km between each other. The resulting dataset was used for the subsequent analysis (Table S2).

### Climatic variable data

The current bioclimatic raster variables (bio1–19; Table S3) were obtained from the WorldClim database v1.4 [48] at a spatial resolution of 2.5 min. These variables were then clipped to the accessible area (M) for each species ranges based on freshwater ecoregions [49] to ensure consistency across all species ranges. To address multicollinearity issues, we extracted the values corresponding to the species occurrence points using the “extract” function in the raster package v3.5.0 (https://cran.r-project.org/web/packages/raster/). Subsequently, a multicollinearity test was conducted using the VIF function in the “*usdm*” v1.1-18 R package [50, 51], and eight variables were selected as the most significant predictors with the VIF values ranging between 1.578 and 3.595 (Table 6), which also aligned with the findings of Nzei et al. [28–30] in terms of their contribution to habitat distribution modeling.

**Table 6:** The reserved bioclimatic variable after variance inflation factors (VIF) correlation analysis

Bioclimatic variables	Code	VIF values
Mean Diurnal Range (°C)	bio2	2.206
Isothermality (°C×100)	bio3	1.578
Mean Temperature of Wettest Quarter (°C)	bio8	2.132
Mean Temperature of Driest Quarter (°C)	bio9	3.595
Precipitation of Wettest Month (mm)	bio13	2.343
Precipitation of Driest Month (mm)	bio14	2.425
Precipitation of Warmest Quarter (mm)	bio18	2.628
Precipitation of Coldest Quarter (mm)	bio19	1.910

### Assessment of realized niche

The realized niche of the *Nymphaea* species was analyzed based on the species climatic niche using point sampling for each occurrence point. Moreover, principal component analysis (PCA) was subsequently conducted in R v.4.0.4. Additionally, an analysis of variance (ANOVA) was performed to assess the realized niche variance for each bioclimatic variable among the species, followed by a post hoc test using Tukey’s honest significance differences (Tukey HSD), following Gaynor et al. [52] approach, to determine the significance of the distribution of climatic factors for the *Nymphaea* species.

### Ecological niche modeling

The potential distribution of each species was determined using the Bioclim algorithm implemented in the ‘dismo’ R package v1.3-8 [51]. The selection of the model criterion was made without considering the interconnections between variables or their explanatory power [53, 54]. This approach aligns with Hutchinson’s concept that the environmental niche of species encompasses all the conditions necessary for its persistence [55]. The model’s performance was evaluated using the area under the curve (AUC) and biserial point correlation (COR) metrics implemented in the ‘dismo’ R package v1.3-8 [51], following the methodology outlined by Engler et al. [53]. The threshold for determining suitable and unsuitable regions was set using the maximum specificity and sensitivity criterion (maxSS).

### Sequence data and processing

Sequence data comprising the ITS nuclear region and the noncoding *trn*T–*trn*F region were retrieved from the National Centre for Biotechnology Information using accession numbers obtained from published studies [19, 22, 24, 33] and cultivar and hybrid species were excluded from the analysis (Table S4). Priority was given to sequences that matched our study regions, and additional sequences from outside our jurisdiction were also included. Using Bayesian and maximum likelihood analyses, a dated phylogenetic tree was constructed for all *Nymphaea* species based on the ITS and *trn*T–*trn*F sequence data (Fig. S3). Two calibration points were used: 33.4 Ma for the root diversification time in the Paleocene period and 22.3 Ma for the most recent diversification time in the early Miocene [27] (more detailed methods in appendix 1). To ensure robustness in downstream analyses, lineages with fewer than five occurrences were excluded from the modeling of suitable geographical regions using the drop.tip function in the ape R package v5.6.2 [56]. This resulted in a refined and more reliable dated tree for subsequent phylogenetic analyses and the reconstruction of evolutionary rates.

### Comparison of species niche overlap

The climatic niche overlap of species in environmental space (E-space) was assessed using the PCA-env function in the ecospat v.3.5 R package [57]. This analysis involved calibrating the entire environmental space of the species and dividing it into a grid of 100 cells based on the unique environmental conditions of the study area. The kernel density function was then applied to smooth the density of species occurrences within each grid cell, thus mitigating bias. The niche overlap analysis utilized Schoenner’s *D* statistic, which measures the degree of overlap between species (with 1 indicating complete overlap and 0 indicating no overlap) and was calculated using the density grid cells of each species in the ecospat v.3.5 R package [57–60].

### Phylogenetic niche signals involved in niche evolutions

To analyze the climatic niche evolution of *Nymphaea* species, we constructed Predicted Niche Occupancy profiles (PNO) using the selected bioclimatic variables to have the most significant influence on the species niche [3, 61], and the ecological niche models of each species using the phyloclim R package v.0.9.5 [62] in accordance with the methods of Evans et al. [45]. The PNO profiles were then generated for each bioclimatic variable by binning them into 100 categories to obtain a histogram of suitability that represents the species tolerance for each bioclimatic variable. Weighted means and 1000 random values associated with the probability of distribution were extracted from each profile and utilized in subsequent niche evolution tests. Then, with the anc.clim function of the phytools v.2.0 R package [63], we reconstruct the ancestral climatic tolerances of the species for each climatic variable [32] using PNO profiles and the phylogenetic ultra-metric tree of the modeled species assuming Brownian-motion evolution (BM) for each node. Then, we analyzed (i) the ancestral state reconstruction of the species’ environmental niche evolution using the maximum clade credibility tree (MCCT) obtained from BEAST analysis [64], generalized least squares (GLS) estimates, and the PNO values for each species. Also, we explore the phylogenetic signal of each variable using two metrics of the phylosing function of the phytools package. The first was Blomberg’s *K* [12, 65], and the second metric was Pagel’s *λ* index [10]. This analysis was performed by randomly subsampling 1000 posterior trees from the Bayesian analysis and the 1000 sample values for each of the PNOs in the phytools [63].

In addition, we explore the mode of evolution of the bioclimatic variables using four evolutionary models fitted for each variable (niche component). They include Brownian motion (BM) [66], Ornstein-Uhlenbeck (OU) [67, 68], Pagel’s delta (δ), and early burst (EB) [69]. The models were evaluated using 1000 subsampled posterior trees using the Geiger R package v.2.0.10 [70]. Model selection was performed by comparing the log-likelihood values and Akaike information criterion (AIC) for small samples, with the best model chosen based on higher log-likelihood and lower AIC values [71]. The difference in AIC values (ΔAIC) was conducted to compare the best model with the remaining models, following the criteria outlined by Burnham and Anderson [72]. Models with ΔAIC < 2 were considered equivalent (denoted as “e”), those with ΔAIC ≥ 2 and ΔAIC < 7 were considered less or more distinct (*), those with ΔAIC ≥ 7 and ΔAIC < 10 were considered distinct (**), and those with ΔAIC ≥ 10 were considered different (***).

### Niche disparity distribution

The measurement of niche divergence and conservatism in niche evolution patterns in the BM model of trait evolution [73] utilized ecological disparity with time (DTT). It was done by calculating the average relative of all clades with ancestral lineages present at each speciation event. The analysis was conducted using the Geiger R package v.2.0.10 through 1000 simulations and a 95% confidence level [73]. The resulting disparity was then plotted against evolutionary time and quantified using the morphological disparity index (MDI) [32].

### Ancestral niche evolution

The impact of climate change on ancestral evolution was evaluated by analyzing a phylogenetic tree and utilizing values derived from bioclimatic variables within the accessible range of species. This assessment was performed using the contMap function of the phytools v.2.0 R package [63]. This function employs Felsenstein’s equation [66] to interpolate the inferred states of internal nodes in the evolutionary model to the branch edges.

## Conclusion

Through the integration of species distribution and phylo-climatic models, our study examined the effects of climate change on *Nymphaea* species across Africa, South America, and Australia. The findings suggested that climate change has impacted the habitat suitability and niche evolution of *Nymphaea* species, leading to changes in their populations. Additionally, the study revealed that each species responds differently to climatic variables, which allows for the exploration of novel distribution areas as climate change persists. This research deepens our understanding of how climatic niche changes can drive the evolution of *Nymphaea* and has important implications for conservation efforts and ecological resilience.

### Electronic supplementary material

Below is the link to the electronic supplementary material.

Supplementary Material 1

Supplementary Material 2

Supplementary Material 3

Supplementary Material 4

Supplementary Material 5

Supplementary Material 6

Supplementary Material 7

Supplementary Material 8

Supplementary Material 9

## Data Availability

The data presented in the study is available on the respective websites.

## Abbreviations

PNC: Phylogenetic niche conservatism
BM: Brownian motion
OU: Ornstein-Uhlenbeck
λ: Pagel’s
K: Blomberg’s
SDMs: Species distribution models
DTT: Disparity through time
GBIF: Global Biodiversity Information Facility
PCA: Principal component analysis
ANOVA: Analysis of variance
AUC: Under the curve
maxSS: Maximum specificity and sensitivity criterion
NCBI: National Centre for Biotechnology Information
PNO: Predicted Niche Occupancy
MCCT: Maximum clade credibility tree
GLS: Generalized least squares
EB: Early burst
AIC: Akaike information criterion
MDI: Morphological disparity index
