# Supplementary material for: Climatic niche evolution and niche conservatism of Nymphaea species in Africa, South America, and Australia
Source: BMC Plant Biol. 2024 May 30;24:476. doi: 10.1186/s12870-024-05141-1 (PMC11137912; doi:10.1186/s12870-024-05141-1)
Supplement: Supplementary file 1 — Supplementary Material 1 [file 12870_2024_5141_MOESM1_ESM.docx]

**Appendix 1** Detailed explanation of the Bayesian and dated tree reconstruction process

**Phylogenetic analysis**

All the sequences were individually aligned using MAFFT v.7.2.2 (Katoh and Standley 2013). The alignment was then trimmed using TrimAl v1.2 (Capella-Gutierrez et al. 2009) at default settings. The ambiguous alignments were cleaned using GBLOCKS (Talavera and Castresana 2007). The final clean alignment was assembled to a supermatrix and evaluated in Phylosuite v.1.2.1 (Zhang et al. 2020) for the phylogenetic analysis. *Barclaya longifolia* was selected as an outgroup as it represented the closest relative (Lohne et al. 2008). Using Modelfinder (Kalyaanamoorthy et al. 2017), TIM3+I+G was selected as the best model according to the corrected Akaike Information Criterion (AICc). MrBayes v.3.2.7 was used for the Bayesian Inference (BI) analysis (Ronquist et al. 2012) at a simulation run of 1000 generations for a total of 1 x 10^6^ generations of Markov Chain Monte Carlo (MCMC) and default chain heating temperature. The consensus tree was formed by the majority rule of the remaining trees (> 50 %) after discarding 25 % of burn-in, then the fixed standards were met at an average standard deviation (sd) of split frequencies of less than 0.003. Tracer v.1.7.2 was then used to visualize the posterior distribution of parameters (Rambaut et al. 2018). The final phylogenetic analysis was visualized in FigTree v1.4.4 (Rambaut 2018).

**Divergence time estimates**

As inferred from BI analysis, the divergence time was estimated in Beast v.1.8.4 (Drummond et al. 2012) using the generated supermatrix and *B. longifolia* as the outgroup species. The relaxed clock and Uncorrelated Lognormal (UCLN) model were used to avoid the challenges of substitution rates heterogeneity and possible ambiguity from the fossil data (Smith et al. 2010, Drummond et al. 2006). The model TIM3+I+G was adopted with six Gamma categories. The initial tree was randomly generated using the Yule process at 9 x 10^7^ MCMC generation in every 1000 sampled generations and discarding the first 10 % of generations. Lognormal distribution was selected for each calibration point for minimum nodes and maximum ones without hard limits (Popp et al. 2011; Adamson et al. 2010). Two calibration points were used for this analysis, the first was the Paleocene period (33.4 Ma) for the root diversification time and the second was the early Miocene (22.3 Ma) for the most recent diversification time (Lohne 2006) at 33.4 offset, mean of 1.0, and standard deviation of 1.0, respectively. TreeAnnotator v.1.8.4 (Drummond et al. 2012) was then used to calculate maximum clade credibility (MCC).

**References**

Adamson EAS, Hurwood DA, Mather PB (2010) A reappraisal of the evolution of Asian snakehead fishes (Pisces, Channidae) using molecular data from multiple genes and fossil calibration. Mol. Phylogenet. Evol. 56:707-717.

Capella-Gutiérrez S, Silla-Martínez JM, Gabaldón T (2009) trimAl: a tool for automated alignment trimming in large-scale phylogenetic analyses. Bioinformatics 25(15): 1972-1973.

Drummond AJ, Ho SY, Phillips MJ, Rambaut A (2006) Relaxed phylogenetics and dating with confidence. PLoS Biol. 4(5):e88.

Drummond MA, Suchard D, Xie A (2012) Rambaut Bayesian phylogenetics with BEAUti and the BEAST 1.7 Mol. Biol. Evol. 29 (8): 1969-1973.

Kalyaanamoorthy S, Minh BQ, Wong TKF, et al. (2017) ModelFinder: fast model selection for accurate phylogenetic estimates. Nat. Methods 14, 587-589.

Katoh K, Standley DM (2013) MAFFT multiple sequence alignment software version 7: improvements in performance and usability. Mol. Biol. and Evol. 30(4): 772-780.

Lohne C (2006) Molecular phylogenetics and historical biogeography of basal angiosperms: A case study in Nymphaeales. Doctoral dissertation, Bonn University.

Löhne C, Yoo MJ, Borsch T, et al (2008) Biogeography of Nymphaeales: Extant patterns and historical events. Taxon 57(4): 1123-1146.

Popp M, Mirre V, Brochmann C (2011) A single Mid-Pleistocene long-distance dispersal by a bird can explain the extreme bipolar disjunction in crowberries (*Empetrum*). Proc. Natl. Acad. Sci. USA 108:6520-6525.

Rambaut A (2018) FigTree v1. 4.4. http://tree.bio.ed.ac.uk/software/figtree/

Rambaut A, Drummond AJ, Xie D, et al. (2018) Posterior summarization in Bayesian phylogenetics using Tracer 1.7. Syst. Biol. 67(5): 901.

Ronquist F, Teslenko M, Van Der Mark P, et al. (2012) MrBayes 3.2: efficient Bayesian phylogenetic inference and model choice across a large model space. Syst. Biol. 61(3): 539-542.

Smith SA, Beaulieu JM, Donoghue MJ (2010) An uncorrelated relaxed-clock analysis suggests an earlier origin for flowering plants. Proc. Natl. Acad. Sci. USA, 107:5897-5902.

Talavera G, Castresana J (2007) Improvement of phylogenies after removing divergent and ambiguously aligned blocks from protein sequence alignments. Syst. Biol. 56(4): 564-577.

Zhang D, Gao F, Jakovlić I, et al. (2020) PhyloSuite: an integrated and scalable desktop platform for streamlined molecular sequence data management and evolutionary phylogenetic studies. Mol. Ecol. Resour. 20(1): 348-355.
