# Supplementary material for: Climatic niche evolution and niche conservatism of Nymphaea species in Africa, South America, and Australia
Source: BMC Plant Biol. 2024 May 30;24:476. doi: 10.1186/s12870-024-05141-1 (PMC11137912; doi:10.1186/s12870-024-05141-1)
Supplement: Supplementary file 2 — Supplementary Material 2 [file 12870_2024_5141_MOESM2_ESM.docx]

**Supplemental Figure Captions:**

**Fig. S1.** Divergence time of the 26 *Nymphaea* species in this study.

**Fig. S2.** Distribution of *Nymphaea*’s suitable habitat niches.

**Fig. S3.** *Nymphaea* and one outgroup species phylogenetic tree. The node numbers indicate Bayesian posterior probabilities (> 0.65) and maximum bootstrap values (> 57)
