# Supplementary material for: Climatic niche evolution and niche conservatism of Nymphaea species in Africa, South America, and Australia
Source: BMC Plant Biol. 2024 May 30;24:476. doi: 10.1186/s12870-024-05141-1 (PMC11137912; doi:10.1186/s12870-024-05141-1)
Supplement: Supplementary file 3 — Supplementary Material 3 [file 12870_2024_5141_MOESM3_ESM.docx]

**Table S1.** Schoener’s D (above diagonal) and Warren’s I (below horizontal) pairwise ecological niche overlap values (> 0.5= more overlap)

| **D/I** | **alb** | **odo** | **mex** | **pub** | **lot** | **jam** | **ama** | **rud** | **lin** | **heu** | **mic** | **pul** | **amp** | **nou** | **ell** | **vio** | **has** | **atr** | **imm** | **car** | **gig** | **mac** | **geo** |
| --- | --- | --- | --- | --- | --- | --- | --- | --- | --- | --- | --- | --- | --- | --- | --- | --- | --- | --- | --- | --- | --- | --- | --- |
| **alb** | NA | 0.18 | 0.601 | 0 | 0.001 | 0 | 0.008 | 0 | 0 | 0 | 0 | 0 | 0 | 0.033 | 0 | 0.003 | 0 | 0 | 0.005 | 0 | 0.08 | 0 | 0 |
| **odo** | 0.308 | NA | 0.175 | 0 | 0 | 0 | 0 | 0 | 0 | 0 | 0 | 0 | 0 | 0.004 | 0 | 0 | 0 | 0 | 0 | 0 | 0.002 | 0 | 0 |
| **mex** | 0.793 | 0.364 | NA | 0 | 0.001 | 0 | 0.006 | 0 | 0 | 0 | 0 | 0 | 0 | 0.038 | 0 | 0.003 | 0 | 0 | 0.004 | 0 | 0.083 | 0 | 0 |
| **pub** | 0 | 0 | 0 | NA | 0.007 | 0 | 0.001 | 0.001 | 0.001 | 0.004 | 0.002 | 0.002 | 0 | 0.004 | 0.375 | 0.096 | 0.124 | 0.238 | 0.065 | 0.009 | 0.017 | 0.115 | 0 |
| **lot** | 0.007 | 0.004 | 0.008 | 0.075 | NA | 0.326 | 0.331 | 0.34 | 0.337 | 0.251 | 0.491 | 0.45 | 0.45 | 0.524 | 0.014 | 0.157 | 0.016 | 0.003 | 0.136 | 0.039 | 0.144 | 0.027 | 0.001 |
| **jam** | 0 | 0 | 0 | 0 | 0.544 | NA | 0.441 | 0.295 | 0.59 | 0.499 | 0.159 | 0.528 | 0.475 | 0.257 | 0.001 | 0.026 | 0 | 0 | 0.009 | 0 | 0 | 0.001 | 0 |
| **ama** | 0.032 | 0 | 0.035 | 0.009 | 0.56 | 0.593 | NA | 0.538 | 0.572 | 0.418 | 0.14 | 0.593 | 0.568 | 0.258 | 0 | 0.05 | 0 | 0 | 0.055 | 0.001 | 0.047 | 0 | 0 |
| **rud** | 0.003 | 0 | 0.002 | 0.015 | 0.539 | 0.5 | 0.808 | NA | 0.407 | 0.308 | 0.138 | 0.528 | 0.561 | 0.232 | 0.001 | 0.036 | 0 | 0 | 0.028 | 0.003 | 0.021 | 0.001 | 0 |
| **lin** | 0 | 0 | 0 | 0.006 | 0.561 | 0.742 | 0.747 | 0.635 | NA | 0.449 | 0.172 | 0.584 | 0.5 | 0.238 | 0.001 | 0.027 | 0 | 0 | 0.017 | 0.002 | 0.011 | 0 | 0 |
| **heu** | 0 | 0 | 0 | 0.023 | 0.466 | 0.669 | 0.609 | 0.51 | 0.597 | NA | 0.111 | 0.455 | 0.431 | 0.22 | 0.003 | 0.048 | 0 | 0 | 0.044 | 0.001 | 0.03 | 0 | 0 |
| **mic** | 0 | 0 | 0 | 0.024 | 0.73 | 0.275 | 0.221 | 0.207 | 0.291 | 0.195 | NA | 0.202 | 0.19 | 0.191 | 0.005 | 0.08 | 0.013 | 0 | 0.06 | 0.03 | 0.058 | 0.028 | 0 |
| **pul** | 0 | 0 | 0 | 0.021 | 0.677 | 0.734 | 0.799 | 0.793 | 0.787 | 0.66 | 0.322 | NA | 0.738 | 0.352 | 0.002 | 0.038 | 0 | 0 | 0.023 | 0.002 | 0.015 | 0.001 | 0 |
| **amp** | 0 | 0 | 0 | 0.001 | 0.647 | 0.674 | 0.784 | 0.788 | 0.701 | 0.632 | 0.277 | 0.916 | NA | 0.347 | 0.001 | 0.023 | 0 | 0 | 0.007 | 0 | 0 | 0 | 0 |
| **nou** | 0.133 | 0.058 | 0.152 | 0.058 | 0.789 | 0.464 | 0.475 | 0.401 | 0.421 | 0.43 | 0.386 | 0.555 | 0.545 | NA | 0.006 | 0.136 | 0.003 | 0.002 | 0.163 | 0.017 | 0.275 | 0.006 | 0 |
| **ell** | 0 | 0 | 0 | 0.519 | 0.111 | 0.007 | 0.004 | 0.018 | 0.007 | 0.018 | 0.044 | 0.026 | 0.009 | 0.072 | NA | 0.284 | 0.276 | 0.275 | 0.168 | 0.131 | 0.047 | 0.316 | 0 |
| **vio** | 0.014 | 0 | 0.015 | 0.291 | 0.344 | 0.049 | 0.084 | 0.069 | 0.05 | 0.077 | 0.211 | 0.073 | 0.037 | 0.281 | 0.474 | NA | 0.324 | 0.063 | 0.553 | 0.393 | 0.314 | 0.49 | 0 |
| **has** | 0 | 0 | 0 | 0.207 | 0.119 | 0 | 0 | 0.001 | 0 | 0 | 0.097 | 0.002 | 0 | 0.053 | 0.363 | 0.543 | NA | 0.081 | 0.121 | 0.131 | 0.045 | 0.572 | 0 |
| **atr** | 0 | 0 | 0 | 0.325 | 0.055 | 0.002 | 0.003 | 0.01 | 0.006 | 0 | 0.01 | 0.012 | 0.001 | 0.039 | 0.469 | 0.235 | 0.153 | NA | 0.055 | 0.034 | 0.012 | 0.062 | 0 |
| **imm** | 0.013 | 0 | 0.012 | 0.211 | 0.325 | 0.013 | 0.108 | 0.091 | 0.038 | 0.078 | 0.154 | 0.063 | 0.011 | 0.322 | 0.353 | 0.802 | 0.288 | 0.223 | NA | 0.422 | 0.469 | 0.219 | 0.003 |
| **car** | 0 | 0 | 0 | 0.026 | 0.173 | 0 | 0.006 | 0.013 | 0.006 | 0.003 | 0.13 | 0.01 | 0 | 0.097 | 0.215 | 0.601 | 0.241 | 0.125 | 0.628 | NA | 0.262 | 0.24 | 0.012 |
| **gig** | 0.153 | 0.016 | 0.179 | 0.115 | 0.294 | 0.001 | 0.097 | 0.062 | 0.017 | 0.041 | 0.115 | 0.03 | 0 | 0.478 | 0.18 | 0.568 | 0.187 | 0.098 | 0.694 | 0.491 | NA | 0.096 | 0.002 |
| **mac** | 0 | 0 | 0 | 0.241 | 0.151 | 0.002 | 0 | 0.003 | 0.001 | 0.001 | 0.135 | 0.003 | 0.002 | 0.066 | 0.455 | 0.703 | 0.759 | 0.193 | 0.408 | 0.382 | 0.261 | NA | 0 |
| **geo** | 0 | 0 | 0 | 0 | 0.026 | 0 | 0 | 0 | 0 | 0 | 0 | 0 | 0 | 0.012 | 0 | 0.002 | 0 | 0 | 0.031 | 0.06 | 0.034 | 0 | NA |

The abbreviations represent: alb = *N. alba*, odo = *N. odorta*, mex = *N. mexicana*, lot = *N. lotus*, jam = *N. jamesoniana*, ama = *N. amazonum*, rud = *N. rudgeana*, lin = *N. lingulata*, heu = *N. heudelotii*, mic = *N. micrantha*, pul = *N. pulchella*, amp = *N. ampla*, nou = *N. nouchali*, ell = *N. elleniae*, vio = *N. violancea*, has = *N. hastifolia*, atr = *N. atrans*, imm = *N. immutabilis*, car = *N. carpentariae*, gig = *N. gigantea*, mac = *N. macrosperma*, geo = *N. georginae*
