# Supplementary material for: Climatic niche evolution and niche conservatism of Nymphaea species in Africa, South America, and Australia
Source: BMC Plant Biol. 2024 May 30;24:476. doi: 10.1186/s12870-024-05141-1 (PMC11137912; doi:10.1186/s12870-024-05141-1)
Supplement: Supplementary file 4 — Supplementary Material 4 [file 12870_2024_5141_MOESM4_ESM.docx]

**Table S2.** Spatial occurrence points for the species studied. (Log= Longitude, Lat= Latitude)

| **Species** | **Log** | **Lat** | **Species** | **Log** | **Lat** | **Species** | **Log** | **Lat** | **Species** | **Log** | **Lat** |
| --- | --- | --- | --- | --- | --- | --- | --- | --- | --- | --- | --- |
| *N. alba* | 144.314 | -38.19 | *N. alba* | 152.931 | -26.388 | *N. amazonum* | -39.507 | -3.494 | *N. amazonum* | -36.554 | -9.934 |
| *N. alba* | 152.469 | -31.94 | *N. alba* | 152.89 | -26.69 | *N. amazonum* | -39.491 | -7.079 | *N. amazonum* | -37.716 | -12.053 |
| *N. alba* | 151.004 | -33.98 | *N. alba* | 150.849 | -34.465 | *N. amazonum* | -39.478 | -3.8 | *N. amazonum* | -38.476 | -12.646 |
| *N. alba* | 145.367 | -38.06 | *N. alba* | 153.124 | -30.298 | *N. amazonum* | -39.433 | -3.738 | *N. amazonum* | -38.963 | -12.428 |
| *N. alba* | 145.205 | -37.81 | *N. alba* | 145.162 | -38.224 | *N. amazonum* | -39.411 | -6.403 | *N. amazonum* | -38.283 | -12.517 |
| *N. alba* | 145.143 | -37.84 | *N. alba* | 144.314 | -38.145 | *N. amazonum* | -39.383 | -6.245 | *N. amazonum* | -39.071 | -7.15 |
| *N. alba* | 147.5 | -41.93 | *N. alba* | 151.496 | -32.814 | *N. amazonum* | -39.359 | -5.558 | *N. amazonum* | -40.563 | -4.707 |
| *N. alba* | 144.559 | -37.41 | *N. alba* | 151.254 | -33.905 | *N. amazonum* | -39.298 | -3.586 | *N. amazonum* | -45.098 | -7.213 |
| *N. alba* | 148.212 | -41.32 | *N. alba* | 151.147 | -33.886 | *N. amazonum* | -39.176 | -6.405 | *N. amazonum* | -38.968 | -3.607 |
| *N. alba* | 144.136 | -37.97 | *N. alba* | 138.325 | -35.587 | *N. amazonum* | -39.144 | -7.223 | *N. amazonum* | -40.35 | -3.686 |
| *N. alba* | 143.986 | -38.52 | *N. alba* | 147.245 | -43.026 | *N. amazonum* | -38.428 | -12.944 | *N. amazonum* | -47.468 | -7.338 |
| *N. alba* | 139.45 | -34.85 | *N. alba* | 139.201 | -34.832 | *N. amazonum* | -38.314 | -5.509 | *N. amazonum* | -56.383 | -19.483 |
| *N. alba* | 146.488 | -38.21 | *N. amazonum* | -77.963 | -2.835 | *N. amazonum* | -38.103 | -4.219 | *N. amazonum* | -57.653 | -19.009 |
| *N. alba* | 145.388 | -36.35 | *N. amazonum* | -75.85 | 9.154 | *N. amazonum* | -37.808 | -4.512 | *N. amazonum* | -54.754 | -18.489 |
| *N. alba* | 151.221 | -33.73 | *N. amazonum* | -75.733 | 9.217 | *N. amazonum* | -37.25 | -6.583 | *N. amazonum* | -53.642 | -22.947 |
| *N. alba* | 138.487 | -35.51 | *N. amazonum* | -70.25 | -12 | *N. amazonum* | -35.6 | -6.804 | *N. amazonum* | -57.602 | -19.005 |
| *N. alba* | 144.609 | -37.31 | *N. amazonum* | -69.083 | -12.583 | *N. amazonum* | -35.55 | -6.723 | *N. amazonum* | -56.364 | -20.243 |
| *N. alba* | 142.543 | -37.64 | *N. amazonum* | -66.07 | 10.408 | *N. amazonum* | -35.523 | -7.306 | *N. amazonum* | -57.883 | -21.699 |
| *N. alba* | 138.686 | -34.85 | *N. amazonum* | -62.067 | 9.05 | *N. amazonum* | -35.491 | -6.984 | *N. amazonum* | -55.283 | -1.933 |
| *N. alba* | 138.767 | -35.1 | *N. amazonum* | -62.019 | -16.128 | *N. amazonum* | -35.367 | -7.009 | *N. amazonum* | -49.673 | -3.766 |
| *N. alba* | 144.25 | -35.83 | *N. amazonum* | -61.141 | -14.7 | *N. amazonum* | -35.316 | -7.184 | *N. amazonum* | -35.084 | -8.109 |
| *N. alba* | 150.501 | -33.7 | *N. amazonum* | -61.039 | -14.769 | *N. amazonum* | -35.261 | -6.816 | *N. amazonum* | -42.169 | -4.828 |
| *N. alba* | 139.458 | -35.25 | *N. amazonum* | -60.934 | -13.584 | *N. amazonum* | -35.218 | -7.189 | *N. amazonum* | -42.767 | -6.806 |
| *N. alba* | 151.22 | -33.85 | *N. amazonum* | -58.56 | 7.21 | *N. amazonum* | -79.617 | -1.15 | *N. amazonum* | -42.156 | -6.811 |
| *N. alba* | 149.42 | -35.1 | *N. amazonum* | -58.183 | -27.991 | *N. amazonum* | -69.283 | -12.817 | *N. amazonum* | -42.802 | -5.089 |
| *N. alba* | 146.018 | -34.22 | *N. amazonum* | -58.067 | -28.683 | *N. amazonum* | -66.173 | -11.147 | *N. amazonum* | -42.01 | -22.87 |
| *N. alba* | 151.65 | -30.48 | *N. amazonum* | -58 | -29 | *N. amazonum* | -65.883 | 10.1 | *N. amazonum* | -41.324 | -21.754 |
| *N. alba* | 145.367 | -37.67 | *N. amazonum* | -57.7 | 6.033 | *N. amazonum* | -61.938 | -10.884 | *N. amazonum* | -61.078 | -13.504 |
| *N. alba* | 150.749 | -34.8 | *N. amazonum* | -57.696 | -18.904 | *N. amazonum* | -50.925 | -29.712 | *N. amazonum* | -37.193 | -10.492 |
| *N. alba* | 145.483 | -37.98 | *N. amazonum* | -57.53 | -17.87 | *N. amazonum* | -39.91 | -3.241 | *N. ampla* | -41.508 | -10.173 |
| *N. alba* | 151.483 | -33.24 | *N. amazonum* | -57.333 | -23.117 | *N. amazonum* | -58.16 | 6.66 | *N. ampla* | -80.367 | -0.617 |
| *N. alba* | 151.478 | -33.06 | *N. amazonum* | -57.039 | 5.948 | *N. amazonum* | -57.781 | -17.67 | *N. ampla* | -79.95 | -2.716 |
| *N. alba* | 151.701 | -32.88 | *N. amazonum* | -56.298 | -16.515 | *N. amazonum* | -56.917 | -17.25 | *N. ampla* | -79.883 | -2.233 |
| *N. alba* | 147.546 | -30.84 | *N. amazonum* | -50.206 | -29.859 | *N. amazonum* | -56.607 | -16.257 | *N. ampla* | -79.367 | -0.583 |
| *N. alba* | 147.527 | -30.77 | *N. amazonum* | -41.024 | -11.691 | *N. amazonum* | -56.4 | -4.195 | *N. ampla* | -78.283 | -9.483 |
| *N. alba* | 145.539 | -37.64 | *N. amazonum* | -40.377 | -3.645 | *N. amazonum* | -49.276 | -20.38 | *N. ampla* | -77.593 | 1.335 |
| *N. alba* | 144.792 | -37.7 | *N. amazonum* | -40.294 | -4.274 | *N. amazonum* | -68.748 | -11.016 | *N. ampla* | -75.859 | 8.873 |
| *N. alba* | 150.913 | -33.68 | *N. amazonum* | -39.646 | -3.472 | *N. amazonum* | -36.667 | -9.417 | *N. ampla* | -75.73 | 9.22 |
| *N. ampla* | -74.55 | 4.658 | *N. carpentariae* | 144.895 | -18.888 | *N. elleniae* | 142.418 | -11.082 | *N. gigantea* | 149.55 | -25.483 |
| *N. ampla* | -74.433 | 4.67 | *N. carpentariae* | 141.111 | -17.661 | *N. elleniae* | 142.418 | -10.915 | *N. gigantea* | 141.086 | -17.641 |
| *N. ampla* | -74.38 | 4.342 | *N. carpentariae* | 139.548 | -17.748 | *N. elleniae* | 142.368 | -10.915 | *N. gigantea* | 150.299 | -24.139 |
| *N. ampla* | -74.12 | 6.438 | *N. carpentariae* | 143.351 | -18.301 | *N. elleniae* | 141.909 | -13.74 | *N. gigantea* | 145.643 | -17.247 |
| *N. ampla* | -69.25 | 11.067 | *N. carpentariae* | 138.04 | -19.74 | *N. elleniae* | 142.134 | -11.219 | *N. gigantea* | 153.206 | -29.587 |
| *N. ampla* | -68.333 | 10.85 | *N. carpentariae* | 138.229 | -18.454 | *N. elleniae* | 142.467 | -11.15 | *N. gigantea* | 150.353 | -23.155 |
| *N. ampla* | -57.039 | 5.948 | *N. carpentariae* | 145.022 | -19.004 | *N. georginae* | 137.913 | -20.957 | *N. gigantea* | 152.811 | -31.436 |
| *N. ampla* | -45.308 | -12.76 | *N. carpentariae* | 141.313 | -17.115 | *N. georginae* | 135.945 | -18.641 | *N. gigantea* | 152.766 | -30.957 |
| *N. ampla* | -41.416 | -8.581 | *N. carpentariae* | 140.56 | -18.613 | *N. georginae* | 144.22 | -23.399 | *N. gigantea* | 152.48 | -31.854 |
| *N. ampla* | -41.371 | -11.41 | *N. carpentariae* | 142.619 | -18.271 | *N. georginae* | 138.04 | -19.74 | *N. gigantea* | 145.071 | -18.112 |
| *N. ampla* | -41.15 | -11.55 | *N. carpentariae* | 138.043 | -18.59 | *N. georginae* | 137.835 | -20.008 | *N. gigantea* | 145.575 | -20.133 |
| *N. ampla* | -41.024 | -11.69 | *N. carpentariae* | 141.82 | -16.53 | *N. georginae* | 137.414 | -20.12 | *N. gigantea* | 145.581 | -20.009 |
| *N. ampla* | -40.725 | -4.449 | *N. carpentariae* | 141.059 | -17.673 | *N. georginae* | 138.12 | -19.58 | *N. gigantea* | 145.62 | -19.951 |
| *N. ampla* | -40.34 | -5.387 | *N. carpentariae* | 144.326 | -18.157 | *N. georginae* | 137.068 | -18.882 | *N. gigantea* | 145.12 | -18.198 |
| *N. ampla* | -40.261 | -3.802 | *N. carpentariae* | 141.668 | -15.665 | *N. georginae* | 138.126 | -19.94 | *N. gigantea* | 150.622 | -23.471 |
| *N. ampla* | -40.18 | -6.668 | *N. carpentariae* | 142.768 | -18.049 | *N. georginae* | 137.085 | -19.797 | *N. gigantea* | 151.37 | -33.462 |
| *N. ampla* | -40.122 | -4.753 | *N. carpentariae* | 141.08 | -17.58 | *N. georginae* | 137.502 | -20.03 | *N. gigantea* | 152.922 | -30.663 |
| *N. ampla* | -40.053 | -9.252 | *N. carpentariae* | 140.25 | -18.62 | *N. georginae* | 137.502 | -20.364 | *N. gigantea* | 149.159 | -21.157 |
| *N. ampla* | -39.76 | -3.73 | *N. carpentariae* | 140.17 | -17.92 | *N. georginae* | 136.152 | -18.472 | *N. gigantea* | 148.106 | -19.945 |
| *N. ampla* | -39.722 | -14.17 | *N. carpentariae* | 139.585 | -17.915 | *N. gigantea* | 152.673 | -25.624 | *N. gigantea* | 126.175 | -17.549 |
| *N. ampla* | -39.517 | -12.77 | *N. carpentariae* | 144.251 | -18.748 | *N. gigantea* | 152.464 | -25.725 | *N. gigantea* | 146.859 | -21.664 |
| *N. ampla* | -38.477 | -12.65 | *N. carpentariae* | 139.75 | -17.961 | *N. gigantea* | 152.425 | -25.676 | *N. gigantea* | 153.091 | -29.767 |
| *N. ampla* | -34.949 | -8.008 | *N. carpentariae* | 144.261 | -23.381 | *N. gigantea* | 152.696 | -25.79 | *N. gigantea* | 146.449 | -19.091 |
| *N. atrans* | 143.704 | -14.27 | *N. carpentariae* | 141.128 | -17.852 | *N. gigantea* | 151.258 | -23.898 | *N. gigantea* | 150.959 | -28.466 |
| *N. atrans* | 143.676 | -14.45 | *N. carpentariae* | 143.313 | -18.535 | *N. gigantea* | 152.34 | -27.483 | *N. gigantea* | 152.072 | -32.758 |
| *N. atrans* | 142.346 | -15.99 | *N. carpentariae* | 141.633 | -15.45 | *N. gigantea* | 152.416 | -25.797 | *N. gigantea* | 138.04 | -19.74 |
| *N. atrans* | 142.023 | -15.38 | *N. elleniae* | 145.123 | -15.682 | *N. gigantea* | 152.425 | -25.985 | *N. gigantea* | 149.576 | -25.223 |
| *N. atrans* | 142.183 | -15.46 | *N. elleniae* | 145.071 | -15.674 | *N. gigantea* | 152.48 | -25.97 | *N. gigantea* | 144.261 | -23.381 |
| *N. atrans* | 143.718 | -15.08 | *N. elleniae* | 143.676 | -14.446 | *N. gigantea* | 144.057 | -14.709 | *N. gigantea* | 153.167 | -30.163 |
| *N. atrans* | 142.551 | -13.57 | *N. elleniae* | 143.704 | -14.272 | *N. gigantea* | 145.154 | -15.426 | *N. gigantea* | 138.229 | -18.454 |
| *N. atrans* | 144.085 | -18.37 | *N. elleniae* | 143.024 | -13.203 | *N. gigantea* | 143.572 | -14.987 | *N. gigantea* | 151.332 | -24.418 |
| *N. atrans* | 143.906 | -14.64 | *N. elleniae* | 143.664 | -14.226 | *N. gigantea* | 149.516 | -22.347 | *N. gigantea* | 150.186 | -26.669 |
| *N. atrans* | 144.142 | -14.71 | *N. elleniae* | 142.857 | -15.159 | *N. gigantea* | 152.446 | -25.633 | *N. gigantea* | 137.493 | -20.025 |
| *N. atrans* | 145.225 | -15.5 | *N. elleniae* | 141.826 | -12.159 | *N. gigantea* | 152.623 | -25.614 | *N. gigantea* | 146.142 | -18.758 |
| *N. atrans* | 144.55 | -14.47 | *N. elleniae* | 142.395 | -11.141 | *N. gigantea* | 131.709 | -12.576 | *N. gigantea* | 138.052 | -18.587 |
| *N. atrans* | 144.25 | -15.15 | *N. elleniae* | 143.405 | -16.529 | *N. gigantea* | 153.069 | -30.363 | *N. gigantea* | 145.075 | -19.894 |
| *N. atrans* | 144.209 | -14.69 | *N. elleniae* | 142.704 | -16.537 | *N. gigantea* | 151.253 | -24.226 | *N. gigantea* | 152.824 | -30.725 |
| *N. carpentariae* | 145.673 | -22.6 | *N. elleniae* | 138.923 | -18.051 | *N. gigantea* | 147.207 | -19.567 | *N. gigantea* | 146.77 | -19.233 |
| *N. carpentariae* | 144.208 | -14.99 | *N. elleniae* | 144.278 | -16.551 | *N. gigantea* | 143.333 | -25.174 | *N. gigantea* | 145.062 | -18.21 |
| *N. carpentariae* | 140.541 | -18.03 | *N. elleniae* | 141.73 | -15.62 | *N. gigantea* | 152.744 | -31.641 | *N. gigantea* | 141.313 | -17.115 |
| *N. carpentariae* | 144.142 | -17.03 | *N. elleniae* | 142.33 | -11.15 | *N. gigantea* | 142.576 | -14.778 | *N. gigantea* | 142.4 | -18.355 |
| *N. gigantea* | 140.56 | -18.61 | *N. gigantea* | 131.134 | -13.332 | *N. gigantea* | 149.909 | -27.173 | *N. hastifolia* | 130.918 | -12.415 |
| *N. gigantea* | 140.592 | -18.45 | *N. gigantea* | 145.133 | -15.1 | *N. gigantea* | 153.267 | -28.983 | *N. hastifolia* | 131.201 | -12.549 |
| *N. gigantea* | 143.352 | -18.3 | *N. gigantea* | 151.501 | -24.498 | *N. gigantea* | 153 | -29.75 | *N. hastifolia* | 131.128 | -12.388 |
| *N. gigantea* | 143.478 | -18.29 | *N. gigantea* | 150.584 | -23.582 | *N. gigantea* | 148.57 | -20.43 | *N. hastifolia* | 130.467 | -13.198 |
| *N. gigantea* | 146.236 | -22.07 | *N. gigantea* | 149.584 | -22.415 | *N. gigantea* | 152.676 | -27.823 | *N. hastifolia* | 131.086 | -12.514 |
| *N. gigantea* | 152.891 | -29.69 | *N. gigantea* | 152.834 | -29.665 | *N. gigantea* | 152.626 | -27.89 | *N. hastifolia* | 125.702 | -14.831 |
| *N. gigantea* | 146.632 | -19.18 | *N. gigantea* | 152.675 | -27.675 | *N. gigantea* | 152.567 | -26.95 | *N. hastifolia* | 132.919 | -12.664 |
| *N. gigantea* | 145.964 | -20.23 | *N. gigantea* | 153.2 | -29.517 | *N. gigantea* | 148.751 | -25.082 | *N. hastifolia* | 130.486 | -15.347 |
| *N. gigantea* | 153.054 | -29.67 | *N. gigantea* | 153.13 | -29.67 | *N. gigantea* | 127.844 | -15.607 | *N. heudelotii* | 10.422 | -3.015 |
| *N. gigantea* | 151.926 | -24.51 | *N. gigantea* | 153.15 | -29.72 | *N. gigantea* | 151.2 | -24 | *N. heudelotii* | 9.42 | -2.016 |
| *N. gigantea* | 149.823 | -27.23 | *N. gigantea* | 140.917 | -17.75 | *N. gigantea* | 152.759 | -27.94 | *N. heudelotii* | 10.45 | -0.833 |
| *N. gigantea* | 150.693 | -26.69 | *N. gigantea* | 153.402 | -27.134 | *N. gigantea* | 152.918 | -27.082 | *N. heudelotii* | 9.455 | -0.682 |
| *N. gigantea* | 150.107 | -26.99 | *N. gigantea* | 150.07 | -24.87 | *N. gigantea* | 152.967 | -27.05 | *N. heudelotii* | 17.717 | -0.5 |
| *N. gigantea* | 149.785 | -25.62 | *N. gigantea* | 149.959 | -27.19 | *N. gigantea* | 130.033 | -15.75 | *N. heudelotii* | 11.567 | -0.217 |
| *N. gigantea* | 150.803 | -28.59 | *N. gigantea* | 149.193 | -27.24 | *N. gigantea* | 133.05 | -12.31 | *N. heudelotii* | 11.617 | -0.117 |
| *N. gigantea* | 152.243 | -25.57 | *N. gigantea* | 134.251 | -19.265 | *N. gigantea* | 145.63 | -16.82 | *N. heudelotii* | 9.41 | 0.611 |
| *N. gigantea* | 149.67 | -27.2 | *N. gigantea* | 135.7 | -11.95 | *N. gigantea* | 146.786 | -19.291 | *N. heudelotii* | 11.447 | 1.758 |
| *N. gigantea* | 152.626 | -27.69 | *N. gigantea* | 127.2 | -15.283 | *N. gigantea* | 147.482 | -19.625 | *N. heudelotii* | 15.175 | 2.058 |
| *N. gigantea* | 149.751 | -27.08 | *N. gigantea* | 152.393 | -27.573 | *N. gigantea* | 150.968 | -24.037 | *N. heudelotii* | 11.15 | 2.933 |
| *N. gigantea* | 146.226 | -21.29 | *N. gigantea* | 131 | -13 | *N. gigantea* | 153.058 | -26.701 | *N. heudelotii* | -3.867 | 5.233 |
| *N. gigantea* | 151.053 | -25.71 | *N. gigantea* | 152.5 | -27.5 | *N. gigantea* | 152.223 | -32.608 | *N. heudelotii* | 3.783 | 8.4 |
| *N. gigantea* | 150.418 | -23.42 | *N. gigantea* | 152.75 | -31 | *N. gigantea* | 153.1 | -29.5 | *N. heudelotii* | 13.667 | 8.733 |
| *N. gigantea* | 146.18 | -18.72 | *N. gigantea* | 128 | -17.5 | *N. gigantea* | 152.684 | -27.771 | *N. heudelotii* | -12.232 | 11.301 |
| *N. gigantea* | 152.952 | -29.63 | *N. gigantea* | 146 | -19.5 | *N. gigantea* | 145.8 | -16.917 | *N. heudelotii* | -5.676 | 11.372 |
| *N. gigantea* | 153.026 | -29.61 | *N. gigantea* | 150.5 | -23 | *N. gigantea* | 152.7 | -27.6 | *N. heudelotii* | -14.995 | 11.698 |
| *N. gigantea* | 153.062 | -29.54 | *N. gigantea* | 128 | -17 | *N. hastifolia* | 131.169 | -12.587 | *N. heudelotii* | -14.173 | 11.732 |
| *N. gigantea* | 153.052 | -29.41 | *N. gigantea* | 130.5 | -17.5 | *N. hastifolia* | 127.243 | -14.011 | *N. heudelotii* | 30.407 | 0.409 |
| *N. gigantea* | 153.207 | -29.39 | *N. gigantea* | 149 | -24 | *N. hastifolia* | 131.09 | -12.448 | *N. heudelotii* | 30.277 | 0.475 |
| *N. gigantea* | 132.024 | -12.82 | *N. gigantea* | 142.5 | -19.5 | *N. hastifolia* | 134.46 | -14.704 | *N. heudelotii* | 30.289 | 0.486 |
| *N. gigantea* | 142.067 | -15.97 | *N. gigantea* | 134.5 | -13 | *N. hastifolia* | 132.519 | -13.961 | *N. heudelotii* | 31.852 | 0.184 |
| *N. gigantea* | 132.341 | -12.61 | *N. gigantea* | 147 | -22 | *N. hastifolia* | 130.727 | -13.265 | *N. immutabilis* | 145.101 | -17.665 |
| *N. gigantea* | 145.209 | -17.14 | *N. gigantea* | 143 | -16 | *N. hastifolia* | 132.857 | -12.661 | *N. immutabilis* | 142.599 | -18.342 |
| *N. gigantea* | 125.968 | -16.83 | *N. gigantea* | 135.833 | -16 | *N. hastifolia* | 129.195 | -15.502 | *N. immutabilis* | 144.995 | -15.615 |
| *N. gigantea* | 132.083 | -14.7 | *N. gigantea* | 126.5 | -17 | *N. hastifolia* | 125.271 | -16.563 | *N. immutabilis* | 143.557 | -14.09 |
| *N. gigantea* | 125.833 | -14.75 | *N. gigantea* | 141 | -19 | *N. hastifolia* | 125.5 | -16.75 | *N. immutabilis* | 143.566 | -15.12 |
| *N. gigantea* | 150.751 | -23.75 | *N. gigantea* | 150.5 | -27.5 | *N. hastifolia* | 135.201 | -12.632 | *N. immutabilis* | 142.862 | -15.676 |
| *N. gigantea* | 131.217 | -12.37 | *N. gigantea* | 150.317 | -28.483 | *N. hastifolia* | 130.185 | -13.149 | *N. immutabilis* | 143.832 | -14.471 |
| *N. gigantea* | 132.692 | -13.05 | *N. gigantea* | 145.167 | -15.333 | *N. hastifolia* | 130.967 | -13.433 | *N. immutabilis* | 142.977 | -18.359 |
| *N. gigantea* | 143.274 | -25 | *N. gigantea* | 146.168 | -18.649 | *N. hastifolia* | 126.633 | -14.3 | *N. immutabilis* | 145.643 | -17.247 |
| *N. gigantea* | 152.751 | -27.75 | *N. gigantea* | 147.333 | -19.567 | *N. hastifolia* | 132.851 | -12.432 | *N. immutabilis* | 144.437 | -15.469 |
| *N. gigantea* | 128.717 | -15.97 | *N. gigantea* | 145 | -15 | *N. hastifolia* | 125.817 | -14.781 | *N. immutabilis* | 145.981 | -17.417 |
| *N. immutabilis* | 150.587 | -23.11 | *N. immutabilis* | 144.558 | -14.475 | *N. immutabilis* | 145.418 | -16.748 | *N. lingulata* | -40.656 | -6.404 |
| *N. immutabilis* | 130.937 | -17.31 | *N. immutabilis* | 145.33 | -16.75 | *N. immutabilis* | 145.418 | -17.582 | *N. lingulata* | -40.477 | -6.463 |
| *N. immutabilis* | 144.214 | -14.99 | *N. immutabilis* | 145.5 | -17.02 | *N. immutabilis* | 131.667 | -12.483 | *N. lingulata* | -40 | -5 |
| *N. immutabilis* | 141.855 | -12.05 | *N. immutabilis* | 144.25 | -15.15 | *N. immutabilis* | 145.501 | -17.498 | *N. lingulata* | -39.483 | -7.116 |
| *N. immutabilis* | 145.12 | -18.2 | *N. immutabilis* | 144.17 | -14.85 | *N. immutabilis* | 147.35 | -19.55 | *N. lingulata* | -39.411 | -6.404 |
| *N. immutabilis* | 145.049 | -17.19 | *N. immutabilis* | 145.05 | -18.7 | *N. immutabilis* | 126.2 | -17.2 | *N. lingulata* | -39.359 | -5.558 |
| *N. immutabilis* | 145.09 | -19.97 | *N. immutabilis* | 145 | -15.75 | *N. immutabilis* | 134.269 | -20.797 | *N. lingulata* | -39.333 | -4.435 |
| *N. immutabilis* | 145.365 | -16.8 | *N. immutabilis* | 144.325 | -15.192 | *N. immutabilis* | 130.402 | -17.497 | *N. lingulata* | -39.33 | -8.307 |
| *N. immutabilis* | 145.154 | -15.43 | *N. immutabilis* | 145.426 | -17.123 | *N. immutabilis* | 145.25 | -15.467 | *N. lingulata* | -39.314 | -5.509 |
| *N. immutabilis* | 141.136 | -17.86 | *N. immutabilis* | 145.2 | -17.142 | *N. immutabilis* | 150.467 | -23.367 | *N. lingulata* | -39.294 | -6.44 |
| *N. immutabilis* | 139.548 | -17.75 | *N. immutabilis* | 145.25 | -15.75 | *N. jamesoniana* | -79.983 | -3.483 | *N. lingulata* | -39.293 | -6.501 |
| *N. immutabilis* | 141.162 | -17.53 | *N. immutabilis* | 126.3 | -17.2 | *N. jamesoniana* | -75 | 10.75 | *N. lingulata* | -39.218 | -6.501 |
| *N. immutabilis* | 140.554 | -18.02 | *N. immutabilis* | 150.67 | -23.33 | *N. jamesoniana* | -60.913 | -13.594 | *N. lingulata* | -39.201 | -6.443 |
| *N. immutabilis* | 134.716 | -14.74 | *N. immutabilis* | 151 | -23.83 | *N. jamesoniana* | -39.359 | -5.558 | *N. lingulata* | -39.166 | -6.402 |
| *N. immutabilis* | 134.461 | -14.71 | *N. immutabilis* | 147.5 | -19.75 | *N. jamesoniana* | -39.276 | -6.501 | *N. lingulata* | -38.317 | -12.683 |
| *N. immutabilis* | 145.022 | -19 | *N. immutabilis* | 150.67 | -23.17 | *N. jamesoniana* | -39.202 | -6.377 | *N. lingulata* | -38.316 | -6.83 |
| *N. immutabilis* | 144.142 | -14.71 | *N. immutabilis* | 147.25 | -20.25 | *N. jamesoniana* | -39.148 | -6.425 | *N. lingulata* | -38.283 | -12.517 |
| *N. immutabilis* | 142.708 | -13.55 | *N. immutabilis* | 145.918 | -19.748 | *N. jamesoniana* | -36.631 | -9.416 | *N. lingulata* | -38.131 | -7.15 |
| *N. immutabilis* | 145.333 | -16.69 | *N. immutabilis* | 144.317 | -18.15 | *N. jamesoniana* | -40.505 | -9.438 | *N. lingulata* | -36.272 | -7.391 |
| *N. immutabilis* | 146.75 | -19.25 | *N. immutabilis* | 141.6 | -15.72 | *N. jamesoniana* | -38.543 | -3.718 | *N. lingulata* | -41.309 | -12.763 |
| *N. immutabilis* | 142.701 | -13.45 | *N. immutabilis* | 141.667 | -15.667 | *N. jamesoniana* | -46.492 | -14.481 | *N. lingulata* | -40.124 | -6.574 |
| *N. immutabilis* | 145.129 | -17.7 | *N. immutabilis* | 141.8 | -15.2 | *N. jamesoniana* | -43.347 | -4.875 | *N. lingulata* | -38.964 | -6.753 |
| *N. immutabilis* | 143.906 | -14.64 | *N. immutabilis* | 142.776 | -18.057 | *N. jamesoniana* | -45.265 | -6.893 | *N. lingulata* | -38.682 | -3.891 |
| *N. immutabilis* | 143.731 | -14.71 | *N. immutabilis* | 145.967 | -17.333 | *N. jamesoniana* | -56.625 | -16.257 | *N. lingulata* | -38.946 | -8.8 |
| *N. immutabilis* | 146.038 | -17.57 | *N. immutabilis* | 141.084 | -17.582 | *N. jamesoniana* | -56.049 | -15.874 | *N. lingulata* | -40.563 | -4.707 |
| *N. immutabilis* | 145.062 | -18.21 | *N. immutabilis* | 130.717 | -13.733 | *N. jamesoniana* | -56.383 | -19.483 | *N. lingulata* | -50.571 | -14.745 |
| *N. immutabilis* | 142.619 | -18.27 | *N. immutabilis* | 150.25 | -22.75 | *N. jamesoniana* | -56.55 | -21.25 | *N. lingulata* | -49.668 | -16.596 |
| *N. immutabilis* | 145.58 | -19.97 | *N. immutabilis* | 143.359 | -18.29 | *N. jamesoniana* | -56.95 | -19.783 | *N. lingulata* | -47.061 | -15.043 |
| *N. immutabilis* | 129.039 | -15.97 | *N. immutabilis* | 145.25 | -16.667 | *N. jamesoniana* | -54.954 | -18.352 | *N. lingulata* | -45.098 | -7.214 |
| *N. immutabilis* | 146.836 | -19.76 | *N. immutabilis* | 145.167 | -17.917 | *N. jamesoniana* | -43.674 | -15.338 | *N. lingulata* | -43.703 | -6.487 |
| *N. immutabilis* | 145.123 | -15.09 | *N. immutabilis* | 147.18 | -19.57 | *N. jamesoniana* | -39.823 | -8.788 | *N. lingulata* | -57.65 | -19.017 |
| *N. immutabilis* | 145.227 | -15.51 | *N. immutabilis* | 146.183 | -18.5 | *N. jamesoniana* | -42.767 | -6.806 | *N. lingulata* | -56.383 | -19.483 |
| *N. immutabilis* | 144.058 | -14.75 | *N. immutabilis* | 134.251 | -19.265 | *N. jamesoniana* | -44.639 | -10.228 | *N. lingulata* | -49.464 | -18.965 |
| *N. immutabilis* | 143.839 | -14.67 | *N. immutabilis* | 132.9 | -12.183 | *N. lingulata* | -63.65 | -18.133 | *N. lingulata* | -44.898 | -17.336 |
| *N. immutabilis* | 146.727 | -19.36 | *N. immutabilis* | 140.183 | -17.933 | *N. lingulata* | -59.41 | 2.83 | *N. lingulata* | -54.096 | -2.462 |
| *N. immutabilis* | 138.043 | -18.59 | *N. immutabilis* | 138.567 | -17.9 | *N. lingulata* | -54.575 | -20.478 | *N. lingulata* | -38.228 | -6.759 |
| *N. immutabilis* | 135.936 | -18.64 | *N. immutabilis* | 138.25 | -17.27 | *N. lingulata* | -47.334 | -15.537 | *N. lingulata* | -37.28 | -7.024 |
| *N. immutabilis* | 128.3 | -15.6 | *N. immutabilis* | 145.176 | -15.09 | *N. lingulata* | -44.564 | -17.09 | *N. lingulata* | -41.661 | -22.187 |
| *N. immutabilis* | 146.217 | -21.28 | *N. immutabilis* | 143.75 | -15 | *N. lingulata* | -43.356 | -4.863 | *N. lingulata* | -37.193 | -10.492 |
| *N. immutabilis* | 141.817 | -16.53 | *N. immutabilis* | 134.22 | -20.8 | *N. lingulata* | -40.87 | -4.038 | *N. lotus* | 33.156 | 1.124 |
| *N. immutabilis* | 125.1 | -16.9 | *N. immutabilis* | 145.418 | -16.915 | *N. lingulata* | -40.725 | -4.449 | *N. lotus* | 10.518 | 11.255 |
| *N. lotus* | 32.495 | 0.089 | *N. lotus* | -16.213 | 11.092 | *N. lotus* | -7.983 | 12.633 | *N. lotus* | -3.433 | 12.833 |
| *N. lotus* | 11.168 | 10.282 | *N. lotus* | -16.117 | 16.067 | *N. lotus* | -7.85 | 11.4 | *N. lotus* | -3.417 | 13.167 |
| *N. lotus* | 6.656 | 12.119 | *N. lotus* | -16.083 | 16.3 | *N. lotus* | -7.783 | 12.667 | *N. lotus* | -3.167 | 5.55 |
| *N. lotus* | 30.576 | -0.14 | *N. lotus* | -15.986 | 16.29 | *N. lotus* | -7.217 | 12.017 | *N. lotus* | -3.083 | 14.65 |
| *N. lotus* | 3.861 | 7.423 | *N. lotus* | -15.983 | 15.933 | *N. lotus* | -6.8 | 7.767 | *N. lotus* | -3.05 | 9.3 |
| *N. lotus* | 10.228 | 12.648 | *N. lotus* | -15.917 | 16.183 | *N. lotus* | -6.333 | 7.867 | *N. lotus* | -2.94 | 15.006 |
| *N. lotus* | 33.202 | 0.485 | *N. lotus* | -15.733 | 16.383 | *N. lotus* | -6.267 | 13.45 | *N. lotus* | -2.933 | 11.75 |
| *N. lotus* | 8.9 | 9.857 | *N. lotus* | -15.25 | 12.85 | *N. lotus* | -6.05 | 7.433 | *N. lotus* | -2.7 | 10.833 |
| *N. lotus* | 7.312 | 11.716 | *N. lotus* | -15.083 | 11.777 | *N. lotus* | -5.983 | 14.217 | *N. lotus* | -2.42 | 10.5 |
| *N. lotus* | 7.723 | 12.692 | *N. lotus* | -14.952 | 13.554 | *N. lotus* | -5.901 | 14.532 | *N. lotus* | -2.273 | 13 |
| *N. lotus* | 7.769 | 13.172 | *N. lotus* | -14.952 | 12.03 | *N. lotus* | -5.633 | 9.55 | *N. lotus* | -2.101 | 11.493 |
| *N. lotus* | 4.227 | 12.479 | *N. lotus* | -14.901 | 11.136 | *N. lotus* | -5.517 | 9.9 | *N. lotus* | -2.1 | 11.25 |
| *N. lotus* | 7.395 | 11.099 | *N. lotus* | -14.9 | 12.833 | *N. lotus* | -5.45 | 9.35 | *N. lotus* | -1.95 | 11.333 |
| *N. lotus* | 38.635 | -4.609 | *N. lotus* | -14.574 | 12.395 | *N. lotus* | -5.367 | 8.033 | *N. lotus* | -1.861 | 9.259 |
| *N. lotus* | 32.876 | -1.433 | *N. lotus* | -14.533 | 14.233 | *N. lotus* | -5.35 | 13.967 | *N. lotus* | -1.817 | 12.2 |
| *N. lotus* | 13.361 | 12.253 | *N. lotus* | -14.197 | 11.021 | *N. lotus* | -5.333 | 9.433 | *N. lotus* | -1.7 | 14.167 |
| *N. lotus* | 38.573 | -4.294 | *N. lotus* | -14.048 | 11.843 | *N. lotus* | -5.3 | 7.85 | *N. lotus* | -1.495 | 12.388 |
| *N. lotus* | 30.277 | -0.475 | *N. lotus* | -13.4 | 16.183 | *N. lotus* | -5.183 | 9.583 | *N. lotus* | -1.3 | 12.033 |
| *N. lotus* | 37.532 | -6.663 | *N. lotus* | -13.1 | 9.417 | *N. lotus* | -5.033 | 7.683 | *N. lotus* | -1.25 | 14.767 |
| *N. lotus* | 34.917 | -7.65 | *N. lotus* | -13.016 | 13.3 | *N. lotus* | -5.017 | 14.133 | *N. lotus* | -1.125 | 9.125 |
| *N. lotus* | 6.138 | 9.053 | *N. lotus* | -12.999 | 11.624 | *N. lotus* | -4.9 | 13.3 | *N. lotus* | -1.05 | 14.017 |
| *N. lotus* | 34.837 | -0.154 | *N. lotus* | -12.383 | 17.967 | *N. lotus* | -4.836 | 10.646 | *N. lotus* | -0.467 | 14.667 |
| *N. lotus* | 37.032 | -6.544 | *N. lotus* | -12.272 | 17.904 | *N. lotus* | -4.6 | 15.617 | *N. lotus* | -0.412 | 14.343 |
| *N. lotus* | 30.218 | -12.84 | *N. lotus* | -12.25 | 12.533 | *N. lotus* | -4.45 | 6.983 | *N. lotus* | -0.35 | 16.95 |
| *N. lotus* | 34.717 | -0.101 | *N. lotus* | -12.117 | 13.067 | *N. lotus* | -4.372 | 11.375 | *N. lotus* | -0.333 | 14.617 |
| *N. lotus* | 11.048 | 12.257 | *N. lotus* | -11.548 | 15.882 | *N. lotus* | -4.25 | 11.167 | *N. lotus* | -0.283 | 14.35 |
| *N. lotus* | 11.066 | 12.848 | *N. lotus* | -11.533 | 16.017 | *N. lotus* | -4.217 | 5.8 | *N. lotus* | -0.233 | 5.6 |
| *N. lotus* | 6.425 | 12.244 | *N. lotus* | -11.409 | 16.549 | *N. lotus* | -4.217 | 14.517 | *N. lotus* | -0.133 | 12.133 |
| *N. lotus* | -16.763 | 13.088 | *N. lotus* | -11.352 | 6.874 | *N. lotus* | -4.183 | 5.95 | *N. lotus* | -0.05 | 16.283 |
| *N. lotus* | -16.7 | 15.117 | *N. lotus* | -10.733 | 17.35 | *N. lotus* | -4.183 | 15.367 | *N. lotus* | -0.033 | 14.05 |
| *N. lotus* | -16.656 | 13.203 | *N. lotus* | -10.433 | 6.467 | *N. lotus* | -4.167 | 5.35 | *N. lotus* | 0.067 | 5.767 |
| *N. lotus* | -16.467 | 16.033 | *N. lotus* | -10.367 | 16.033 | *N. lotus* | -4.15 | 11.75 | *N. lotus* | 0.07 | 7.53 |
| *N. lotus* | -16.45 | 14.367 | *N. lotus* | -10.15 | 16.917 | *N. lotus* | -4.083 | 8.017 | *N. lotus* | 0.117 | 7.883 |
| *N. lotus* | -16.433 | 13.667 | *N. lotus* | -10.15 | 17.083 | *N. lotus* | -4.05 | 5.383 | *N. lotus* | 0.35 | 14.05 |
| *N. lotus* | -16.417 | 12.467 | *N. lotus* | -9.667 | 15.75 | *N. lotus* | -3.983 | 5.267 | *N. lotus* | 0.9 | 14.2 |
| *N. lotus* | -16.383 | 16.233 | *N. lotus* | -9.483 | 15.7 | *N. lotus* | -3.883 | 5.4 | *N. lotus* | 0.98 | 10.708 |
| *N. lotus* | -16.367 | 16.617 | *N. lotus* | -9.45 | 15.6 | *N. lotus* | -3.817 | 8.733 | *N. lotus* | 1.021 | 10.409 |
| *N. lotus* | -16.3 | 16.417 | *N. lotus* | -9.267 | 15.5 | *N. lotus* | -3.5 | 6.733 | *N. lotus* | 1.033 | 14.183 |
| *N. lotus* | -16.261 | 16.205 | *N. lotus* | -8.77 | 5.418 | *N. lotus* | -3.483 | 14.233 | *N. lotus* | 1.038 | 11.018 |
| *N. lotus* | -16.217 | 12.983 | *N. lotus* | -8.272 | 7.394 | *N. lotus* | -3.467 | 6.3 | *N. lotus* | 1.1 | 6.3 |
| *N. lotus* | -16.215 | 16.539 | *N. lotus* | -8.05 | 5.417 | *N. lotus* | -3.45 | 12.967 | *N. lotus* | 1.2 | 6.2 |
| *N. lotus* | 1.209 | 10.542 | *N. lotus* | 2.4 | 12.267 | *N. lotus* | 5.533 | 13.917 | *N. lotus* | 10.817 | 3.867 |
| *N. lotus* | 1.229 | 10.847 | *N. lotus* | 2.417 | 12.467 | *N. lotus* | 5.683 | 6.15 | *N. lotus* | 10.933 | 8.733 |
| *N. lotus* | 1.272 | 10.707 | *N. lotus* | 2.421 | 8.496 | *N. lotus* | 5.7 | 15.383 | *N. lotus* | 11.428 | 3.88 |
| *N. lotus* | 1.35 | 10.883 | *N. lotus* | 2.423 | 7.173 | *N. lotus* | 5.8 | 6.283 | *N. lotus* | 11.5 | 3.483 |
| *N. lotus* | 1.355 | 10.27 | *N. lotus* | 2.433 | 11.967 | *N. lotus* | 6.033 | 6.35 | *N. lotus* | 11.58 | 3.63 |
| *N. lotus* | 1.383 | 10.75 | *N. lotus* | 2.473 | 6.719 | *N. lotus* | 6.767 | 17.1 | *N. lotus* | 11.668 | 10.066 |
| *N. lotus* | 1.422 | 11.148 | *N. lotus* | 2.5 | 6.45 | *N. lotus* | 7.63 | 11.182 | *N. lotus* | 12.1 | 13.133 |
| *N. lotus* | 1.45 | 14.217 | *N. lotus* | 2.513 | 6.622 | *N. lotus* | 7.716 | 11.051 | *N. lotus* | 12.2 | 2.1 |
| *N. lotus* | 1.483 | 10.9 | *N. lotus* | 2.55 | 12.517 | *N. lotus* | 7.98 | 7.454 | *N. lotus* | 12.25 | 3.75 |
| *N. lotus* | 1.483 | 11.033 | *N. lotus* | 2.567 | 9.417 | *N. lotus* | 8.55 | 7.767 | *N. lotus* | 12.25 | 13.133 |
| *N. lotus* | 1.49 | 11.446 | *N. lotus* | 2.6 | 9.3 | *N. lotus* | 8.684 | 11.858 | *N. lotus* | 12.4 | 7.483 |
| *N. lotus* | 1.553 | 11.289 | *N. lotus* | 2.616 | 6.383 | *N. lotus* | 8.867 | 11.9 | *N. lotus* | 12.611 | 0.301 |
| *N. lotus* | 1.598 | 6.863 | *N. lotus* | 2.667 | 6.533 | *N. lotus* | 9.167 | 13.8 | *N. lotus* | 12.8 | 0.5 |
| *N. lotus* | 1.65 | 6.25 | *N. lotus* | 2.716 | 9.871 | *N. lotus* | 9.2 | 4.17 | *N. lotus* | 12.933 | 0.817 |
| *N. lotus* | 1.667 | 6.767 | *N. lotus* | 2.729 | 6.676 | *N. lotus* | 9.338 | -0.377 | *N. lotus* | 13.183 | 1.167 |
| *N. lotus* | 1.667 | 6.367 | *N. lotus* | 2.75 | 11.017 | *N. lotus* | 9.433 | 0.433 | *N. lotus* | 13.25 | 10.333 |
| *N. lotus* | 1.695 | 6.676 | *N. lotus* | 2.8 | 12.417 | *N. lotus* | 9.497 | -0.618 | *N. lotus* | 13.4 | 9.267 |
| *N. lotus* | 1.696 | 6.897 | *N. lotus* | 2.917 | 12.417 | *N. lotus* | 9.538 | -2.043 | *N. lotus* | 13.459 | -8.693 |
| *N. lotus* | 1.717 | 13.683 | *N. lotus* | 2.933 | 11.9 | *N. lotus* | 9.567 | 14.033 | *N. lotus* | 14.167 | 13.433 |
| *N. lotus* | 1.739 | 6.478 | *N. lotus* | 3.102 | 11.121 | *N. lotus* | 9.6 | 0.867 | *N. lotus* | 14.233 | 10.3 |
| *N. lotus* | 1.811 | 6.287 | *N. lotus* | 3.117 | 11.65 | *N. lotus* | 9.617 | 1.35 | *N. lotus* | 14.283 | 13.433 |
| *N. lotus* | 1.817 | 13.667 | *N. lotus* | 3.239 | 11.513 | *N. lotus* | 9.65 | 4.967 | *N. lotus* | 14.3 | 13.567 |
| *N. lotus* | 1.928 | 13.599 | *N. lotus* | 3.243 | 11.134 | *N. lotus* | 9.683 | 4.017 | *N. lotus* | 14.333 | 10.35 |
| *N. lotus* | 2 | 12.317 | *N. lotus* | 3.27 | 11.395 | *N. lotus* | 9.729 | 1.778 | *N. lotus* | 14.667 | 12.867 |
| *N. lotus* | 2.017 | 7 | *N. lotus* | 3.317 | 11.632 | *N. lotus* | 9.783 | 4.45 | *N. lotus* | 14.717 | 12.3 |
| *N. lotus* | 2.033 | 13.383 | *N. lotus* | 3.358 | 11.058 | *N. lotus* | 9.8 | 4 | *N. lotus* | 14.8 | 10.383 |
| *N. lotus* | 2.1 | 13.517 | *N. lotus* | 3.383 | 11.867 | *N. lotus* | 9.85 | -1.9 | *N. lotus* | 14.867 | 12.933 |
| *N. lotus* | 2.126 | 6.742 | *N. lotus* | 3.417 | 6.917 | *N. lotus* | 9.9 | 2.867 | *N. lotus* | 14.933 | 12.667 |
| *N. lotus* | 2.145 | 6.86 | *N. lotus* | 3.433 | 12.567 | *N. lotus* | 9.967 | 4.467 | *N. lotus* | 15 | 12.083 |
| *N. lotus* | 2.148 | 7.226 | *N. lotus* | 3.536 | 6.442 | *N. lotus* | 10.033 | 12.435 | *N. lotus* | 15.1 | 12.067 |
| *N. lotus* | 2.161 | 9.338 | *N. lotus* | 4.083 | 15.083 | *N. lotus* | 10.033 | 2.4 | *N. lotus* | 15.2 | 10.483 |
| *N. lotus* | 2.165 | 9.55 | *N. lotus* | 4.517 | 10.35 | *N. lotus* | 10.083 | 0.25 | *N. lotus* | 15.217 | 11.8 |
| *N. lotus* | 2.167 | 12.417 | *N. lotus* | 4.65 | 10.083 | *N. lotus* | 10.117 | 2.817 | *N. lotus* | 15.25 | 9.967 |
| *N. lotus* | 2.183 | 6.971 | *N. lotus* | 4.667 | 10.633 | *N. lotus* | 10.162 | -0.848 | *N. lotus* | 15.25 | 10.117 |
| *N. lotus* | 2.264 | 8.994 | *N. lotus* | 4.75 | 13.833 | *N. lotus* | 10.417 | 1.542 | *N. lotus* | 15.35 | -4.467 |
| *N. lotus* | 2.267 | 12.167 | *N. lotus* | 4.833 | 13.983 | *N. lotus* | 10.417 | 3.083 | *N. lotus* | 15.483 | 10.517 |
| *N. lotus* | 2.283 | 12.417 | *N. lotus* | 5.167 | 14.367 | *N. lotus* | 10.45 | -2.233 | *N. lotus* | 15.6 | 12.833 |
| *N. lotus* | 2.285 | 7.188 | *N. lotus* | 5.267 | 14.283 | *N. lotus* | 10.462 | 12.868 | *N. lotus* | 15.833 | 12.833 |
| *N. lotus* | 2.355 | 6.696 | *N. lotus* | 5.274 | 13.225 | *N. lotus* | 10.499 | -0.814 | *N. lotus* | 15.967 | 11.617 |
| *N. lotus* | 2.367 | 13.117 | *N. lotus* | 5.417 | 14 | *N. lotus* | 10.5 | 6.333 | *N. lotus* | 16.15 | 2.367 |
| *N. lotus* | 2.397 | 6.575 | *N. lotus* | 5.467 | 6.333 | *N. lotus* | 10.5 | 9.75 | *N. lotus* | 16.233 | 12.467 |
| *N. lotus* | 16.933 | 13.283 | *N. lotus* | 30.394 | -12.621 | *N. lotus* | 37.833 | 6.583 | *N. macrosperma* | 131.707 | -12.6 |
| *N. lotus* | 17.467 | 12.883 | *N. lotus* | 30.745 | -30.293 | *N. lotus* | 39.76 | -3.824 | *N. macrosperma* | 129.884 | -13.985 |
| *N. lotus* | 17.667 | 8.683 | *N. lotus* | 31.067 | 9.483 | *N. lotus* | 39.794 | -3.373 | *N. macrosperma* | 132.735 | -11.815 |
| *N. lotus* | 18.65 | 8.683 | *N. lotus* | 31.125 | -27.875 | *N. lotus* | 40.383 | 5.617 | *N. macrosperma* | 132.834 | -12.495 |
| *N. lotus* | 19.625 | -17.88 | *N. lotus* | 31.125 | -26.125 | *N. lotus* | 43.637 | -21.996 | *N. macrosperma* | 132.518 | -12.849 |
| *N. lotus* | 19.833 | 10.217 | *N. lotus* | 31.125 | -24.625 | *N. lotus* | 44.318 | -23.565 | *N. macrosperma* | 132.2 | -12.37 |
| *N. lotus* | 20.875 | -19.63 | *N. lotus* | 31.375 | -27.875 | *N. lotus* | 45.014 | -20.404 | *N. macrosperma* | 130.168 | -15.482 |
| *N. lotus* | 21.375 | -18.13 | *N. lotus* | 31.484 | -25.102 | *N. lotus* | 45.474 | -19.708 | *N. macrosperma* | 132.28 | -12.58 |
| *N. lotus* | 21.881 | -18.41 | *N. lotus* | 31.566 | -24.982 | *N. lotus* | 46.856 | -19.825 | *N. macrosperma* | 132.85 | -12.45 |
| *N. lotus* | 22.017 | -18.5 | *N. lotus* | 31.625 | -28.625 | *N. lotus* | 47.09 | -20.196 | *N. macrosperma* | 143.926 | -14.607 |
| *N. lotus* | 22.157 | -18.56 | *N. lotus* | 31.652 | -29.006 | *N. lotus* | 47.147 | -24.77 | *N. macrosperma* | 130.401 | -15.499 |
| *N. lotus* | 22.196 | -18.74 | *N. lotus* | 31.756 | -28.918 | *N. lotus* | 47.483 | -19.2 | *N. macrosperma* | 141.143 | -17.873 |
| *N. lotus* | 22.375 | -19.13 | *N. lotus* | 31.875 | -28.875 | *N. lotus* | 48.249 | -18.451 | *N. macrosperma* | 132.835 | -12.632 |
| *N. lotus* | 22.386 | -18.96 | *N. lotus* | 31.955 | -28.154 | *N. lotus* | 48.268 | -18.205 | *N. macrosperma* | 132.935 | -12.682 |
| *N. lotus* | 22.406 | -18.86 | *N. lotus* | 31.956 | -25.448 | *N. lotus* | 48.297 | -18.868 | *N. macrosperma* | 141.834 | -13.349 |
| *N. lotus* | 22.625 | -19.38 | *N. lotus* | 31.982 | -26.156 | *N. lotus* | 48.686 | -13.59 | *N. macrosperma* | 132.085 | -14.699 |
| *N. lotus* | 23.125 | -19.63 | *N. lotus* | 31.987 | -28.682 | *N. lotus* | 48.791 | -19.898 | *N. macrosperma* | 133 | -12.333 |
| *N. lotus* | 23.125 | -19.13 | *N. lotus* | 32.095 | -28.108 | *N. lotus* | 28.837 | -16.19 | *N. macrosperma* | 143.918 | -15.215 |
| *N. lotus* | 23.296 | -17.98 | *N. lotus* | 32.108 | 14.167 | *N. lotus* | 30.14 | -12.502 | *N. macrosperma* | 134.051 | -12.232 |
| *N. lotus* | 23.3 | -17.7 | *N. lotus* | 32.125 | -28.625 | *N. lotus* | 26.01 | -15 | *N. macrosperma* | 131.218 | -12.365 |
| *N. lotus* | 23.375 | -19.88 | *N. lotus* | 32.125 | -26.875 | *N. lotus* | 23.221 | -13.322 | *N. macrosperma* | 141.801 | -15.199 |
| *N. lotus* | 23.375 | -16.13 | *N. lotus* | 32.135 | -27.383 | *N. lotus* | 28.888 | -15.983 | *N. macrosperma* | 144.705 | -15.281 |
| *N. lotus* | 23.379 | -18.12 | *N. lotus* | 32.375 | -28.375 | *N. macrosperma* | 143.664 | -14.226 | *N. macrosperma* | 128.267 | -15.483 |
| *N. lotus* | 23.545 | -18.56 | *N. lotus* | 32.375 | -26.875 | *N. macrosperma* | 132.886 | -12.079 | *N. macrosperma* | 133.117 | -14.933 |
| *N. lotus* | 23.625 | -19.88 | *N. lotus* | 32.406 | -27.657 | *N. macrosperma* | 132.219 | -12.571 | *N. macrosperma* | 141.084 | -17.582 |
| *N. lotus* | 23.726 | 1.248 | *N. lotus* | 32.5 | -25.283 | *N. macrosperma* | 132.517 | -12.9 | *N. macrosperma* | 128.258 | -15.588 |
| *N. lotus* | 23.962 | -19.23 | *N. lotus* | 32.625 | -26.875 | *N. macrosperma* | 132.672 | -15.654 | *N. macrosperma* | 138.58 | -17.88 |
| *N. lotus* | 24.821 | -17.92 | *N. lotus* | 32.818 | -27.021 | *N. macrosperma* | 132.751 | -15.483 | *N. macrosperma* | 132.95 | -12.43 |
| *N. lotus* | 24.875 | -21.13 | *N. lotus* | 32.875 | -26.875 | *N. macrosperma* | 131.349 | -12.556 | *N. macrosperma* | 136.385 | -15.649 |
| *N. lotus* | 25.144 | -17.81 | *N. lotus* | 33.272 | -18.987 | *N. macrosperma* | 132.883 | -12.553 | *N. macrosperma* | 131.117 | -12.583 |
| *N. lotus* | 25.851 | -17.91 | *N. lotus* | 34.017 | 2.083 | *N. macrosperma* | 135.9 | -13.226 | *N. macrosperma* | 131.251 | -12.632 |
| *N. lotus* | 27.11 | -25.25 | *N. lotus* | 34.024 | -9.488 | *N. macrosperma* | 132.743 | -12.845 | *N. macrosperma* | 142.259 | -15.84 |
| *N. lotus* | 27.625 | -24.38 | *N. lotus* | 34.03 | -15.877 | *N. macrosperma* | 129.011 | -15.622 | *N. macrosperma* | 131.585 | -12.749 |
| *N. lotus* | 28.125 | -25.63 | *N. lotus* | 34.169 | 0.021 | *N. macrosperma* | 128.31 | -15.563 | *N. macrosperma* | 131.538 | -12.321 |
| *N. lotus* | 28.625 | -25.13 | *N. lotus* | 34.65 | 8.283 | *N. macrosperma* | 130.751 | -13.749 | *N. macrosperma* | 130.274 | -13.389 |
| *N. lotus* | 28.933 | -8.417 | *N. lotus* | 35.099 | -16.515 | *N. macrosperma* | 133.018 | -12.232 | *N. macrosperma* | 131.509 | -12.649 |
| *N. lotus* | 29.333 | -3.25 | *N. lotus* | 36.017 | 0.583 | *N. macrosperma* | 132.585 | -12.815 | *N. macrosperma* | 131.613 | -12.318 |
| *N. lotus* | 29.375 | -25.38 | *N. lotus* | 36.399 | -17.731 | *N. macrosperma* | 131.823 | -13.09 | *N. macrosperma* | 130.387 | -13.323 |
| *N. lotus* | 29.517 | 3.753 | *N. lotus* | 37.417 | -3.75 | *N. macrosperma* | 132.583 | -12.75 | *N. macrosperma* | 130.508 | -13.019 |
| *N. lotus* | 29.7 | 1.383 | *N. lotus* | 37.417 | 11.6 | *N. macrosperma* | 132.451 | -12.849 | *N. macrosperma* | 133.727 | -11.976 |
| *N. lotus* | 30.375 | -23.38 | *N. lotus* | 37.5 | 12 | *N. macrosperma* | 130.588 | -13.751 | *N. macrosperma* | 131.654 | -12.355 |
| *N. macrosperma* | 130.381 | -13.17 | *N. mexicana* | 151.374 | -33.516 | *N. micrantha* | -15.083 | 11.777 | *N. micrantha* | -2.334 | 13.529 |
| *N. macrosperma* | 130.441 | -13.27 | *N. mexicana* | 151.242 | -33.772 | *N. micrantha* | -14.952 | 12.03 | *N. micrantha* | -1.917 | 16.867 |
| *N. macrosperma* | 135.039 | -12.44 | *N. mexicana* | 151.293 | -33.359 | *N. micrantha* | -14.901 | 11.136 | *N. micrantha* | -1.6 | 14.05 |
| *N. macrosperma* | 134.967 | -12.34 | *N. mexicana* | 152.432 | -31.876 | *N. micrantha* | -14.574 | 12.395 | *N. micrantha* | -1.517 | 12.233 |
| *N. macrosperma* | 132.419 | -14.88 | *N. mexicana* | 138.534 | -34.925 | *N. micrantha* | -14.048 | 11.843 | *N. micrantha* | -1.4 | 12.633 |
| *N. macrosperma* | 132.694 | -13.05 | *N. mexicana* | 116.35 | -34.583 | *N. micrantha* | -13.016 | 13.3 | *N. micrantha* | -1.3 | 12.033 |
| *N. macrosperma* | 132.669 | -12.65 | *N. mexicana* | 145.135 | -36.753 | *N. micrantha* | -12.569 | 11.194 | *N. micrantha* | -1.25 | 16.233 |
| *N. macrosperma* | 132.902 | -12.65 | *N. mexicana* | 151.482 | -33.242 | *N. micrantha* | -12.25 | 12.533 | *N. micrantha* | -1.167 | 9.167 |
| *N. macrosperma* | 131.652 | -12.7 | *N. mexicana* | 150.49 | -33.728 | *N. micrantha* | -12.167 | 8.317 | *N. micrantha* | -0.65 | 8.217 |
| *N. macrosperma* | 132.202 | -12.61 | *N. mexicana* | 148.034 | -37.745 | *N. micrantha* | -12.13 | 8.503 | *N. micrantha* | -0.5 | 13.317 |
| *N. macrosperma* | 134.969 | -12.48 | *N. mexicana* | 150.861 | -33.558 | *N. micrantha* | -11.283 | 13.341 | *N. micrantha* | -0.467 | 14.667 |
| *N. macrosperma* | 130.719 | -13.66 | *N. mexicana* | 145.118 | -36.832 | *N. micrantha* | -10.367 | 16.033 | *N. micrantha* | -0.428 | 11.159 |
| *N. macrosperma* | 133.285 | -14.91 | *N. mexicana* | 115.337 | -33.651 | *N. micrantha* | -8.383 | 12.267 | *N. micrantha* | -0.316 | 11.716 |
| *N. macrosperma* | 136.369 | -16.08 | *N. mexicana* | 152.67 | -25.53 | *N. micrantha* | -7.933 | 12.65 | *N. micrantha* | -0.264 | 14.376 |
| *N. macrosperma* | 130.686 | -13.75 | *N. mexicana* | 116 | -31.817 | *N. micrantha* | -7.783 | 12.667 | *N. micrantha* | -0.133 | 12.133 |
| *N. macrosperma* | 133.419 | -14.88 | *N. mexicana* | 151.218 | -33.898 | *N. micrantha* | -7.597 | 13.25 | *N. micrantha* | -0.083 | 14.067 |
| *N. mexicana* | 152.57 | -25.98 | *N. mexicana* | 153.084 | -27.415 | *N. micrantha* | -6.917 | 4.667 | *N. micrantha* | -0.05 | 16.267 |
| *N. mexicana* | 152.654 | -25.58 | *N. mexicana* | 151.018 | -33.815 | *N. micrantha* | -6.258 | 13.446 | *N. micrantha* | 0.004 | 8.51 |
| *N. mexicana* | 153.076 | -27.66 | *N. mexicana* | 151.168 | -33.815 | *N. micrantha* | -6.017 | 14.683 | *N. micrantha* | 0.026 | 11.01 |
| *N. mexicana* | 152.685 | -26.22 | *N. mexicana* | 151.201 | -33.948 | *N. micrantha* | -6 | 14.3 | *N. micrantha* | 0.07 | 7.53 |
| *N. mexicana* | 152.924 | -27.44 | *N. mexicana* | 151 | -34 | *N. micrantha* | -5.901 | 14.532 | *N. micrantha* | 0.117 | 7.883 |
| *N. mexicana* | 150.917 | -33.67 | *N. mexicana* | 152.5 | -27.5 | *N. micrantha* | -5.6 | 13.95 | *N. micrantha* | 0.36 | 12.06 |
| *N. mexicana* | 153.248 | -27.51 | *N. mexicana* | 151.22 | -33.83 | *N. micrantha* | -5.45 | 11.217 | *N. micrantha* | 0.39 | 8.13 |
| *N. mexicana* | 152.878 | -27.46 | *N. micrantha* | 10.518 | 11.255 | *N. micrantha* | -5.033 | 10.981 | *N. micrantha* | 0.467 | 10.35 |
| *N. mexicana* | 145.5 | -38.03 | *N. micrantha* | 9.852 | 10.41 | *N. micrantha* | -4.853 | 10.644 | *N. micrantha* | 0.49 | 9.41 |
| *N. mexicana* | 150.661 | -33.62 | *N. micrantha* | 6.684 | 12.196 | *N. micrantha* | -4.6 | 15.617 | *N. micrantha* | 1.1 | 9.6 |
| *N. mexicana* | 151.207 | -33.56 | *N. micrantha* | 9.983 | 12.423 | *N. micrantha* | -4.333 | 5.333 | *N. micrantha* | 1.667 | 6.25 |
| *N. mexicana* | 150.699 | -33.71 | *N. micrantha* | 10.228 | 12.648 | *N. micrantha* | -4.183 | 9.233 | *N. micrantha* | 1.717 | 6.9 |
| *N. mexicana* | 139.367 | -35.05 | *N. micrantha* | 8.927 | 9.879 | *N. micrantha* | -4.183 | 9.5 | *N. micrantha* | 2.134 | 6.88 |
| *N. mexicana* | 151.654 | -32.77 | *N. micrantha* | 7.312 | 11.716 | *N. micrantha* | -4.167 | 13.117 | *N. micrantha* | 2.383 | 12.35 |
| *N. mexicana* | 139.606 | -34.85 | *N. micrantha* | 8.684 | 11.858 | *N. micrantha* | -4.05 | 5.367 | *N. micrantha* | 2.417 | 12.467 |
| *N. mexicana* | 145.985 | -36.55 | *N. micrantha* | 4.227 | 12.479 | *N. micrantha* | -3.983 | 5.267 | *N. micrantha* | 2.5 | 9.083 |
| *N. mexicana* | 139.619 | -34.34 | *N. micrantha* | 7.395 | 11.099 | *N. micrantha* | -3.467 | 12.95 | *N. micrantha* | 3.183 | 12.067 |
| *N. mexicana* | 144.37 | -35.96 | *N. micrantha* | 11.048 | 12.257 | *N. micrantha* | -3.467 | 16.083 | *N. micrantha* | 3.387 | 11.81 |
| *N. mexicana* | 144.306 | -34.48 | *N. micrantha* | -17.083 | 14.917 | *N. micrantha* | -3.433 | 12.833 | *N. micrantha* | 4.083 | 15.083 |
| *N. mexicana* | 150.925 | -33.35 | *N. micrantha* | -16.644 | 12.475 | *N. micrantha* | -3.417 | 13.2 | *N. micrantha* | 4.517 | 10.35 |
| *N. mexicana* | 151.412 | -33.4 | *N. micrantha* | -16.626 | 13.383 | *N. micrantha* | -2.7 | 10.833 | *N. micrantha* | 4.75 | 13.833 |
| *N. mexicana* | 151.478 | -33.06 | *N. micrantha* | -16.518 | 12.32 | *N. micrantha* | -2.6 | 12.2 | *N. micrantha* | 4.833 | 13.983 |
| *N. mexicana* | 150.618 | -33.76 | *N. micrantha* | -16.367 | 12.533 | *N. micrantha* | -2.583 | 7.45 | *N. micrantha* | 5.167 | 14.367 |
| *N. mexicana* | 151.493 | -33.17 | *N. micrantha* | -16.299 | 16.414 | *N. micrantha* | -2.42 | 10.5 | *N. micrantha* | 5.267 | 14.283 |
| *N. mexicana* | 151.353 | -32.82 | *N. micrantha* | -15.941 | 11.468 | *N. micrantha* | -2.35 | 7.27 | *N. micrantha* | 5.533 | 13.917 |
| *N. micrantha* | 5.65 | 15.267 | *N. nouchali* | 9.448 | -0.676 | *N. nouchali* | 20.986 | -33.504 | *N. nouchali* | 23.625 | -18.375 |
| *N. micrantha* | 5.7 | 15.383 | *N. nouchali* | 11.767 | -0.7 | *N. nouchali* | 21.375 | -18.125 | *N. nouchali* | 23.626 | -19.391 |
| *N. micrantha* | 5.7 | 15.55 | *N. nouchali* | 11.8 | -4.687 | *N. nouchali* | 21.625 | -18.125 | *N. nouchali* | 24.067 | 12.917 |
| *N. micrantha* | 5.75 | 15.25 | *N. nouchali* | 13.237 | -0.903 | *N. nouchali* | 21.745 | -18.207 | *N. nouchali* | 24.197 | -33.916 |
| *N. micrantha* | 6.558 | 9.049 | *N. nouchali* | 13.695 | 7.131 | *N. nouchali* | 21.875 | -18.375 | *N. nouchali* | 24.375 | -18.625 |
| *N. micrantha* | 7.63 | 11.182 | *N. nouchali* | 13.994 | -1.771 | *N. nouchali* | 22.017 | -18.496 | *N. nouchali* | 24.375 | -17.875 |
| *N. micrantha* | 7.762 | 11.082 | *N. nouchali* | 14.083 | 13.633 | *N. nouchali* | 22.125 | -19.375 | *N. nouchali* | 24.488 | -34.01 |
| *N. micrantha* | 10.401 | 12.841 | *N. nouchali* | 14.55 | 11.4 | *N. nouchali* | 22.125 | -18.625 | *N. nouchali* | 24.625 | -34.125 |
| *N. micrantha* | 10.5 | 9.75 | *N. nouchali* | 14.875 | -17.375 | *N. nouchali* | 22.195 | -18.744 | *N. nouchali* | 24.757 | -17.895 |
| *N. micrantha* | 12.183 | 13.15 | *N. nouchali* | 15.125 | -17.625 | *N. nouchali* | 22.373 | -18.788 | *N. nouchali* | 24.875 | -21.125 |
| *N. micrantha* | 12.633 | 6.467 | *N. nouchali* | 15.133 | 11.983 | *N. nouchali* | 22.373 | -18.951 | *N. nouchali* | 25.041 | -33.85 |
| *N. micrantha* | 12.95 | 9.317 | *N. nouchali* | 15.709 | -17.74 | *N. nouchali* | 22.375 | -19.875 | *N. nouchali* | 25.093 | -28.999 |
| *N. micrantha* | 13.4 | 9.3 | *N. nouchali* | 15.875 | -17.875 | *N. nouchali* | 22.375 | -19.125 | *N. nouchali* | 25.125 | -33.625 |
| *N. micrantha* | 13.567 | 10.617 | *N. nouchali* | 17.625 | -18.625 | *N. nouchali* | 22.419 | -33.966 | *N. nouchali* | 25.125 | -17.875 |
| *N. micrantha* | 13.617 | 9.383 | *N. nouchali* | 18.125 | -19.625 | *N. nouchali* | 22.601 | -19.282 | *N. nouchali* | 25.158 | -33.898 |
| *N. micrantha* | 13.633 | 10.733 | *N. nouchali* | 18.416 | -34.051 | *N. nouchali* | 22.625 | -19.125 | *N. nouchali* | 25.263 | -33.915 |
| *N. micrantha* | 14.3 | 12.7 | *N. nouchali* | 18.517 | -34.067 | *N. nouchali* | 22.643 | -19.514 | *N. nouchali* | 25.375 | -33.625 |
| *N. micrantha* | 14.683 | 11.233 | *N. nouchali* | 18.633 | -34.033 | *N. nouchali* | 22.65 | -33.984 | *N. nouchali* | 25.4 | -33.75 |
| *N. micrantha* | 14.817 | 12.183 | *N. nouchali* | 18.783 | -31.81 | *N. nouchali* | 22.791 | -19.527 | *N. nouchali* | 25.433 | -33.952 |
| *N. micrantha* | 15 | 12.083 | *N. nouchali* | 18.828 | -33.937 | *N. nouchali* | 22.802 | -19.316 | *N. nouchali* | 25.545 | -33.389 |
| *N. micrantha* | 15.05 | 12.333 | *N. nouchali* | 18.833 | -32.083 | *N. nouchali* | 22.845 | -19.742 | *N. nouchali* | 25.625 | -33.875 |
| *N. micrantha* | 15.1 | 11.783 | *N. nouchali* | 18.872 | -34.063 | *N. nouchali* | 22.865 | -18.988 | *N. nouchali* | 25.625 | -18.625 |
| *N. micrantha* | 15.1 | 12.067 | *N. nouchali* | 18.946 | -33.735 | *N. nouchali* | 22.9 | -33.9 | *N. nouchali* | 26 | -30 |
| *N. micrantha* | 15.333 | 11.2 | *N. nouchali* | 19.022 | -34.196 | *N. nouchali* | 22.94 | -19.415 | *N. nouchali* | 26.125 | -33.375 |
| *N. micrantha* | 15.5 | 10.583 | *N. nouchali* | 19.031 | -34.327 | *N. nouchali* | 22.999 | -33.981 | *N. nouchali* | 26.125 | -29.375 |
| *N. micrantha* | 15.55 | 10.083 | *N. nouchali* | 19.16 | -32.178 | *N. nouchali* | 23.001 | -19.907 | *N. nouchali* | 26.404 | -33.405 |
| *N. micrantha* | 15.75 | 10.767 | *N. nouchali* | 19.232 | -34.377 | *N. nouchali* | 23.064 | -19.544 | *N. nouchali* | 26.586 | -33.485 |
| *N. micrantha* | 15.817 | 9.733 | *N. nouchali* | 19.375 | -33.625 | *N. nouchali* | 23.125 | -19.875 | *N. nouchali* | 26.625 | -25.875 |
| *N. micrantha* | 16.483 | 9.067 | *N. nouchali* | 19.522 | -34.409 | *N. nouchali* | 23.125 | -19.125 | *N. nouchali* | 26.625 | -25.625 |
| *N. micrantha* | 16.817 | 9.067 | *N. nouchali* | 19.61 | -34.07 | *N. nouchali* | 23.137 | -19.657 | *N. nouchali* | 26.95 | -10.85 |
| *N. micrantha* | 17.467 | 12.883 | *N. nouchali* | 19.625 | -34.625 | *N. nouchali* | 23.151 | -20.133 | *N. nouchali* | 27.125 | -26.625 |
| *N. micrantha* | 18.133 | 9.217 | *N. nouchali* | 19.857 | -17.898 | *N. nouchali* | 23.167 | -19.554 | *N. nouchali* | 27.375 | -32.875 |
| *N. micrantha* | 19.917 | 10.717 | *N. nouchali* | 19.875 | -34.625 | *N. nouchali* | 23.245 | -19.165 | *N. nouchali* | 27.417 | -11.717 |
| *N. micrantha* | 20.033 | 11.283 | *N. nouchali* | 20.061 | -17.513 | *N. nouchali* | 23.318 | -19.875 | *N. nouchali* | 27.511 | -33.187 |
| *N. micrantha* | 20.067 | 10.75 | *N. nouchali* | 20.125 | -17.875 | *N. nouchali* | 23.357 | -19.128 | *N. nouchali* | 27.625 | -26.875 |
| *N. nouchali* | -16 | 16.35 | *N. nouchali* | 20.129 | -33.792 | *N. nouchali* | 23.375 | -27.375 | *N. nouchali* | 27.625 | -26.375 |
| *N. nouchali* | -15.009 | 11.777 | *N. nouchali* | 20.37 | -33.993 | *N. nouchali* | 23.379 | -18.117 | *N. nouchali* | 27.625 | -24.375 |
| *N. nouchali* | -13.019 | 11.834 | *N. nouchali* | 20.625 | -34.125 | *N. nouchali* | 23.38 | -19.242 | *N. nouchali* | 27.625 | -23.625 |
| *N. nouchali* | -4.833 | 10.667 | *N. nouchali* | 20.625 | -19.375 | *N. nouchali* | 23.494 | -33.979 | *N. nouchali* | 27.672 | -33.156 |
| *N. nouchali* | 2.343 | 9.647 | *N. nouchali* | 20.875 | -20.875 | *N. nouchali* | 23.625 | -19.875 | *N. nouchali* | 27.84 | -26.088 |
| *N. nouchali* | 9.338 | -0.377 | *N. nouchali* | 20.875 | -18.125 | *N. nouchali* | 23.625 | -19.125 | *N. nouchali* | 27.875 | -26.875 |
| *N. nouchali* | 27.875 | -24.13 | *N. nouchali* | 30.15 | -31.017 | *N. nouchali* | 31.625 | -24.875 | *N. nouchali* | 32.783 | -18.283 |
| *N. nouchali* | 27.875 | -23.63 | *N. nouchali* | 30.375 | -29.875 | *N. nouchali* | 31.652 | -29.006 | *N. nouchali* | 32.8 | -27.033 |
| *N. nouchali* | 27.93 | -32.58 | *N. nouchali* | 30.375 | -29.625 | *N. nouchali* | 31.687 | -7.983 | *N. nouchali* | 33.534 | -24.914 |
| *N. nouchali* | 28.125 | -25.88 | *N. nouchali* | 30.375 | -22.875 | *N. nouchali* | 31.742 | -24.106 | *N. nouchali* | 33.633 | -25.1 |
| *N. nouchali* | 28.125 | -25.63 | *N. nouchali* | 30.386 | -30.82 | *N. nouchali* | 31.756 | -28.918 | *N. nouchali* | 33.665 | -16.228 |
| *N. nouchali* | 28.203 | -32.74 | *N. nouchali* | 30.45 | -1.52 | *N. nouchali* | 31.764 | -25.291 | *N. nouchali* | 33.921 | -24.745 |
| *N. nouchali* | 28.217 | -24.76 | *N. nouchali* | 30.498 | -29.771 | *N. nouchali* | 31.79 | -1.327 | *N. nouchali* | 34.06 | -14.964 |
| *N. nouchali* | 28.375 | -25.88 | *N. nouchali* | 30.533 | -2.167 | *N. nouchali* | 31.875 | -28.875 | *N. nouchali* | 34.524 | -18.889 |
| *N. nouchali* | 28.375 | -25.63 | *N. nouchali* | 30.625 | -30.375 | *N. nouchali* | 31.875 | -27.375 | *N. nouchali* | 34.749 | -5.003 |
| *N. nouchali* | 28.375 | -25.38 | *N. nouchali* | 30.625 | -23.625 | *N. nouchali* | 31.875 | -27.125 | *N. nouchali* | 34.851 | -5.026 |
| *N. nouchali* | 28.375 | -24.63 | *N. nouchali* | 30.625 | -22.875 | *N. nouchali* | 31.875 | -24.875 | *N. nouchali* | 35 | -6 |
| *N. nouchali* | 28.375 | -24.13 | *N. nouchali* | 30.732 | -20.129 | *N. nouchali* | 31.875 | -24.375 | *N. nouchali* | 35.15 | -22.078 |
| *N. nouchali* | 28.375 | -23.88 | *N. nouchali* | 30.743 | -30.3 | *N. nouchali* | 31.982 | -26.156 | *N. nouchali* | 35.167 | -8.517 |
| *N. nouchali* | 28.451 | -15.47 | *N. nouchali* | 30.83 | -24.25 | *N. nouchali* | 31.991 | -28.703 | *N. nouchali* | 35.241 | -13.313 |
| *N. nouchali* | 28.48 | -2.15 | *N. nouchali* | 30.875 | -29.875 | *N. nouchali* | 32.019 | -26.283 | *N. nouchali* | 36.35 | -0.76 |
| *N. nouchali* | 28.533 | 7.433 | *N. nouchali* | 30.875 | -25.375 | *N. nouchali* | 32.036 | -25.956 | *N. nouchali* | 36.399 | -17.731 |
| *N. nouchali* | 28.625 | -24.63 | *N. nouchali* | 30.875 | -24.375 | *N. nouchali* | 32.108 | 14.167 | *N. nouchali* | 36.75 | 7.617 |
| *N. nouchali* | 28.625 | -24.38 | *N. nouchali* | 30.875 | -22.375 | *N. nouchali* | 32.125 | -28.625 | *N. nouchali* | 36.825 | -1.242 |
| *N. nouchali* | 28.816 | -32.31 | *N. nouchali* | 30.96 | -25.678 | *N. nouchali* | 32.125 | -28.375 | *N. nouchali* | 36.857 | -17.745 |
| *N. nouchali* | 28.875 | -25.88 | *N. nouchali* | 30.975 | -29.896 | *N. nouchali* | 32.125 | -27.875 | *N. nouchali* | 37.05 | 7.68 |
| *N. nouchali* | 28.875 | -25.63 | *N. nouchali* | 31.125 | -28.625 | *N. nouchali* | 32.125 | -27.375 | *N. nouchali* | 37.125 | -12.125 |
| *N. nouchali* | 28.875 | -24.63 | *N. nouchali* | 31.125 | -26.125 | *N. nouchali* | 32.125 | -26.625 | *N. nouchali* | 37.199 | -2.639 |
| *N. nouchali* | 29 | -19 | *N. nouchali* | 31.125 | -25.125 | *N. nouchali* | 32.125 | -26.125 | *N. nouchali* | 37.333 | -13.317 |
| *N. nouchali* | 29.125 | -24.13 | *N. nouchali* | 31.125 | -24.625 | *N. nouchali* | 32.182 | -28.484 | *N. nouchali* | 37.492 | 11.514 |
| *N. nouchali* | 29.125 | -23.13 | *N. nouchali* | 31.125 | -24.125 | *N. nouchali* | 32.244 | -27.954 | *N. nouchali* | 37.5 | 12 |
| *N. nouchali* | 29.261 | -26.19 | *N. nouchali* | 31.151 | -17.836 | *N. nouchali* | 32.25 | -27.25 | *N. nouchali* | 37.52 | -17.334 |
| *N. nouchali* | 29.375 | -31.63 | *N. nouchali* | 31.179 | -26.487 | *N. nouchali* | 32.35 | -27.833 | *N. nouchali* | 37.6 | -17.483 |
| *N. nouchali* | 29.375 | -25.38 | *N. nouchali* | 31.186 | -6.899 | *N. nouchali* | 32.361 | -28.331 | *N. nouchali* | 37.833 | 6.583 |
| *N. nouchali* | 29.375 | -23.13 | *N. nouchali* | 31.375 | -28.875 | *N. nouchali* | 32.375 | -28.125 | *N. nouchali* | 38.231 | -7.748 |
| *N. nouchali* | 29.5 | -2.55 | *N. nouchali* | 31.375 | -28.375 | *N. nouchali* | 32.375 | -27.625 | *N. nouchali* | 38.417 | 7 |
| *N. nouchali* | 29.833 | -2.917 | *N. nouchali* | 31.375 | -26.875 | *N. nouchali* | 32.375 | -27.125 | *N. nouchali* | 38.6 | -6.15 |
| *N. nouchali* | 29.875 | -31.38 | *N. nouchali* | 31.375 | -25.375 | *N. nouchali* | 32.483 | -27.017 | *N. nouchali* | 38.833 | 7.85 |
| *N. nouchali* | 29.875 | -23.88 | *N. nouchali* | 31.375 | -25.125 | *N. nouchali* | 32.491 | -28.23 | *N. nouchali* | 38.867 | -6.833 |
| *N. nouchali* | 29.875 | -23.38 | *N. nouchali* | 31.375 | -24.375 | *N. nouchali* | 32.5 | -27.5 | *N. nouchali* | 38.9 | -5.15 |
| *N. nouchali* | 29.917 | -1.283 | *N. nouchali* | 31.375 | -22.625 | *N. nouchali* | 32.583 | 0.367 | *N. nouchali* | 39 | 7.933 |
| *N. nouchali* | 29.981 | -31.3 | *N. nouchali* | 31.389 | -24.183 | *N. nouchali* | 32.584 | -27.665 | *N. nouchali* | 39.05 | -5.033 |
| *N. nouchali* | 30 | 15 | *N. nouchali* | 31.478 | -29.095 | *N. nouchali* | 32.625 | -27.375 | *N. nouchali* | 39.183 | -6.9 |
| *N. nouchali* | 30.125 | -31.13 | *N. nouchali* | 31.589 | -24.995 | *N. nouchali* | 32.635 | -27.557 | *N. nouchali* | 39.217 | -6.15 |
| *N. nouchali* | 30.125 | -23.88 | *N. nouchali* | 31.623 | -25.279 | *N. nouchali* | 32.676 | -25.737 | *N. nouchali* | 39.233 | -6.8 |
| *N. nouchali* | 30.125 | -23.63 | *N. nouchali* | 31.625 | -27.375 | *N. nouchali* | 32.683 | -27.282 | *N. nouchali* | 39.25 | -5.95 |
| *N. nouchali* | 30.125 | -23.38 | *N. nouchali* | 31.625 | -26.375 | *N. nouchali* | 32.783 | -26.85 | *N. nouchali* | 39.317 | -15.472 |
| *N. nouchali* | 39.483 | -15.59 | *N. nouchali* | 49.078 | -13.2 | *N. nouchali* | 22.613 | -14.925 | *N. nouchali* | 153.521 | -28.21 |
| *N. nouchali* | 39.8 | -15.98 | *N. nouchali* | 49.149 | -12.58 | *N. nouchali* | 24.34 | -14.958 | *N. nouchali* | 152.596 | -25.543 |
| *N. nouchali* | 40.65 | 10.167 | *N. nouchali* | 49.383 | -17.283 | *N. nouchali* | 24.524 | -14.665 | *N. nouchali* | 152.717 | -25.847 |
| *N. nouchali* | 40.656 | -2.513 | *N. nouchali* | 49.668 | -16.864 | *N. nouchali* | 24.191 | -14.487 | *N. nouchali* | 152.977 | -27.369 |
| *N. nouchali* | 41.412 | 11.625 | *N. nouchali* | 49.685 | -16.744 | *N. nouchali* | 23.24 | -13.288 | *N. nouchali* | 153.092 | -30.308 |
| *N. nouchali* | 43.678 | -21.87 | *N. nouchali* | 21.15 | 8.483 | *N. nouchali* | 25.574 | -12.243 | *N. nouchali* | 153.109 | -26.814 |
| *N. nouchali* | 43.736 | -21.71 | *N. nouchali* | 15.45 | -5.3 | *N. nouchali* | 24.441 | -11.737 | *N. nouchali* | 152.68 | -25.751 |
| *N. nouchali* | 44.155 | -22.72 | *N. nouchali* | 29.773 | -1.307 | *N. nouchali* | 24.997 | -11.761 | *N. nouchali* | 145.699 | -16.834 |
| *N. nouchali* | 44.167 | 1.583 | *N. nouchali* | 39.057 | -6.578 | *N. nouchali* | 30.808 | -14.551 | *N. nouchali* | 146.283 | -18.7 |
| *N. nouchali* | 44.403 | -20.27 | *N. nouchali* | 38.854 | -5.308 | *N. nouchali* | 28.888 | -15.983 | *N. nouchali* | 146.05 | -17.983 |
| *N. nouchali* | 44.705 | -18.7 | *N. nouchali* | 38.68 | -4.766 | *N. nouchali* | 28.688 | -13.301 | *N. nouchali* | 145.258 | -15.492 |
| *N. nouchali* | 45.283 | -16.35 | *N. nouchali* | 33.851 | -9.583 | *N. nouchali* | 26.855 | -12.918 | *N. nouchali* | 145.4 | -16.267 |
| *N. nouchali* | 45.397 | -22.56 | *N. nouchali* | 39.804 | -3.403 | *N. nouchali* | 27.603 | -12.867 | *N. nouchali* | 145.75 | -16.97 |
| *N. nouchali* | 46.033 | -25.02 | *N. nouchali* | 38.389 | -5.076 | *N. nouchali* | 152.69 | -25.544 | *N. nouchali* | 132.869 | -12.464 |
| *N. nouchali* | 46.125 | -16.26 | *N. nouchali* | 29.673 | -1.259 | *N. nouchali* | 152.654 | -25.578 | *N. nouchali* | 130.967 | -13.433 |
| *N. nouchali* | 46.143 | -24.25 | *N. nouchali* | 35.753 | -3.703 | *N. nouchali* | 152.971 | -27.539 | *N. nouchali* | 136.297 | -12.438 |
| *N. nouchali* | 46.683 | -25.05 | *N. nouchali* | 37.351 | -11.117 | *N. nouchali* | 152.829 | -27.521 | *N. nouchali* | 145.744 | -16.882 |
| *N. nouchali* | 46.706 | -19.37 | *N. nouchali* | 29.818 | -12.108 | *N. nouchali* | 152.769 | -26.602 | *N. nouchali* | 146.632 | -19.181 |
| *N. nouchali* | 46.755 | -19.18 | *N. nouchali* | 23.522 | -16.524 | *N. nouchali* | 151.221 | -23.969 | *N. nouchali* | 145.415 | -16.344 |
| *N. nouchali* | 46.882 | -21.83 | *N. nouchali* | 30.452 | -0.597 | *N. nouchali* | 152.769 | -25.277 | *N. nouchali* | 131.308 | -12.568 |
| *N. nouchali* | 47 | -24.95 | *N. nouchali* | 34.739 | -0.142 | *N. nouchali* | 153.168 | -27.55 | *N. nouchali* | 129.658 | -15.764 |
| *N. nouchali* | 47.064 | -20.67 | *N. nouchali* | 33.145 | -0.78 | *N. nouchali* | 152.975 | -27.476 | *N. nouchali* | 146.018 | -18.051 |
| *N. nouchali* | 47.089 | -18.91 | *N. nouchali* | 30.345 | -0.367 | *N. nouchali* | 152.122 | -27.563 | *N. nouchali* | 146.426 | -19.057 |
| *N. nouchali* | 47.09 | -20.2 | *N. nouchali* | 32.876 | -1.433 | *N. nouchali* | 153.299 | -28.46 | *N. nouchali* | 145.572 | -16.673 |
| *N. nouchali* | 47.119 | -24.82 | *N. nouchali* | 31.6 | 0.112 | *N. nouchali* | 153.149 | -27.435 | *N. nouchali* | 152.922 | -27.441 |
| *N. nouchali* | 47.19 | -24.7 | *N. nouchali* | 35.614 | -0.01 | *N. nouchali* | 152.964 | -26.835 | *N. nouchali* | 153.372 | -28.087 |
| *N. nouchali* | 47.25 | -21.25 | *N. nouchali* | 31.714 | -0.336 | *N. nouchali* | 152.682 | -26.444 | *N. nouchali* | 152.926 | -27.307 |
| *N. nouchali* | 47.3 | -19.37 | *N. nouchali* | 36.661 | -1.252 | *N. nouchali* | 153.267 | -27.534 | *N. nouchali* | 153.011 | -30.505 |
| *N. nouchali* | 47.375 | -22.44 | *N. nouchali* | 31.115 | -0.338 | *N. nouchali* | 142.087 | -11.989 | *N. nouchali* | 152.786 | -27.806 |
| *N. nouchali* | 47.417 | -21.25 | *N. nouchali* | 30.151 | -12.355 | *N. nouchali* | 153.467 | -28.2 | *N. nouchali* | 153.347 | -27.941 |
| *N. nouchali* | 47.433 | -14.76 | *N. nouchali* | 30.144 | -11.571 | *N. nouchali* | 146.151 | -18.655 | *N. nouchali* | 152.9 | -27.4 |
| *N. nouchali* | 47.517 | -18.92 | *N. nouchali* | 30.363 | -12.218 | *N. nouchali* | 146.765 | -19.313 | *N. nouchali* | 152.573 | -31.973 |
| *N. nouchali* | 47.99 | -13.67 | *N. nouchali* | 25.115 | -16.842 | *N. nouchali* | 150.987 | -34.135 | *N. nouchali* | 153.037 | -29.076 |
| *N. nouchali* | 48.228 | -18.94 | *N. nouchali* | 30.14 | -12.502 | *N. nouchali* | 152.96 | -26.402 | *N. nouchali* | 150.838 | -33.954 |
| *N. nouchali* | 48.25 | -18.15 | *N. nouchali* | 30.252 | -12.592 | *N. nouchali* | 131.1 | -12.531 | *N. nouchali* | 152.923 | -30.927 |
| *N. nouchali* | 48.258 | -18.28 | *N. nouchali* | 28.874 | -11.197 | *N. nouchali* | 152.758 | -26.913 | *N. nouchali* | 151.767 | -32.804 |
| *N. nouchali* | 48.432 | -18.99 | *N. nouchali* | 31.077 | -10.926 | *N. nouchali* | 131.027 | -12.193 | *N. nouchali* | 150.679 | -34.771 |
| *N. nouchali* | 48.504 | -18.79 | *N. nouchali* | 31.295 | -12.451 | *N. nouchali* | 153.094 | -27.374 | *N. nouchali* | 151.856 | -32.8 |
| *N. nouchali* | 48.686 | -13.59 | *N. nouchali* | 29.22 | -15.356 | *N. nouchali* | 153.355 | -29.439 | *N. nouchali* | 152.826 | -31.567 |
| *N. nouchali* | 48.767 | -19.94 | *N. nouchali* | 23.326 | -16.23 | *N. nouchali* | 153.377 | -28.171 | *N. nouchali* | 151.405 | -33.36 |
| *N. nouchali* | 48.988 | -17.75 | *N. nouchali* | 23.202 | -15.373 | *N. nouchali* | 152.746 | -27.49 | *N. nouchali* | 150.839 | -33.722 |
| *N. nouchali* | 150.813 | -33.88 | *N. nouchali* | 153.469 | -27.575 | *N. nouchali* | 152.795 | -31.025 | *N. nouchali* | 153 | -25.917 |
| *N. nouchali* | 151.911 | -30.53 | *N. nouchali* | 152.072 | -32.758 | *N. nouchali* | 152.849 | -31.035 | *N. nouchali* | 152.676 | -26.19 |
| *N. nouchali* | 150.698 | -33.55 | *N. nouchali* | 152.875 | -31.43 | *N. nouchali* | 152.881 | -30.997 | *N. nouchali* | 153.03 | -31.05 |
| *N. nouchali* | 153.15 | -30.03 | *N. nouchali* | 151.26 | -23.854 | *N. nouchali* | 152.828 | -30.989 | *N. nouchali* | 151.451 | -33.065 |
| *N. nouchali* | 153.207 | -30 | *N. nouchali* | 152.54 | -32.057 | *N. nouchali* | 152.864 | -30.949 | *N. nouchali* | 153.084 | -27.248 |
| *N. nouchali* | 152.432 | -31.86 | *N. nouchali* | 150.85 | -33.807 | *N. nouchali* | 152.767 | -31.093 | *N. nouchali* | 153.217 | -27.733 |
| *N. nouchali* | 152.964 | -30.72 | *N. nouchali* | 153.095 | -27.581 | *N. nouchali* | 152.868 | -31.083 | *N. nouchali* | 153.08 | -27.42 |
| *N. nouchali* | 153.537 | -28.51 | *N. nouchali* | 153.063 | -27.631 | *N. nouchali* | 152.937 | -31.078 | *N. nouchali* | 153.05 | -27.3 |
| *N. nouchali* | 153.007 | -28.65 | *N. nouchali* | 153.52 | -28.8 | *N. nouchali* | 152.975 | -31.022 | *N. nouchali* | 152.83 | -31.13 |
| *N. nouchali* | 153.583 | -28.69 | *N. nouchali* | 151.482 | -33.242 | *N. nouchali* | 151.22 | -33.83 | *N. nouchali* | 153.5 | -28.4 |
| *N. nouchali* | 152.826 | -30.42 | *N. nouchali* | 147.845 | -19.947 | *N. nouchali* | 153.043 | -26.223 | *N. nouchali* | 151.279 | -23.735 |
| *N. nouchali* | 152.906 | -31.47 | *N. nouchali* | 153.055 | -29.671 | *N. nouchali* | 153.418 | -27.582 | *N. odorata* | 117.4 | -35 |
| *N. nouchali* | 153.054 | -29.51 | *N. nouchali* | 151.638 | -24.245 | *N. nouchali* | 153.334 | -29.498 | *N. odorata* | 117.9 | -35 |
| *N. nouchali* | 150.785 | -33.53 | *N. nouchali* | 153.053 | -30.957 | *N. nouchali* | 153.168 | -28.665 | *N. odorata* | 146.03 | -34.25 |
| *N. nouchali* | 153.56 | -28.6 | *N. nouchali* | 153.29 | -28.746 | *N. nouchali* | 143.13 | -29.67 | *N. odorata* | 144.42 | -35.97 |
| *N. nouchali* | 152.812 | -29.8 | *N. nouchali* | 153.079 | -26.603 | *N. nouchali* | 152.97 | -31.18 | *N. odorata* | 150.5 | -33.7 |
| *N. nouchali* | 153.064 | -30.37 | *N. nouchali* | 153.09 | -26.724 | *N. nouchali* | 152.77 | -31.33 | *N. odorata* | 142.235 | -34.216 |
| *N. nouchali* | 153.451 | -27.07 | *N. nouchali* | 152.23 | -25.57 | *N. nouchali* | 152.8 | -30.95 | *N. odorata* | 150.913 | -33.694 |
| *N. nouchali* | 148.203 | -23.53 | *N. nouchali* | 152.926 | -27.607 | *N. nouchali* | 152.62 | -29.18 | *N. odorata* | 115.068 | -33.949 |
| *N. nouchali* | 153.022 | -30.12 | *N. nouchali* | 153.016 | -30.221 | *N. nouchali* | 152.95 | -27.07 | *N. odorata* | 115.938 | -32.026 |
| *N. nouchali* | 153.127 | -27.08 | *N. nouchali* | 151.218 | -33.915 | *N. nouchali* | 152.92 | -31.2 | *N. pubescens* | 132.165 | -13.048 |
| *N. nouchali* | 153.027 | -28.82 | *N. nouchali* | 153.38 | -29.1 | *N. nouchali* | 150.83 | -33.42 | *N. pubescens* | 146.75 | -19.3 |
| *N. nouchali* | 152.831 | -31.46 | *N. nouchali* | 151.201 | -33.498 | *N. nouchali* | 152.95 | -26.3 | *N. pubescens* | 132.219 | -12.571 |
| *N. nouchali* | 153.623 | -28.65 | *N. nouchali* | 152.3 | -25.27 | *N. nouchali* | 153.083 | -26.417 | *N. pubescens* | 131.098 | -12.336 |
| *N. nouchali* | 153.199 | -27.67 | *N. nouchali* | 152.844 | -29.588 | *N. nouchali* | 153.117 | -26.383 | *N. pubescens* | 131.068 | -12.399 |
| *N. nouchali* | 151.471 | -33.17 | *N. nouchali* | 152.904 | -29.571 | *N. nouchali* | 153.083 | -29.6 | *N. pubescens* | 134.936 | -12.197 |
| *N. nouchali* | 153.148 | -29.98 | *N. nouchali* | 152.985 | -29.605 | *N. nouchali* | 146 | -19.5 | *N. pubescens* | 134.989 | -12.44 |
| *N. nouchali* | 153.063 | -29.9 | *N. nouchali* | 153.036 | -29.552 | *N. nouchali* | 152.744 | -25.504 | *N. pubescens* | 135.019 | -12.488 |
| *N. nouchali* | 151.468 | -33.11 | *N. nouchali* | 152.988 | -29.497 | *N. nouchali* | 152.5 | -27.5 | *N. pubescens* | 131.465 | -12.682 |
| *N. nouchali* | 152.81 | -31.19 | *N. nouchali* | 153.051 | -29.398 | *N. nouchali* | 152.75 | -31 | *N. pubescens* | 146.727 | -19.362 |
| *N. nouchali* | 152.449 | -32.09 | *N. nouchali* | 153.123 | -29.465 | *N. nouchali* | 151 | -34 | *N. pubescens* | 146.019 | -17.518 |
| *N. nouchali* | 152.522 | -32.34 | *N. nouchali* | 153.102 | -29.411 | *N. nouchali* | 150.5 | -23 | *N. pubescens* | 133.02 | -12.23 |
| *N. nouchali* | 153 | -31.12 | *N. nouchali* | 153.24 | -29.367 | *N. nouchali* | 153.35 | -28.617 | *N. pubescens* | 132.585 | -12.749 |
| *N. nouchali* | 153.518 | -28.32 | *N. nouchali* | 153.138 | -29.509 | *N. nouchali* | 152.651 | -31.798 | *N. pubescens* | 130.191 | -13.151 |
| *N. nouchali* | 151.797 | -32.58 | *N. nouchali* | 153.028 | -29.728 | *N. nouchali* | 152.87 | -30.77 | *N. pubescens* | 130.287 | -13.129 |
| *N. nouchali* | 152.959 | -26.92 | *N. nouchali* | 153.01 | -29.775 | *N. nouchali* | 152.23 | -32.35 | *N. pubescens* | 130.273 | -13.39 |
| *N. nouchali* | 151.849 | -25.73 | *N. nouchali* | 153.096 | -29.777 | *N. nouchali* | 152.62 | -31.67 | *N. pubescens* | 131.5 | -12.302 |
| *N. nouchali* | 153.452 | -29 | *N. nouchali* | 153.14 | -29.695 | *N. nouchali* | 151.118 | -34.115 | *N. pubescens* | 132.851 | -11.699 |
| *N. nouchali* | 150.091 | -29.77 | *N. nouchali* | 153.131 | -29.643 | *N. nouchali* | 150.468 | -33.198 | *N. pubescens* | 134.968 | -12.482 |
| *N. nouchali* | 152.369 | -32.12 | *N. nouchali* | 153.171 | -29.559 | *N. nouchali* | 153.2 | -30.1 | *N. pubescens* | 129.857 | -13.752 |
| *N. nouchali* | 151.293 | -33.36 | *N. nouchali* | 153.236 | -29.454 | *N. nouchali* | 152.85 | -31.5 | *N. pubescens* | 132.518 | -12.849 |
| *N. pubescens* | 132.201 | -12.62 | *N. pulchella* | -62.471 | 9.953 | *N. pulchella* | -39.192 | -4.236 | *N. pulchella* | -36.725 | -10.604 |
| *N. pubescens* | 130.285 | -13.02 | *N. pulchella* | -62.05 | 7.4 | *N. pulchella* | -39.147 | -6.458 | *N. pulchella* | -36.322 | -10.261 |
| *N. pubescens* | 131.3 | -12.57 | *N. pulchella* | -52.4 | -1.233 | *N. pulchella* | -39.084 | -12.727 | *N. pulchella* | -36.281 | -10.291 |
| *N. pubescens* | 132.283 | -12.58 | *N. pulchella* | -50.76 | -0.11 | *N. pulchella* | -39.08 | -12.53 | *N. pulchella* | -35.855 | -7.051 |
| *N. pubescens* | 132.57 | -12.93 | *N. pulchella* | -42.578 | -11.569 | *N. pulchella* | -39.08 | -7.926 | *N. pulchella* | -35.484 | -7.502 |
| *N. pubescens* | 131.73 | -12.52 | *N. pulchella* | -41.772 | -11.272 | *N. pulchella* | -38.705 | -8.079 | *N. pulchella* | -35.469 | -6.893 |
| *N. pubescens* | 131.57 | -12.62 | *N. pulchella* | -41.759 | -10.348 | *N. pulchella* | -38.599 | -7.131 | *N. pulchella* | -35.468 | -6.998 |
| *N. pubescens* | 132.87 | -12.55 | *N. pulchella* | -41.525 | -13.275 | *N. pulchella* | -38.295 | -6.576 | *N. pulchella* | -35.449 | -7.187 |
| *N. pubescens* | 133.035 | -12.75 | *N. pulchella* | -41.383 | -12.55 | *N. pulchella* | -38.202 | -7.312 | *N. pulchella* | -35.428 | -6.858 |
| *N. pubescens* | 132.87 | -12.47 | *N. pulchella* | -41.371 | -11.407 | *N. pulchella* | -38.169 | -12.765 | *N. pulchella* | -35.421 | -6.929 |
| *N. pubescens* | 131.28 | -12.3 | *N. pulchella* | -41.324 | -12.667 | *N. pulchella* | -38.112 | -7.84 | *N. pulchella* | -35.419 | -6.999 |
| *N. pubescens* | 132.967 | -12.43 | *N. pulchella* | -41.167 | -12.683 | *N. pulchella* | -38.11 | -10.423 | *N. pulchella* | -35.367 | -7.009 |
| *N. pubescens* | 145.917 | -18.47 | *N. pulchella* | -41.086 | -11.48 | *N. pulchella* | -37.735 | -11.034 | *N. pulchella* | -35.316 | -7.184 |
| *N. pubescens* | 145.976 | -18.46 | *N. pulchella* | -41.016 | -11.682 | *N. pulchella* | -37.724 | -7.246 | *N. pulchella* | -35.208 | -6.84 |
| *N. pubescens* | 131.758 | -12.48 | *N. pulchella* | -40.845 | -9.268 | *N. pulchella* | -37.705 | -8.509 | *N. pulchella* | -36.2 | -9.483 |
| *N. pubescens* | 131.62 | -12.31 | *N. pulchella* | -40.834 | -11.692 | *N. pulchella* | -37.652 | -8.598 | *N. pulchella* | -36.667 | -9.417 |
| *N. pubescens* | 132.888 | -12.05 | *N. pulchella* | -40.671 | -9.34 | *N. pulchella* | -37.65 | -10.917 | *N. pulchella* | -36.55 | -9.95 |
| *N. pubescens* | 131.609 | -12.65 | *N. pulchella* | -40.567 | -12.085 | *N. pulchella* | -37.629 | -10.864 | *N. pulchella* | -41.309 | -12.763 |
| *N. pubescens* | 132.984 | -12.26 | *N. pulchella* | -40.552 | -9.318 | *N. pulchella* | -37.616 | -7.137 | *N. pulchella* | -39.151 | -12.193 |
| *N. pubescens* | 131.7 | -12.45 | *N. pulchella* | -40.55 | -11.717 | *N. pulchella* | -37.604 | -8.162 | *N. pulchella* | -38.283 | -12.767 |
| *N. pubescens* | 131.637 | -12.72 | *N. pulchella* | -40.492 | -6.43 | *N. pulchella* | -37.6 | -11.8 | *N. pulchella* | -38.476 | -12.646 |
| *N. pubescens* | 134.662 | -12.25 | *N. pulchella* | -40.467 | -11.85 | *N. pulchella* | -37.58 | -8.05 | *N. pulchella* | -40.018 | -11.323 |
| *N. pubescens* | 134.467 | -12.14 | *N. pulchella* | -39.984 | -6.771 | *N. pulchella* | -37.541 | -10.667 | *N. pulchella* | -38.963 | -12.428 |
| *N. pubescens* | 132.066 | -12.4 | *N. pulchella* | -39.94 | -3.764 | *N. pulchella* | -37.433 | -11.041 | *N. pulchella* | -37.716 | -12.053 |
| *N. pubescens* | 132.419 | -12.58 | *N. pulchella* | -39.85 | -8.268 | *N. pulchella* | -37.42 | -10.218 | *N. pulchella* | -38.083 | -11.933 |
| *N. pubescens* | 132.169 | -12.55 | *N. pulchella* | -39.722 | -14.167 | *N. pulchella* | -37.39 | -10.55 | *N. pulchella* | -38.982 | -10.586 |
| *N. pubescens* | 132.152 | -12.41 | *N. pulchella* | -39.583 | -7.891 | *N. pulchella* | -37.352 | -10.411 | *N. pulchella* | -39.088 | -12.243 |
| *N. pubescens* | 132.902 | -12.16 | *N. pulchella* | -39.517 | -12.848 | *N. pulchella* | -37.339 | -6.235 | *N. pulchella* | -40.227 | -12.489 |
| *N. pubescens* | 132.552 | -12.81 | *N. pulchella* | -39.493 | -3.811 | *N. pulchella* | -37.312 | -6.411 | *N. pulchella* | -39.867 | -10.65 |
| *N. pubescens* | 131.286 | -12.4 | *N. pulchella* | -39.49 | -4.9 | *N. pulchella* | -37.31 | -10.998 | *N. pulchella* | -40.557 | -11.188 |
| *N. pubescens* | 136.297 | -12.44 | *N. pulchella* | -39.446 | -12.961 | *N. pulchella* | -37.251 | -10.131 | *N. pulchella* | -40.656 | -9.562 |
| *N. pubescens* | 132.52 | -12.89 | *N. pulchella* | -39.389 | -4.626 | *N. pulchella* | -37.229 | -7.877 | *N. pulchella* | -38.283 | -12.517 |
| *N. pulchella* | -80.45 | -0.917 | *N. pulchella* | -39.371 | -3.723 | *N. pulchella* | -37.206 | -11.015 | *N. pulchella* | -40.608 | -11.328 |
| *N. pulchella* | -79.95 | -2.717 | *N. pulchella* | -39.333 | -6.791 | *N. pulchella* | -37.193 | -10.492 | *N. pulchella* | -41.733 | -12.488 |
| *N. pulchella* | -79.717 | -2.3 | *N. pulchella* | -39.33 | -8.307 | *N. pulchella* | -37.169 | -8.63 | *N. pulchella* | -40.261 | -10.705 |
| *N. pulchella* | -79.667 | -2.433 | *N. pulchella* | -39.309 | -8.362 | *N. pulchella* | -37.143 | -10.843 | *N. pulchella* | -38.428 | -12.944 |
| *N. pulchella* | -79.633 | -2.3 | *N. pulchella* | -39.308 | -6.502 | *N. pulchella* | -37.103 | -10.921 | *N. pulchella* | -38.967 | -11.933 |
| *N. pulchella* | -75.572 | 6.27 | *N. pulchella* | -39.298 | -3.616 | *N. pulchella* | -37.054 | -10.789 | *N. pulchella* | -43.507 | -12.364 |
| *N. pulchella* | -75 | 9 | *N. pulchella* | -39.283 | -6.43 | *N. pulchella* | -36.967 | -10.114 | *N. pulchella* | -41.808 | -9.873 |
| *N. pulchella* | -74.448 | 6.596 | *N. pulchella* | -39.218 | -6.501 | *N. pulchella* | -36.94 | -10.593 | *N. pulchella* | -39.409 | -12.144 |
| *N. pulchella* | -74.376 | -9.164 | *N. pulchella* | -39.204 | -8.062 | *N. pulchella* | -36.799 | -7.15 | *N. pulchella* | -39.092 | -11.981 |
| *N. pulchella* | -41.233 | -12.85 | *N. rudgeana* | -36.461 | -10.396 | *N. violacea* | 148.772 | -21.978 | *N. violacea* | 122.261 | -17.78 |
| *N. pulchella* | -38.301 | -7.249 | *N. rudgeana* | -47.876 | -3.265 | *N. violacea* | 148.6 | -21.574 | *N. violacea* | 137.683 | -17.75 |
| *N. pulchella* | -35.95 | -8.317 | *N. rudgeana* | -35.285 | -7.193 | *N. violacea* | 149.2 | -21.4 | *N. violacea* | 146.109 | -17.69 |
| *N. pulchella* | -39.117 | -8.283 | *N. rudgeana* | -50.167 | -1 | *N. violacea* | 145.88 | -20.941 | *N. violacea* | 141.089 | -17.644 |
| *N. pulchella* | -41.661 | -22.19 | *N. rudgeana* | -43.083 | -11.052 | *N. violacea* | 147.25 | -20.25 | *N. violacea* | 139.186 | -17.593 |
| *N. pulchella* | -41.472 | -22.11 | *N. rudgeana* | -35.533 | -7.213 | *N. violacea* | 145.97 | -20.246 | *N. violacea* | 126.175 | -17.549 |
| *N. rudgeana* | -54.9 | -9.367 | *N. rudgeana* | -75.65 | 1.18 | *N. violacea* | 145.931 | -19.981 | *N. violacea* | 130.519 | -17.547 |
| *N. rudgeana* | -59.017 | -16.32 | *N. rudgeana* | -59.59 | 5.363 | *N. violacea* | 147.934 | -19.951 | *N. violacea* | 126.111 | -17.525 |
| *N. rudgeana* | -59.483 | 5.167 | *N. rudgeana* | -59.578 | -2.649 | *N. violacea* | 146.617 | -19.867 | *N. violacea* | 123.727 | -17.454 |
| *N. rudgeana* | -58.25 | 6.5 | *N. rudgeana* | -59.53 | 3.91 | *N. violacea* | 147.17 | -19.83 | *N. violacea* | 127.611 | -17.428 |
| *N. rudgeana* | -38.283 | -12.6 | *N. rudgeana* | -44.67 | -4.32 | *N. violacea* | 146.835 | -19.759 | *N. violacea* | 130.836 | -17.414 |
| *N. rudgeana* | -38.083 | -11.93 | *N. rudgeana* | -44.273 | -2.596 | *N. violacea* | 145.95 | -19.75 | *N. violacea* | 124.55 | -17.367 |
| *N. rudgeana* | -59.85 | 7.833 | *N. rudgeana* | -35.232 | -6.144 | *N. violacea* | 147.4 | -19.59 | *N. violacea* | 124.887 | -17.336 |
| *N. rudgeana* | -59.53 | 5.376 | *N. rudgeana* | -37.6 | -11.8 | *N. violacea* | 146.83 | -19.5 | *N. violacea* | 138.233 | -17.333 |
| *N. rudgeana* | -41.391 | -13 | *N. rudgeana* | -63.027 | -7.506 | *N. violacea* | 146.726 | -19.457 | *N. violacea* | 124.62 | -17.33 |
| *N. rudgeana* | -61.167 | 9.25 | *N. rudgeana* | -40.505 | -9.438 | *N. violacea* | 147.061 | -19.347 | *N. violacea* | 125.3 | -17.317 |
| *N. rudgeana* | -52.033 | -0.65 | *N. rudgeana* | -39.028 | -16.279 | *N. violacea* | 146.858 | -19.336 | *N. violacea* | 125.85 | -17.3 |
| *N. rudgeana* | -51.5 | -2 | *N. rudgeana* | -38.543 | -3.718 | *N. violacea* | 146.751 | -19.248 | *N. violacea* | 123.85 | -17.27 |
| *N. rudgeana* | -50.397 | -6.372 | *N. rudgeana* | -45.098 | -7.214 | *N. violacea* | 146.632 | -19.181 | *N. violacea* | 126.9 | -17.27 |
| *N. rudgeana* | -35.233 | -7.096 | *N. rudgeana* | -45.336 | -15.485 | *N. violacea* | 138.567 | -19.117 | *N. violacea* | 126.43 | -17.2 |
| *N. rudgeana* | -72.75 | 9.6 | *N. rudgeana* | -48.298 | -1.379 | *N. violacea* | 146.45 | -19.109 | *N. violacea* | 126.25 | -17.2 |
| *N. rudgeana* | -63.37 | -7.961 | *N. rudgeana* | -46.669 | -1.19 | *N. violacea* | 138.726 | -19.019 | *N. violacea* | 128.6 | -17.183 |
| *N. rudgeana* | -63 | -8 | *N. rudgeana* | -48.155 | -1.37 | *N. violacea* | 141 | -19 | *N. violacea* | 144.5 | -17.181 |
| *N. rudgeana* | -54.75 | 2.51 | *N. rudgeana* | -54.789 | -2.445 | *N. violacea* | 138.511 | -18.775 | *N. violacea* | 125.28 | -17.17 |
| *N. rudgeana* | -62.283 | 5.95 | *N. rudgeana* | -35.082 | -7.791 | *N. violacea* | 138.42 | -18.75 | *N. violacea* | 125.33 | -17.17 |
| *N. rudgeana* | -62.25 | 5.9 | *N. rudgeana* | -35.084 | -8.109 | *N. violacea* | 146.133 | -18.717 | *N. violacea* | 128.735 | -17.165 |
| *N. rudgeana* | -55.759 | -1.165 | *N. rudgeana* | -41.324 | -21.754 | *N. violacea* | 138.493 | -18.707 | *N. violacea* | 137.769 | -17.164 |
| *N. rudgeana* | -55.33 | 5.25 | *N. violacea* | 152.857 | -30.999 | *N. violacea* | 138.49 | -18.617 | *N. violacea* | 145.2 | -17.142 |
| *N. rudgeana* | -52.33 | 4.93 | *N. violacea* | 152.952 | -30.68 | *N. violacea* | 138.585 | -18.582 | *N. violacea* | 125.182 | -17.107 |
| *N. rudgeana* | -48.52 | -25.7 | *N. violacea* | 153.007 | -28.646 | *N. violacea* | 124.717 | -18.55 | *N. violacea* | 139.085 | -17.082 |
| *N. rudgeana* | -48.383 | -1.146 | *N. violacea* | 152.973 | -27.474 | *N. violacea* | 137.952 | -18.514 | *N. violacea* | 144.284 | -17.036 |
| *N. rudgeana* | -48.245 | -1.321 | *N. violacea* | 152.55 | -26.933 | *N. violacea* | 144.25 | -18.383 | *N. violacea* | 145.5 | -16.983 |
| *N. rudgeana* | -61.167 | 5 | *N. violacea* | 152.93 | -26.388 | *N. violacea* | 125.58 | -18.292 | *N. violacea* | 122.75 | -16.967 |
| *N. rudgeana* | -58.01 | 6.53 | *N. violacea* | 151.267 | -24.217 | *N. violacea* | 124.498 | -18.275 | *N. violacea* | 122.667 | -16.967 |
| *N. rudgeana* | -58.117 | 5.367 | *N. violacea* | 151.183 | -24 | *N. violacea* | 137.068 | -18.115 | *N. violacea* | 125.58 | -16.95 |
| *N. rudgeana* | -52.38 | 4.43 | *N. violacea* | 151.083 | -23.8 | *N. violacea* | 140.56 | -18.023 | *N. violacea* | 125.779 | -16.927 |
| *N. rudgeana* | -57.583 | 5.6 | *N. violacea* | 150.617 | -23.217 | *N. violacea* | 145.15 | -17.92 | *N. violacea* | 125.07 | -16.92 |
| *N. rudgeana* | -54.333 | 5.667 | *N. violacea* | 150.667 | -23.217 | *N. violacea* | 140.17 | -17.92 | *N. violacea* | 145.4 | -16.9 |
| *N. rudgeana* | -60 | 5.083 | *N. violacea* | 150.587 | -23.105 | *N. violacea* | 138.58 | -17.88 | *N. violacea* | 137.811 | -16.884 |
| *N. rudgeana* | -59.98 | 8.16 | *N. violacea* | 150.273 | -22.719 | *N. violacea* | 139.55 | -17.817 | *N. violacea* | 130.201 | -16.882 |
| *N. rudgeana* | -60.43 | 1.644 | *N. violacea* | 150.22 | -22.426 | *N. violacea* | 130.591 | -17.785 | *N. violacea* | 144.876 | -16.873 |
| *N. violacea* | 145.368 | -16.87 | *N. violacea* | 130.486 | -16.064 | *N. violacea* | 132.651 | -15.531 | *N. violacea* | 126.733 | -15.05 |
| *N. violacea* | 137.251 | -16.87 | *N. violacea* | 130.41 | -16.062 | *N. violacea* | 129.159 | -15.529 | *N. violacea* | 145.143 | -15.007 |
| *N. violacea* | 145.367 | -16.82 | *N. violacea* | 128.735 | -16.031 | *N. violacea* | 135.718 | -15.507 | *N. violacea* | 144.604 | -15.003 |
| *N. violacea* | 136.55 | -16.8 | *N. violacea* | 130.463 | -16.019 | *N. violacea* | 145.224 | -15.507 | *N. violacea* | 130.057 | -15.002 |
| *N. violacea* | 131.219 | -16.8 | *N. violacea* | 127.917 | -16 | *N. violacea* | 144.484 | -15.502 | *N. violacea* | 133.201 | -14.949 |
| *N. violacea* | 137.101 | -16.79 | *N. violacea* | 143 | -16 | *N. violacea* | 135.4 | -15.5 | *N. violacea* | 133.139 | -14.941 |
| *N. violacea* | 125.833 | -16.78 | *N. violacea* | 135.833 | -16 | *N. violacea* | 145.17 | -15.5 | *N. violacea* | 144.2 | -14.917 |
| *N. violacea* | 145.33 | -16.75 | *N. violacea* | 136.268 | -15.999 | *N. violacea* | 144.35 | -15.5 | *N. violacea* | 133.435 | -14.915 |
| *N. violacea* | 136.468 | -16.75 | *N. violacea* | 128.717 | -15.967 | *N. violacea* | 145.007 | -15.49 | *N. violacea* | 133.087 | -14.911 |
| *N. violacea* | 135.633 | -16.72 | *N. violacea* | 127.441 | -15.96 | *N. violacea* | 128.267 | -15.483 | *N. violacea* | 128.661 | -14.902 |
| *N. violacea* | 136.231 | -16.71 | *N. violacea* | 135.757 | -15.955 | *N. violacea* | 128.5 | -15.483 | *N. violacea* | 126.201 | -14.9 |
| *N. violacea* | 135.432 | -16.69 | *N. violacea* | 129.923 | -15.883 | *N. violacea* | 141.758 | -15.475 | *N. violacea* | 142.83 | -14.9 |
| *N. violacea* | 145.326 | -16.69 | *N. violacea* | 134.567 | -15.867 | *N. violacea* | 125.1 | -15.47 | *N. violacea* | 133.038 | -14.887 |
| *N. violacea* | 135.67 | -16.68 | *N. violacea* | 136.483 | -15.867 | *N. violacea* | 129.345 | -15.463 | *N. violacea* | 126.831 | -14.847 |
| *N. violacea* | 125.492 | -16.65 | *N. violacea* | 127.361 | -15.864 | *N. violacea* | 141.658 | -15.458 | *N. violacea* | 125.696 | -14.829 |
| *N. violacea* | 129.8 | -16.6 | *N. violacea* | 127.557 | -15.838 | *N. violacea* | 124.667 | -15.433 | *N. violacea* | 126.917 | -14.817 |
| *N. violacea* | 144.85 | -16.6 | *N. violacea* | 127.648 | -15.838 | *N. violacea* | 126.3 | -15.433 | *N. violacea* | 143.376 | -14.807 |
| *N. violacea* | 143.566 | -16.57 | *N. violacea* | 135.426 | -15.829 | *N. violacea* | 145.17 | -15.43 | *N. violacea* | 126.222 | -14.805 |
| *N. violacea* | 129.686 | -16.55 | *N. violacea* | 129.33 | -15.796 | *N. violacea* | 145.043 | -15.419 | *N. violacea* | 126.493 | -14.803 |
| *N. violacea* | 125.217 | -16.53 | *N. violacea* | 128.767 | -15.783 | *N. violacea* | 130.875 | -15.405 | *N. violacea* | 127.083 | -14.783 |
| *N. violacea* | 145.123 | -16.53 | *N. violacea* | 129.036 | -15.781 | *N. violacea* | 141.712 | -15.405 | *N. violacea* | 134.888 | -14.783 |
| *N. violacea* | 129.992 | -16.51 | *N. violacea* | 130.036 | -15.764 | *N. violacea* | 135.356 | -15.372 | *N. violacea* | 131.123 | -14.754 |
| *N. violacea* | 126.4 | -16.5 | *N. violacea* | 144.266 | -15.751 | *N. violacea* | 134.831 | -15.365 | *N. violacea* | 125.65 | -14.75 |
| *N. violacea* | 145.453 | -16.5 | *N. violacea* | 145 | -15.75 | *N. violacea* | 129.692 | -15.361 | *N. violacea* | 130.802 | -14.731 |
| *N. violacea* | 125.351 | -16.48 | *N. violacea* | 137.068 | -15.749 | *N. violacea* | 135.322 | -15.295 | *N. violacea* | 144.059 | -14.707 |
| *N. violacea* | 144.872 | -16.47 | *N. violacea* | 130.56 | -15.734 | *N. violacea* | 127.2 | -15.283 | *N. violacea* | 134.461 | -14.703 |
| *N. violacea* | 136.167 | -16.45 | *N. violacea* | 143.565 | -15.692 | *N. violacea* | 145.33 | -15.27 | *N. violacea* | 141.783 | -14.7 |
| *N. violacea* | 126.8 | -16.43 | *N. violacea* | 145.119 | -15.686 | *N. violacea* | 126.717 | -15.267 | *N. violacea* | 142.301 | -14.699 |
| *N. violacea* | 127.186 | -16.41 | *N. violacea* | 130.48 | -15.67 | *N. violacea* | 143.917 | -15.217 | *N. violacea* | 143.993 | -14.697 |
| *N. violacea* | 135.7 | -16.37 | *N. violacea* | 130.383 | -15.659 | *N. violacea* | 125.728 | -15.21 | *N. violacea* | 143.839 | -14.667 |
| *N. violacea* | 124.971 | -16.35 | *N. violacea* | 136.383 | -15.65 | *N. violacea* | 126.161 | -15.195 | *N. violacea* | 125.75 | -14.667 |
| *N. violacea* | 137.783 | -16.33 | *N. violacea* | 135.718 | -15.635 | *N. violacea* | 144.359 | -15.19 | *N. violacea* | 129.863 | -14.591 |
| *N. violacea* | 145.41 | -16.33 | *N. violacea* | 130.452 | -15.631 | *N. violacea* | 127.85 | -15.133 | *N. violacea* | 132.874 | -14.538 |
| *N. violacea* | 144.718 | -16.27 | *N. violacea* | 128.085 | -15.629 | *N. violacea* | 126.12 | -15.13 | *N. violacea* | 129.902 | -14.531 |
| *N. violacea* | 135.659 | -16.26 | *N. violacea* | 135.462 | -15.626 | *N. violacea* | 129.895 | -15.119 | *N. violacea* | 132.77 | -14.52 |
| *N. violacea* | 144.761 | -16.17 | *N. violacea* | 129.058 | -15.62 | *N. violacea* | 130.388 | -15.117 | *N. violacea* | 143.808 | -14.504 |
| *N. violacea* | 127.753 | -16.15 | *N. violacea* | 141.733 | -15.617 | *N. violacea* | 131.618 | -15.115 | *N. violacea* | 134.229 | -14.5 |
| *N. violacea* | 129.796 | -16.12 | *N. violacea* | 130.35 | -15.583 | *N. violacea* | 145.133 | -15.1 | *N. violacea* | 132.22 | -14.5 |
| *N. violacea* | 130.431 | -16.12 | *N. violacea* | 144.441 | -15.573 | *N. violacea* | 133.201 | -15.099 | *N. violacea* | 132.835 | -14.499 |
| *N. violacea* | 136.685 | -16.12 | *N. violacea* | 128.317 | -15.567 | *N. violacea* | 130.541 | -15.072 | *N. violacea* | 127.654 | -14.494 |
| *N. violacea* | 144.777 | -16.11 | *N. violacea* | 126.217 | -15.567 | *N. violacea* | 130.475 | -15.068 | *N. violacea* | 134.233 | -14.445 |
| *N. violacea* | 131.694 | -14.39 | *N. violacea* | 129.87 | -13.717 | *N. violacea* | 134.933 | -13.014 | *N. violacea* | 142.076 | -12.707 |
| *N. violacea* | 132.253 | -14.39 | *N. violacea* | 131.207 | -13.709 | *N. violacea* | 130.452 | -13.014 | *N. violacea* | 132.564 | -12.699 |
| *N. violacea* | 130.186 | -14.33 | *N. violacea* | 143.504 | -13.679 | *N. violacea* | 134.5 | -13 | *N. violacea* | 132.935 | -12.682 |
| *N. violacea* | 132.47 | -14.33 | *N. violacea* | 130.461 | -13.673 | *N. violacea* | 131 | -13 | *N. violacea* | 131.465 | -12.682 |
| *N. violacea* | 129.913 | -14.33 | *N. violacea* | 134.301 | -13.665 | *N. violacea* | 130.905 | -12.981 | *N. violacea* | 135.221 | -12.681 |
| *N. violacea* | 143.741 | -14.32 | *N. violacea* | 130.39 | -13.625 | *N. violacea* | 130.413 | -12.977 | *N. violacea* | 142.115 | -12.677 |
| *N. violacea* | 126.633 | -14.3 | *N. violacea* | 130.489 | -13.601 | *N. violacea* | 130.291 | -12.949 | *N. violacea* | 132.615 | -12.675 |
| *N. violacea* | 132.415 | -14.3 | *N. violacea* | 130.057 | -13.572 | *N. violacea* | 132.001 | -12.949 | *N. violacea* | 132.818 | -12.665 |
| *N. violacea* | 129.859 | -14.29 | *N. violacea* | 142.691 | -13.545 | *N. violacea* | 132.551 | -12.942 | *N. violacea* | 135.385 | -12.664 |
| *N. violacea* | 143.704 | -14.27 | *N. violacea* | 130.868 | -13.532 | *N. violacea* | 131.082 | -12.941 | *N. violacea* | 142.671 | -12.658 |
| *N. violacea* | 129.916 | -14.25 | *N. violacea* | 132.017 | -13.483 | *N. violacea* | 130.56 | -12.932 | *N. violacea* | 132.574 | -12.651 |
| *N. violacea* | 136.712 | -14.22 | *N. violacea* | 134.151 | -13.482 | *N. violacea* | 132.411 | -12.92 | *N. violacea* | 131.709 | -12.649 |
| *N. violacea* | 134.368 | -14.2 | *N. violacea* | 130.25 | -13.467 | *N. violacea* | 135.401 | -12.915 | *N. violacea* | 135.318 | -12.649 |
| *N. violacea* | 132.191 | -14.18 | *N. violacea* | 131.167 | -13.467 | *N. violacea* | 135.459 | -12.903 | *N. violacea* | 141.865 | -12.637 |
| *N. violacea* | 130.689 | -14.18 | *N. violacea* | 142.7 | -13.451 | *N. violacea* | 132.672 | -12.902 | *N. violacea* | 132.885 | -12.632 |
| *N. violacea* | 129.789 | -14.17 | *N. violacea* | 130.464 | -13.398 | *N. violacea* | 130.336 | -12.9 | *N. violacea* | 135.201 | -12.614 |
| *N. violacea* | 133.25 | -14.17 | *N. violacea* | 136.138 | -13.391 | *N. violacea* | 131.218 | -12.899 | *N. violacea* | 132.293 | -12.6 |
| *N. violacea* | 131.078 | -14.15 | *N. violacea* | 141.793 | -13.373 | *N. violacea* | 131.641 | -12.882 | *N. violacea* | 131.709 | -12.599 |
| *N. violacea* | 130.038 | -14.15 | *N. violacea* | 141.733 | -13.35 | *N. violacea* | 130.522 | -12.873 | *N. violacea* | 135.419 | -12.597 |
| *N. violacea* | 129.863 | -14.15 | *N. violacea* | 130.237 | -13.349 | *N. violacea* | 135.652 | -12.864 | *N. violacea* | 131.021 | -12.595 |
| *N. violacea* | 129.891 | -14.1 | *N. violacea* | 131.136 | -13.331 | *N. violacea* | 130.411 | -12.85 | *N. violacea* | 132.369 | -12.595 |
| *N. violacea* | 134.102 | -14.05 | *N. violacea* | 130.407 | -13.292 | *N. violacea* | 130.588 | -12.849 | *N. violacea* | 134.949 | -12.589 |
| *N. violacea* | 127.3 | -14.03 | *N. violacea* | 130.487 | -13.273 | *N. violacea* | 130.648 | -12.849 | *N. violacea* | 131.166 | -12.587 |
| *N. violacea* | 126.757 | -14.03 | *N. violacea* | 141.893 | -13.257 | *N. violacea* | 130.985 | -12.849 | *N. violacea* | 132.867 | -12.583 |
| *N. violacea* | 126.933 | -14.02 | *N. violacea* | 130.366 | -13.225 | *N. violacea* | 132.024 | -12.824 | *N. violacea* | 132.22 | -12.58 |
| *N. violacea* | 143.191 | -14.01 | *N. violacea* | 130.464 | -13.224 | *N. violacea* | 132.75 | -12.82 | *N. violacea* | 132.566 | -12.577 |
| *N. violacea* | 143.55 | -13.99 | *N. violacea* | 130.516 | -13.223 | *N. violacea* | 131.667 | -12.8 | *N. violacea* | 135.011 | -12.565 |
| *N. violacea* | 132.218 | -13.99 | *N. violacea* | 130.267 | -13.2 | *N. violacea* | 132.579 | -12.797 | *N. violacea* | 131.305 | -12.565 |
| *N. violacea* | 141.658 | -13.98 | *N. violacea* | 136.201 | -13.199 | *N. violacea* | 141.872 | -12.762 | *N. violacea* | 132.286 | -12.551 |
| *N. violacea* | 136.469 | -13.96 | *N. violacea* | 130.413 | -13.176 | *N. violacea* | 132.633 | -12.751 | *N. violacea* | 135.067 | -12.537 |
| *N. violacea* | 130.835 | -13.93 | *N. violacea* | 130.463 | -13.175 | *N. violacea* | 132.585 | -12.749 | *N. violacea* | 131.235 | -12.527 |
| *N. violacea* | 129.888 | -13.88 | *N. violacea* | 130.259 | -13.153 | *N. violacea* | 132.818 | -12.749 | *N. violacea* | 132.035 | -12.523 |
| *N. violacea* | 143.3 | -13.87 | *N. violacea* | 130.336 | -13.152 | *N. violacea* | 142.251 | -12.748 | *N. violacea* | 132.214 | -12.518 |
| *N. violacea* | 136.492 | -13.84 | *N. violacea* | 130.191 | -13.151 | *N. violacea* | 132.336 | -12.737 | *N. violacea* | 131.085 | -12.515 |
| *N. violacea* | 129.851 | -13.83 | *N. violacea* | 130.567 | -13.1 | *N. violacea* | 132.736 | -12.724 | *N. violacea* | 132.144 | -11.154 |
| *N. violacea* | 134.185 | -13.78 | *N. violacea* | 142.93 | -13.1 | *N. violacea* | 132.49 | -12.724 | *N. violacea* | 142.367 | -11.15 |
| *N. violacea* | 133.152 | -13.77 | *N. violacea* | 132.16 | -13.081 | *N. violacea* | 131.636 | -12.722 | *N. violacea* | 142.483 | -10.917 |
| *N. violacea* | 130.633 | -13.77 | *N. violacea* | 130.287 | -13.074 | *N. violacea* | 135.592 | -12.722 | *N. violacea* | 131.938 | -12.4 |
| *N. violacea* | 130.41 | -13.75 | *N. violacea* | 134.912 | -13.068 | *N. violacea* | 131.016 | -12.72 | *N. violacea* | 131.269 | -12.4 |
| *N. violacea* | 130.463 | -13.75 | *N. violacea* | 130.34 | -13.049 | *N. violacea* | 132.668 | -12.715 | *N. violacea* | 132.944 | -12.4 |
| *N. violacea* | 130.718 | -13.73 | *N. violacea* | 130.289 | -13.015 | *N. violacea* | 142.514 | -12.71 | *N. violacea* | 131.317 | -12.38 |
| *N. violacea* | 131.586 | -12.47 | *N. violacea* | 132.491 | -12.245 | *N. violacea* | 142.126 | -10.124 | *N. violacea* | 134.918 | -12.37 |
| *N. violacea* | 133.333 | -12.47 | *N. violacea* | 134.985 | -12.232 | *N. violacea* | 134.985 | -12.31 | *N. violacea* | 135.046 | -12.36 |
| *N. violacea* | 132.868 | -12.47 | *N. violacea* | 134.861 | -12.23 | *N. violacea* | 133.056 | -12.3 | *N. violacea* | 131.654 | -12.36 |
| *N. violacea* | 131.687 | -12.46 | *N. violacea* | 134.617 | -12.222 | *N. violacea* | 131.971 | -12.3 | *N. violacea* | 132.763 | -12.36 |
| *N. violacea* | 132.815 | -12.46 | *N. violacea* | 136.8 | -12.22 | *N. violacea* | 132.128 | -12.3 | *N. violacea* | 131.947 | -12.35 |
| *N. violacea* | 130.97 | -12.45 | *N. violacea* | 134.537 | -12.218 | *N. violacea* | 131.27 | -12.3 | *N. violacea* | 132.835 | -12.35 |
| *N. violacea* | 132.917 | -12.45 | *N. violacea* | 134.964 | -12.186 | *N. violacea* | 133.185 | -12.3 | *N. violacea* | 136.397 | -12.35 |
| *N. violacea* | 132.089 | -12.45 | *N. violacea* | 132.989 | -12.18 | *N. violacea* | 134.081 | -12.29 | *N. violacea* | 131.2 | -12.33 |
| *N. violacea* | 134.7 | -12.43 | *N. violacea* | 136.78 | -12.179 | *N. violacea* | 135.014 | -12.28 | *N. violacea* | 132.341 | -12.32 |
| *N. violacea* | 131.061 | -12.43 | *N. violacea* | 132.512 | -12.173 | *N. violacea* | 134.723 | -12.28 | *N. violacea* | 131.538 | -12.32 |
| *N. violacea* | 134.982 | -12.42 | *N. violacea* | 132.847 | -12.17 | *N. violacea* | 132.317 | -12.27 | *N. violacea* | 134.917 | -12.32 |
| *N. violacea* | 131.994 | -12.42 | *N. violacea* | 134.494 | -12.145 | *N. violacea* | 134.917 | -12.27 | *N. violacea* | 134.283 | -12.1 |
| *N. violacea* | 131.878 | -12.42 | *N. violacea* | 134.441 | -12.14 | *N. violacea* | 133.019 | -12.26 | *N. violacea* | 132.886 | -12.081 |
| *N. violacea* | 132.885 | -12.41 | *N. violacea* | 134.646 | -12.125 | *N. violacea* | 132.885 | -12.514 | *N. violacea* | 134.69 | -12.07 |
| *N. violacea* | 132.915 | -11.723 | *N. violacea* | 131.167 | -11.583 | *N. violacea* | 135.11 | -12.512 | *N. violacea* | 135.701 | -11.949 |
| *N. violacea* | 132.864 | -11.7 | *N. violacea* | 132.918 | -11.532 | *N. violacea* | 132.467 | -12.5 | *N. violacea* | 132.884 | -11.852 |
| *N. violacea* | 133.385 | -11.614 | *N. violacea* | 130.9 | -11.468 | *N. violacea* | 135.918 | -12.499 | *N. violacea* | 132.737 | -11.832 |
| *N. violacea* | 130.236 | -11.464 | *N. violacea* | 134.635 | -12.068 | *N. violacea* | 135.022 | -12.488 | *N. violacea* | 132.552 | -11.385 |
| *N. violacea* | 132.117 | -11.439 | *N. violacea* | 132.864 | -12.022 | *N. violacea* | 131.758 | -12.482 | *N. violacea* | 132.291 | -11.354 |
| *N. violacea* | 130.512 | -11.413 | *N. violacea* | 134.667 | -12.02 | *N. violacea* | 134.964 | -12.475 | *N. violacea* | 132.401 | -11.332 |
| *N. violacea* | 131.985 | -11.315 | *N. violacea* | 131.165 | -11.283 | *N. violacea* | 132.09 | -11.241 |  |  |  |
