# Supplementary material for: Climatic niche evolution and niche conservatism of Nymphaea species in Africa, South America, and Australia
Source: BMC Plant Biol. 2024 May 30;24:476. doi: 10.1186/s12870-024-05141-1 (PMC11137912; doi:10.1186/s12870-024-05141-1)
Supplement: Supplementary file 5 — Supplementary Material 5 [file 12870_2024_5141_MOESM5_ESM.docx]

**Table S3.** The 19 bioclimatic variables obtained from WorldClim v1.4

| **Bioclimatic variable** | **Code** |
| --- | --- |
| Annual mean temperature | **bio1** |
| Mean diurnal range D Mean of monthly (max temp -min temp) | **bio2** |
| Isothermality (bio2/bio7) (*100) | **bio3** |
| Temperature seasonality (standard deviation * 100) | **bio4** |
| Maximum temperature of warmest month | **bio5** |
| Minimum temperature of coldest month | **bio6** |
| Temperature annual range (Bio5–Bio6) | **bio7** |
| Mean temperature of wettest quarter | **bio8** |
| Mean temperature of the driest quarter | **bio9** |
| Mean temperature of warmest quarter | **bio10** |
| Mean temperature of coldest quarter | **bio11** |
| Annual precipitation | **bio12** |
| Precipitation of wettest month | **bio13** |
| Precipitation of driest month | **bio14** |
| Precipitation seasonality (coefficient of variation) | **bio15** |
| Precipitation of wettest quarter | **bio16** |
| Precipitation of driest quarter | **bio17** |
| Precipitation of warmest quarter | **bio18** |
| Precipitation of coldest quarter | **bio19** |
