# Supplementary material for: Climatic niche evolution and niche conservatism of Nymphaea species in Africa, South America, and Australia
Source: BMC Plant Biol. 2024 May 30;24:476. doi: 10.1186/s12870-024-05141-1 (PMC11137912; doi:10.1186/s12870-024-05141-1)
Supplement: Supplementary file 6 — Supplementary Material 6 [file 12870_2024_5141_MOESM6_ESM.docx]

**Table S4.** Accession numbers and species used for the analysis of phylogenetic niche evolution

| **Species** | **ITS** | ***trnT***–***trnF*** |
| --- | --- | --- |
| *N. alba* | FM242148 | AM422066 |
| *N. amazonum* | FM242149 | AM422026 |
| *N. ampla* | FJ026604 | AM422044 |
| *N. atrans* | FJ026554 | AM422055 |
| *N. caerulea* | AY707897 | FR717554 |
| *N. carpentariae* | FJ026556 | FJ026519 |
| *N. elleniae* | FJ026562 | AM422057 |
| *N. georginae* | FJ026563 | FJ026523 |
| *N. gigantea* | FJ026564 | AM422058 |
| *N. hastifolia* | FJ026568 | AM422060 |
| *N. heudelotii* | FJ026603 | AM422052 |
| *N. immutabilis* | FJ026573 | AM422061 |
| *N. jamesoniana* | FM242152 | AM422032 |
| *N. lingulata* | HG518070 | AM422031 |
| *N. lotus* | MK452746 | MK452761 |
| *N. macrosperma* | FJ026577 | AM489715 |
| *N. mexicana* | AY771814 | AM422072 |
| *N. micrantha* | FR717592 | FR717561 |
| *N. nouchali* | FJ597740 | MW809738 |
| *N. odorata* | EF526395 | AM422073 |
| *N. pubescens* | FJ198406 | AM422043 |
| *N. pulchella* | FR717596 | FR717563 |
| *N. rudgeana* | EU428068 | AM422038 |
| *N. violacea* | FJ026590 | AM422065 |
| *N. novogranatensis* | FM242154 | AM422034 |
| *N. oxypetala* | FM242150 | AM422035 |
| *B. longifolia* | FM242140 | AM422019 |
