# Supplementary figures and images for: Climatic niche evolution and niche conservatism of Nymphaea species in Africa, South America, and Australia
Source: BMC Plant Biol. 2024 May 30;24:476. doi: 10.1186/s12870-024-05141-1 (PMC11137912; doi:10.1186/s12870-024-05141-1)

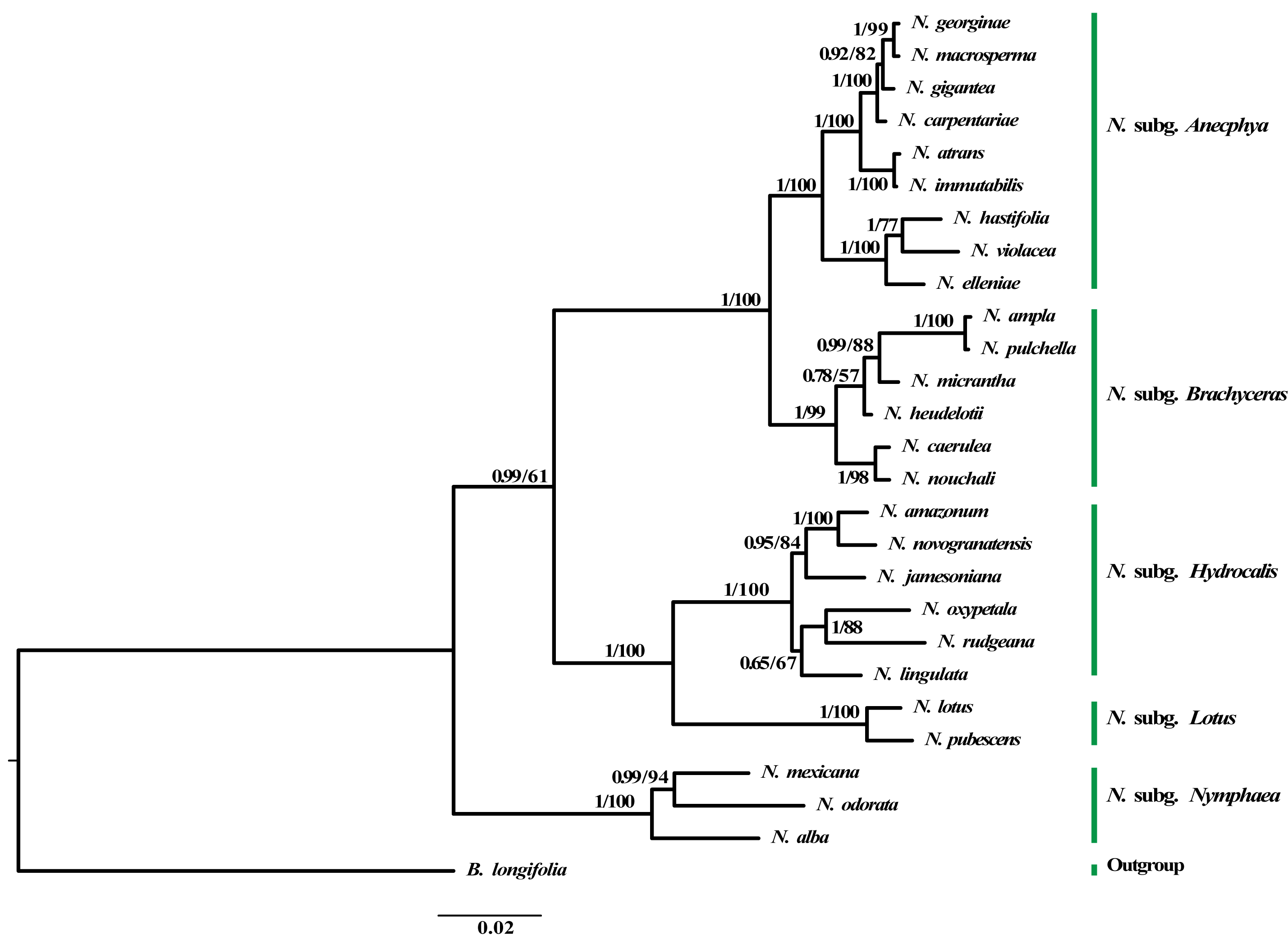

Supplement: Supplementary file 7 — Supplementary Material 7 [file 12870_2024_5141_MOESM7_ESM.tif]

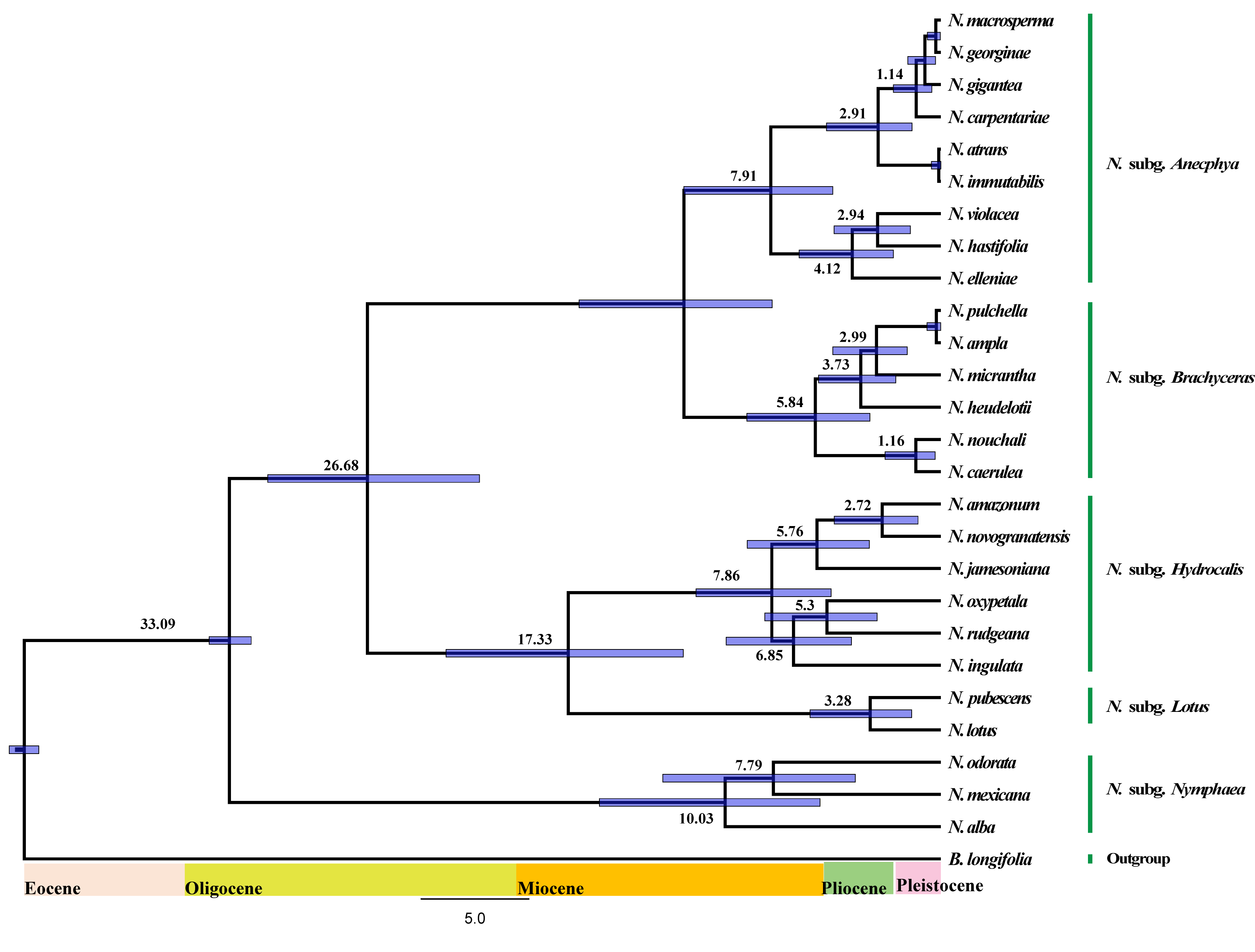

Supplement: Supplementary file 8 — Supplementary Material 8 [file 12870_2024_5141_MOESM8_ESM.tif]

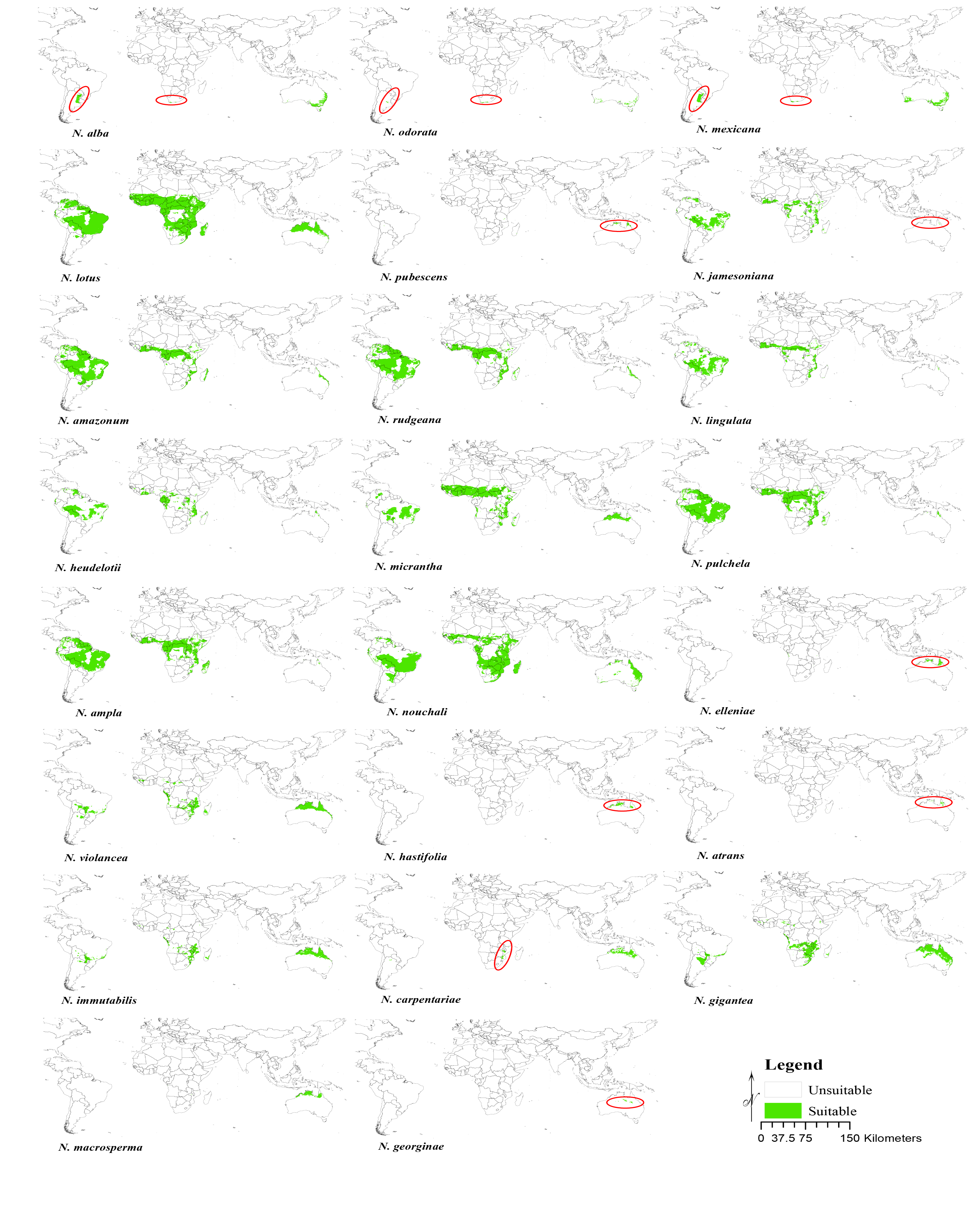

Supplement: Supplementary file 9 — Supplementary Material 9 [file 12870_2024_5141_MOESM9_ESM.tif]
